# An epigenome-wide analysis of DNA methylation, racialized and economic inequities, and air pollution

**DOI:** 10.1186/s13148-025-01929-6

**Published:** 2025-11-27

**Authors:** Sarah Holmes Watkins, Christian Testa, Andrew J. Simpkin, George Davey Smith, Brent Coull, Immaculata De Vivo, Kate Tilling, Pamela D. Waterman, Jarvis T. Chen, Ana V. Diez-Roux, Nancy Krieger, Matthew Suderman, Caroline Relton

**Affiliations:** 1 Population Health Sciences, Bristol Medical School, University of Bristol, Bristol, UK; 2 Integrative Epidemiology Unit, Population Health Sciences, Bristol Medical School, University of Bristol, Bristol, UK; 3 Department of Social and Behavioral Sciences, Harvard T H Chan School of Public Health, Harvard University, Boston, MA 02115 USA; 4 Department of Biostatistics, Harvard School of Public Health, Boston, MA 02115 USA; 5 School of Mathematical and Statistical Sciences, University of Galway, Galway, Ireland; 6 Program in Genetic Epidemiology and Statistical Genetics, Department of Epidemiology, Harvard T.H. Chan School of Public Health, Boston, MA USA; 7 Department of Medicine, Brigham and Women’s Hospital and Harvard Medical School, Boston, MA USA; 8 Department of Epidemiology and Biostatistics and Urban Health Collaborative, Dornsife School of Public Health, Drexel University, Philadelphia, USA; 9 London School of Hygiene & Tropical Medicine, Keppel Street, London, UK

## Abstract

**Background:**

DNA methylation (DNAm) provides a plausible mechanism by which adverse exposures become embodied and contribute to health inequities, due to its role in genome regulation and responsiveness to social and biophysical exposures tied to societal context. However, scant epigenome-wide association studies (EWAS) have included structural and lifecourse measures of exposure, especially in relation to structural discrimination. Our study tested the hypothesis that DNAm is a mechanism by which racial discrimination, economic adversity, and air pollution become biologically embodied, via a series of cross-sectional EWAS, conducted in two population-based samples of US-born Black non-Hispanic (Black NH), white non-Hispanic (white NH), and Hispanic individuals (My Body My Story:: *n* = 224 Black NH and 69 white NH;; and the Multi-Ethnic Study of Atherosclerosis:: *n* = 229 Black NH, *n* = 555 white NH and *n* = 191 Hispanic). Genome-wide changes in DNAm were measured using the Illumina EPIC BeadChip (MBMS; using frozen blood spots) and Illumina 450 k BeadChip (MESA; using purified monocytes).

**Results:**

We observed the strongest associations with traffic-related air pollution (between 0 and 22 DNAm sites associated at *p* < 2.4e-07, measured via black carbon and nitrogen oxides exposure), with evidence from both studies suggesting that air pollution exposure may induce epigenetic changes related to inflammatory processes. However, we did not replicate previous air pollution EWAS findings. We also found suggestive associations of DNAm variation with measures of structural racial discrimination (e.g. for Black NH participants, in MBMS born in a Jim Crow state associates with a DNAm site in ZNF286B at *p* = 8.43E-08; and in MESA adult exposure to racialized economic residential segregation associates with a DNAm site in FUT6 at *p* = 4.05E-08) situated in genes with plausible links to effects on health.

**Conclusions:**

Overall, this work suggests that DNAm is a biological mechanism through which structural racism and air pollution (of which distribution of exposure is inequitable) become embodied and may lead to health inequities. Due to the extensive range of exposures we tested, further replication in additional studies and other tissues is warranted.

**Supplementary Information:**

The online version contains supplementary material available at 10.1186/s13148-025-01929-6.

## Background

Recent advances enabling large population-based epigenetic studies are permitting researchers to test hypotheses linking socially patterned exposures, gene regulation, and health inequities [1–3]. DNA methylation (DNAm) is a plausible biological mechanism by which adverse social exposures may become embodied [4, 5], because 1) it plays an active role in genome regulation [6–8], 2) it changes in response to environmental exposures [1, 9] and internal human physiology like ageing [2] and inflammation [10], and 3) induced changes can be long-lasting [11–14]. There is a growing literature reporting associations between DNAm and environmental factors to which social groups are unequally exposed; a recent review [15] found associations between DNAm and measures of socio-economic position (SEP), including income, education, occupation, and neighbourhood measures; and illustrated timing and duration of exposure are important. Exposure to toxins, including air pollution, is often inequitable between social groups [16, 17]. A number of EWAS have identified associations with particulate matter [18–23] including many individual components of particulate matter such as black carbon [22, 23] and oxides of nitrogen (NOx) [21, 24]; although there is little replication between studies, some studies have failed to find effects of particulate matter [24, 25], NOx [25], and residential proximity to roadways [26]. Four EWAS have found non-overlapping DNAm sites associated with experience of racial discrimination; two DNAm sites in first-generation Ghanaian migrants living in Europe [3], two DNAm sites [27], and one DNAm site [28] in African American women; and DNAm sites in African American (*n* = 4), Hispanic (*n* = 1), and a combination of African American, Hispanic, and white individuals (*n* = 2) [29]. Given population and migration differences, the lack of replication between studies is perhaps not surprising.

However, no EWAS have yet examined associations between DNAm and exposure to racial discrimination and economic adversity, both at individual and structural levels, and measured at different points in the lifecourse, in the same group of people. This is important because it is not clear if the different timing, duration, and levels of these adverse exposures are embodied in different ways involving differing biological pathways. Supporting attention to these issues is a growing body of research documenting how exposure to health-affecting factors such as toxins, quality healthcare, education, fresh food, and green spaces is determined by the way dominant social groups have structured society, which in turn results in health inequities between dominant social groups and groups they subordinate [4, 30]. Structural racism (the totality of ways in which society discriminates against racialized groups [31]), for example, results in groups subjected to racial discrimination often disproportionately bearing the burden of adverse exposures and economic hardship [4, 32], thus driving racialized health inequities [33]. For example, associations between structural racism and cardiovascular health have been shown for discriminatory housing policies and continuing neighbourhood racial segregation [34, 35]; with the historical legacy of slavery [36]; and with state-level institutional domains [37]. Associations have also been shown for diabetes outcomes in the USA [38] and globally [39].

Guided by the ecosocial theory of disease distribution [4, 5], we tested the hypothesis that DNAm is a biological mechanism by which embodiment of structural racial discrimination, economic hardship, and air pollution may occur. We tested our study hypothesis using data from US-born participants in two US population-based studies with similar exposure data: our primary study, the My Body My Story study (MBMS), and the Multi-Ethnic Study of Atherosclerosis study (MESA), which we use for evidence triangulation [40] due to differences between the two study populations.

## Methods

### Participants

This study utilizes biological specimens obtained in 2010–2012 from MBMS and MESA, two US population-based studies that contain similar data on the study exposures. In 2021–2022, the study team newly conducted epigenetic assays for MBMS and added new georeferenced social exposure data. Full study descriptions are in Supplementary materials. Our analyses comprised 293 participants (224 Black and 69 white) from MBMS, recruited from four Community Health Centers (CHCs) in Boston, MA; and 975 participants from MESA who were US-born (229 Black, 555 white NH, additionally including 191 Hispanic), recruited from four of the study field centres (Baltimore, MD; Forsyth County, NC; New York City, NY; and St. Paul, MN).

### Social exposures

We tested the relationship between DNAm and eight variables relating to exposure to racial discrimination (both structural and self-reported), economic hardship, and air pollution; these are described in detail in Table 1. The ecosocial theory of disease distribution posits that “we humans, and all other biological organisms, literally biologically incorporate our societal and ecological context” [4] (p. 32), thereby “producing population distributions of health, disease, and death” [4] (p. 32), including health inequities. From this standpoint, “health inequities comprise the *emergent embodied phenotypes* via which injustice is biologically expressed” [4] (p.40). Since DNAm is both a mechanism of genome regulation and can be influenced by the environment, and our hypothesis was that DNAm is a mechanism by which racial discrimination, economic adversity, and air pollution become biologically embodied. As ecosocial theory has an important historical component, we tested this using exposures measured at a variety of levels and from various points in the lifecourse.

**Table 1:** Detailed description of study exposure variables

Exposure	Description
Childhood exposure to racialized and economic adversity	
Jim Crow birth state	For both MBMS and MESA, this is a true/false variable representing whether or not the participant was born in one of the 21 US states (plus the District of Columbia) which, after the 1865 defeat of the slave-holding Confederacy in the US civil war, undermined the post-civil war Reconstruction efforts to promote racial equality, and brutally enforced white supremacy via state-sponsored legal racial discrimination, disenfranchisement, violence, and terror [41–44]. These regimes remained in force, despite protest, until the Civil Rights movement gained sufficient strength to win passage of the 1964 and 1965 US Civil Rights movement [41–44]. These post-Civil War and pre-Civil Rights states enforcing white supremacy are referred to as “Jim Crow” regimes; although the origins of this phrase are unclear (with some claims, disputed, that the term initially referred to derogatory “blackface” performers), the phrase “Jim Crow” is widely used by scholars of US history to describe the types of law, institutions, and social practices enforcing white supremacy via state power and both judicial and extra-judicial violence [41–44]. In addition to DC, the 21 Jim Crow states comprised, alphabetically: Alabama; Arizona; Arkansas; Delaware; District of Columbia; Florida; Georgia; Kansas; Kentucky; Louisiana; Maryland; Mississippi; Missouri; New Mexico; North Carolina; Oklahoma; South Carolina; Tennessee; Texas; Virginia; West Virginia; and Wyoming [41]
Parent’s highest education	For both MBMS and MESA, this was measured using three categories: < high school; > high school and < 4 years college; > 4 years college (reference level)
Participant’s highest education	For both MBMS and MESA, this was measured using three categories: < high school; > high school and < 4 years college; > 4 years college (reference level)
Adult exposure to racialized and economic adversity	
Household poverty to income ratio	For both MBMS and MESA, this measure is derived from participants’ ratio of household income in 2010 dollars to the US 2010 poverty line given their household composition
Index of Concentration at the Extremes for racialized economic segregation	For both MBMS and MESA, this measure captures extremes of privilege and deprivation, measuring both economic and racialized segregation at the census tract level [45]. Score ranges from -1 to 1 representing 100% non-Hispanic Black residents among the lowest 2 census wealth brackets, to 100% non-Hispanic white residents among the 2 highest wealth brackets
Black carbon exposure	MBMS: measured as the cumulative average exposure to ambient black carbon (in μg/m3) at the longitude–latitude of their residential address for the 1-year prior to the individual’s study enrolment (2008–2010) [16] MESA: measured as light absorption coefficient (in 10^–5^/m; 1 unit approx. equivalent to 1 µg/m3) for the addresses lived at during the year prior to exam 5 (when blood samples used for DNAm measurement were taken) [46]
NOx exposure	MBMS: measured as Pollution Proximity Index (https://datacommon.mapc.org/calendar/2020/july), which uses NOx measurements to construct a weighted score of roadway pollution, measured on a scale of 0–5, with measurements taken over the year 2012 MESA: NOx was measured in parts per billion for the year prior to exam 5 (when blood samples used for DNAm measurement were taken) [46]
Experiences of discrimination (EOD) (MBMS only)	A validated self-report questionnaire measuring domains of exposure to racial discrimination. Score range 0–9, categorized into 0, 1–2, 3 + based on previous work indicating a nonlinear relationship; the reference level of this exposure was 1–2 [47]
Major discrimination scale (MDS) (MESA only)	A validated self-report questionnaire measuring domains of exposure to racial discrimination; combined with the attribution aspect from EDS (everyday discrimination scale) to enable comparability between EOD and MDS. Score range 0–5, categorized into 0, 1–2, 3 + based on previous work indicating a nonlinear relationship: the reference level of this exposure was 1–2 [47]

### DNA methylation

For MBMS, DNA was extracted from frozen blood spots in 2021 and bisulphite converted with the EZ DNA Methylation-Lightning™ Kit (Zymo Research) according to the manufacturer’s instructions. The eluant from the bisulphite-converted DNA was then applied to the Illumina Infinium MethylationEPIC Beadchip to measure DNA methylation, according to the manufacturer’s protocol. The EPIC BeadChips were scanned using Illumina iScan, with an initial quality review conducted with GenomeStudio. Sample QC and normalization were conducted using the pipeline implemented in the *meffil* R package, which has previously been described in detail [48]. DNA methylation is reported in beta values; this measures methylation on a scale of 0 (0% methylation) to 1 (100% methylation).

DNA extraction and processing has previously been described in detail for MESA [49], but briefly, in 2012–2013 DNA was extracted from purified monocytes and bisulfite converted using the EZ-96 DNA MethylationTM Kit (Zymo Research, Orange, CA, USA). The eluant from the bisulphite-converted DNA was then applied to the Illumina Infinium HumanMethylation450 BeadChip, according to the manufacturer’s protocol. The BeadChips were scanned using the Illumina HiScan reader and summarized using GenomeStudio. Sample normalization was conducted using the R package lumi, with QC checking for “sex and race/ethnicity” mismatches, and outlier identification. We converted the methylation M-values provided by MESA to beta values using the *m2beta* function in the R package *lumi* [50].

Because frozen blood spots are whole blood samples, they contain a multitude of blood cell types; resultantly MBMS DNAm data reflect a composite measure across cell types that have differing DNAm profiles. This is likely to result in averaging of DNAm across cell types at many loci. In contrast, MESA data are from a purified cell type (monocytes) that typically comprise 10% of whole blood; MESA DNAm data will reflect DNAm levels in monocytes only. Thus, although we might expect some comparability between the DNAm measurements, there will be differences between the methylation profiles that arise from differing DNAm profiles of the cell types. As EWAS examine associations at single loci, there is no way to increase comparability between the datasets; but we see this as an opportunity for evidence triangulation, using differing data sources to strengthen inferences [40]. It may be that associations are easier to detect in MESA because signal will not be lost via averaging over multiple cell types.

### Participant stratification

EWAS were stratified by self-reported membership of racialized groups, for two reasons. Firstly, for most of our exposures, different constructs are represented between the racialized groups; for example, being born in a Jim Crow state means something very different for individuals who identify as Black versus white. Secondly, stratification prevents potential confounding by racialized group due to exposure and a degree of genetic differences between groups. Racialized groups are social constructs that are changeable and dependent on local context [51]; they are important to our research question because group membership is pertinent to the experience of social inequities perpetuated by structural racial discrimination.

### EWAS

All EWAS were conducted using linear regression models implemented using the R package *meffil* [48]. Many exposures had low levels of missing data, and complete case numbers for each EWAS can be found in Table 4. For EWAS in both MBMS and MESA, we adjusted for age, reported gender in MBMS and sex in MESA (which differ here as we utilize the terminology used in the self-report questionnaires for each study), smoking status, blood cell count proportions (estimated in meffil using the “blood gse35069 complete reference” [52]), and batch effects (estimated using surrogate variables [SVs] calculated using the R package *sva* [53]). We used SVs to adjust for batch rather than batch variables directly due to small cell frequencies (due to stratifying the dataset). We chose to use 5 SVs for MBMS and 10 for MESA as these accounted for the majority of batch variation and, in MBMS, DNA input level. Their inclusion in EWAS models shifted EWAS lambda values to approximately 1. EWAS p value thresholds were adjusted for multiple tests as recommended in the literature: as MBMS utilized the EPIC array with 850 k features, the threshold was 9e-08 [54]; as MESA utilized the 450 k array, the threshold was 2.4e-07 [55]. Categorical exposures with more than two levels (parent’s highest education, participant’s highest education, experiences of discrimination) were run as multiple binary EWAS, with each level compared to a pre-defined reference group. (Reference groups are identified from previous work and are detailed in Table 1 in the main text.)

### Replication

We did not perform meta-analyses of MBMS and MESA, because the substantial differences between the two cohorts (involving age range, demographics, geographic location, cell type, and array) would make direct comparisons too difficult to interpret. We did, however, conduct replication tests to explore consistency in the associations in both cohorts. Firstly, we took the top 25 sites associated with each exposure in the MBMS EWAS that were also present on the 450 k array and assessed them for association in the MESA EWAS at the equivalent of *p* < 0.05/25. Secondly, we correlated effect sizes (using Pearson correlation) between MBMS and MESA, for the top 10, 25, 50, 100, and 200 sites identified in each exposure EWAS. All but one of our exposures were measured either in exactly the same way or were linearly equivalent between MBMS and MESA; Pollution Proximity Index in MBMS and NOx in MESA both utilize NOx measurement (see Table 1) so are using measures of the same exposure. However, they are measured on differing scales, and Pollution Proximity Index was developed to specifically target traffic emissions, which could impact the ability to detect replication via correlation. Correlation of effects between studies is a recognized way to evaluate consistency of effect directions between studies. However, we note that a high correlation between studies does not imply consistent effect sizes, and, due to the inevitable errors in the calculated effects, the calculated correlation will be downwardly biased [56]. Because correlation can be influenced by outlying values, we used robust regression to validate the robustness of correlating effect sizes (using rlm from the R package MASS [57]). Finally, we conducted a binomial test for each MBMS EWAS to ascertain whether the top 10, 25, 50, 100, and 200 sites replicated in MESA at *p* < 0.05 with the same direction of effect. We also used these three tests of replication to assess whether sites identified in the MESA EWAS replicated in MBMS. (Table 2)

**Table 2:** Characteristics of MBMS and MESA participants

Variable			MBMS: Black NH	MBMS: white NH	MESA: Black NH	MESA: white NH	MESA: Hispanic
Total N			224	69	229	555	191
Sociodemographic characteristics							
Age: mean (SD)			49.02 (7.8)	48.7 (8.3)	71 (8.9)	70.1 (9.5)	68.5 (8.9)
Gender: N (%) women			135 (60.3%)	49 (71%)	133 (58.1%)	264 (47.6%)	86 (45%)
BMI: mean (SD)			32.1 (7.7)	29.7 (7.2)	30.6 (5.7)	28.7 (5.3)	30.8 (5.5)
Smoking: N (%)	Current		115 (51.3%)	24 (34.8%)	31 (13.7%)	44 (8%)	16 (8.6%)
	Former		31 (13.8%)	23 (33.3%)	101 (44.9%)	262 (47.7%)	80 (43%)
	Never		78 (34.8%)	22 (31.9%)	93 (41.5%)	243 (44.3%)	90 (48.4%)
	*Missing*		*0*	*0*	*4 (1.7%)*	*6 (1.1%)*	*5 (2.6%)*
Childhood exposure to racialized and economic adversity:							
Born in a Jim Crow state^1^: N (%) yes			71 (31.7%)	2 (3%)	165 (72.1%)	166 (29.9%)	19 (9.9%)
Parent’s highest education: N (%)	< High school		29 (18.4%)	8 (14%)	95 (42.2%)	161 (29.3%)	129 (69.7%)
	> = High school and < 4 yr college		94 (59.5%)	24 (42.1%)	106 (47.3%)	258 (47%)	51 (27.6%)
	4 + years college		35 (22.2%)	25 (43.9%)	24 (10.7%)	130 (23.7%)	5 (2.7%)
	*Missing*		*66 (29.5%)*	*12 (17.4%)*	*4 (1.7%)*	*6 (1.1%)*	*6 (3.1%)*
Participant’s education: N (%)	< High school		34 (15.2%)	8 (11.6%)	23 (10%)	21 (3.8%)	35 (18.3%)
	> = High school and < 4 yr college		161 (71.9%)	33 (47.8%)	175 (76.4%)	413 (74.4%)	140 (73.3%)
	4 + years college		29 (12.9%)	28 (40.6%)	31 (13.5%)	121 (21.8%)	16 (8.4%)
	*Missing:*		*0*	*0*	*0*	*0*	*0*
Adult exposure to racialized and economic adversity:							
Household income to poverty ratio^2^: mean (SD)			2.2 (2.2)	2.9 (2.3)	3.9 (2.3)	4.8 (2.9)	3.3 (2.1)
*Missing*			*34 (15.2%)*	*3 (4.3%)*	*9 (3.9%)*	*24 (4.3%)*	*7 (3.7%)*
Index of Concentration at the Extremes for racialized economic segregation^3^: mean(SD)			-0.07 (0.2)	0.19 (0.2)	-0.11 (0.2)	0.16 (0.2)	0.09 (0.2)
*Missing*			*0*	*0*	*2 (0.9%)*	*4 (0.7%)*	*12 (6.3%)*
Black carbon (μg/m3): mean (SD)			0.64 (0.1)	0.63 (0.17)			
*Missing*			*0*	*0*			
Light absorption coefficient (10^–5^/m): mean (SD)					0.89 (0.35)	0.6 (0.3)	0.7 (0.4)
*Missing*					*14 (6.1%)*	*23 (4.1%)*	*11 (5.6%)*
Pollution Proximity Index^4^ (scale of 0–5): mean (SD)			4.3 (1.1)	3.9 (1.4)			
*Missing*			*5 (2.2%)*	*0*			
Oxides of nitrogen (NOx, parts per billion): mean (SD)					31.9 (16.2)	21.55 (12.2)	27 (16.4)
*Missing*					*14 (6.1%)*	*23 (4.1%)*	*11 (5.6%)*
Experiences of Discrimination (EOD, N of domains)^5^: N (%)		0	30 (13.4%)	35 (50.7%)			
		1–2	52 (23.2%)	24 (34.8%)			
		3 +	140 (62.5%)	10 (14.5%)			
		*Missing*	*2 (0.9%)*	*0*			
Major Discrimination Scale (MDS, N of domains)^6^: N (%)		0			129 (56.6%)	534 (96.4%)	131 (68.6%)
		1–2			79 (34.6%)	20 (3.6%)	53 (27.7%)
		3 +			20 (8.8%)	0	7 (3.7%)
		*Missing*			*1 (0.4%)*	*1 (0.2%)*	*0*

### Sensitivity analysis

In MESA, we conducted a sensitivity analysis to test whether our results were influenced by population stratification, by additionally adjusting each EWAS for the first 10 genetic principal components (PCs), and then using Pearson correlation to test effect size replication for the top 10, 25, 50, 100, and 200 sites in each EWAS. Details of MESA genomic data are in Supplementary materials. Additional sensitivity analysis restricted the MESA analysis to participants recruited from the Baltimore and New York sites, because these cities bear the greatest similarity to the Boston area in terms of geographical location, city environment, and social histories.

### Meta-analysis

We meta-analysed associations with air pollution within MBMS and within MESA because air pollution is the only exposure we tested that we would hypothesize to have the same meaning, and therefore biological effect, for all individuals. We used *METAL* [58] to conduct fixed effects meta-analysis of the

 effect sizes and standard errors of the EWAS summary statistics of each racialized group, for black carbon/LAC and NOx.

### Biological enrichments of top sites

For DNAm sites associated with an exposure below the genome-wide threshold, we used the UCSC genome browser to identify genomic regions. For sites within known genes, we used GeneCards (https://www.genecards.org/) and literature searches to identify putative gene functions. For all EWAS, we performed analyses to ascertain whether DNAm sites associated with our exposures indicate effects on particular biological pathways, processes or functions. These analyses included the top 100 sites from each EWAS to ensure were sufficient numbers to assess enrichment. Gene set enrichment analyses were conducted using the R package *missMethyl *[59]. Enrichments among sites previously associated with other phenotypes and exposures were tested by comparison to published EWAS summary statistics recorded by the EWAS catalog [60] using previously described methods to create phenotype and exposure categories [61]. Enrichments for tissue-specific chromatin states, genomic regions, and transcription factor binding sites (TFBS) were assessed the R package *LOLA* [62]. Specific details are in Supplementary materials.

#### Lookup of associations in a priori specified genomic locations

We hypothesized a priori that our EWAS would detect DNAm sites that have been robustly associated with our study exposures, or factors that might relate to our exposures, in previous EWAS studies. DNAm sites associated with these domains in previous literature were identified through the EWAS catalog [60] and literature searches. To ensure the sites from previous studies that we tested had robust associations with our exposures, we only took previous DNAm sites forward where they were identified in at least two separate studies. However, for racial discrimination we took previous sites identified by just one previous study, as this area has limited literature and is an important focus of our study. EWAS results for each of the tested exposures were then reduced to the sites that had been identified for that particular exposure in the previous literature, and tested for association at *p* < 0.05 divided by the number of sites tested. Please see Supplementary materials for further details.

## Results

### Participant characteristics

Both cohorts include racialized groups that are underrepresented in epigenetic studies. Beyond this, substantial differences existed between the racialized groups within and across MBMS and MESA. Overall, MBMS participants were on average 21 years younger than MESA participants, had less variability in exposure to air pollution, and far more were current smokers. In both studies, Black NH compared to white NH participants had higher BMI, rates of smoking, impoverishment, lower education, rates of self-reported exposure to racial discrimination and were more likely to be born in a Jim Crow state and live in a neighbourhood with extreme concentrations of low-income persons of colour. In MESA, Hispanic participants reported the lowest levels of personal and parental education. In MBMS, there were differences between the predicted cell type proportions of neutrophils and eosinophils.

^1^ Jim Crow states are the 21 US states (plus the District of Columbia) which permitted legal racial discrimination prior to the 1964 US Civil Rights Act.

^2^ Participants’ ratio of household income in 2010 dollars to the US 2010 poverty line given household composition.

^3^ Census tract measure of economic and racialized segregation, scored from -1 to 1.

^4^ NOx measurements were used to construct a weighted score of roadway pollution.

^5^ Validated self-report questionnaire measuring the number of domains of exposure to racial discrimination. Score range 0–9, categorized into 0, 1–2, 3. The reference level of this exposure was 1–2.

^6^ Validated self-report questionnaire measuring the number of domains of exposure to racial discrimination; combined with the attribution aspect from EDS (everyday discrimination scale) to enable comparability between EOD and MDS. Score range 0–5, categorized into 0, 1–2, 3 + . The reference level of this exposure was 1–2.

### EWAS results and biological interpretation

**Fig. 1:**
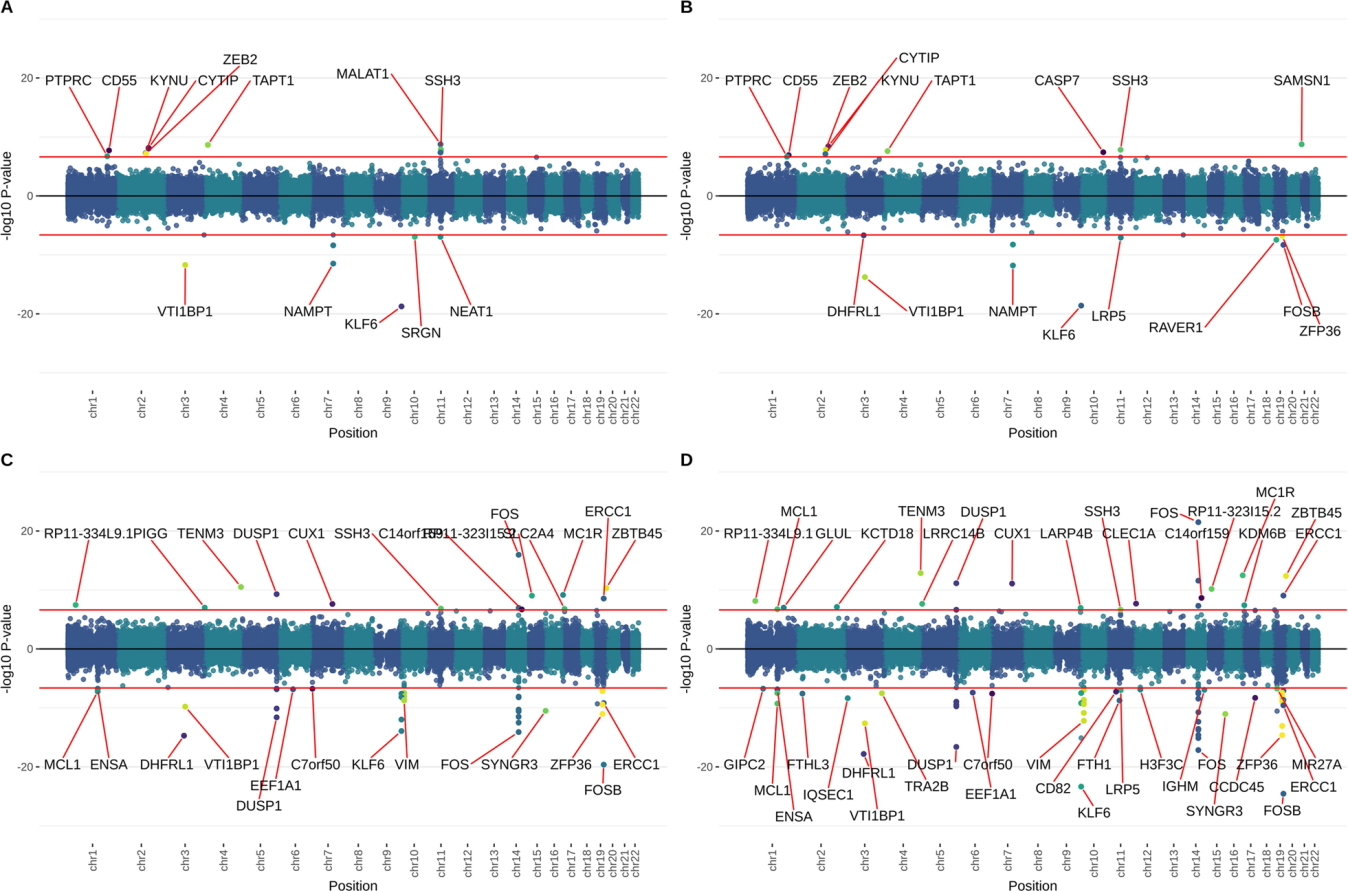
MESA air pollution meta-analysis Miami plots. **A**: MESA full cohort LAC meta-analysis. **B**: MESA full cohort NOx meta-analysis. **C**: MESA subgroup LAC meta-analysis. **D**: MESA subgroup NOx meta-analysis

In MBMS, among the Black NH participants one DNAm site, in *ZNF286B*, was associated with being born in a Jim Crow state. Another DNAm site, *PLXND1*, was associated with participants having less than high school education. Among white NH participants, no associations passed the genome-wide threshold. See Table 3 for details of gene functions; Table 4 for numbers of associated EWAS sites; and Supplementary Figs. 1 and 2 for Miami plots and Supplementary Figs. 9 and 10 for QQ plots for each of the exposures. In MESA, two DNAm sites were associated with racialized economic segregation — one in Black NH participants (in *FUT6*) and one in white NH participants (a CpG previously associated with BMI); and in Black NH participants one DNAm site (in *PDE4D*) was associated with an MDS score of 0. The majority of associations in MESA were related to air pollution exposure—among Black NH participants, 12 sites with LAC and 22 sites with NOx. Notably, many of these sites are clustered in or near regulatory regions of genes with putative roles in immune responses and are known to interact with one another, including *KLF6*, *MIR23A*, *FOS*, *FOSB*, *ZFP36,* and *DUSP1*. Among the MESA white NH participants, four DNAm sites were associated with both LAC and NOx, and an additional 3 uniquely associated with LAC. Associations of 53 DNAm sites with birth in a Jim Crow state were very likely the result of mediation by air pollution. (Evidence for this hypothesis can be found in Supplementary materials section “EWAS results and biological interpretation”.) Among Hispanic participants, one site was associated with LAC (*NPNT*) and one with NOx (*ADPRHL1*). See Table 4 for numbers of associations for all EWAS performed; Supplementary Figs. 3–5 for corresponding Miami plots and Supplementary Figs. 11 to 13 for QQ plots; and Supplementary Table 6 for a complete list of sites passing the genome-wide threshold. EWAS lambda values were close to 1 for the majority of analyses, with the highest value at 1.23—lambdas are displayed on the QQ plots in the supplement. (Fig. 1)

**Table 3:** Putative functions of genes in which the top exposure-associated DNAm sites sit; with details of how many sites within that gene were identified, and in which main analysis EWAS they were identified

Gene	Functional relevance	Chr	Exposure	Dataset	CpG	Effect size	P value
*ZNF286B*	A pseudogene, which is predicted to be involved in regulation of RNA polymerase 2 (Pol II)-mediated transcription (Pol II transcribes protein-coding genes into mRNA [63])	17	Born in a Jim Crow state	MBMS: Black NH	cg26306683	-0.00226	8.43E-08
*PLXND1*	Encodes a cell receptor involved in axonal guidance, migration of endothelial cells, and regulates atherosclerotic plaque deposition [64]	3	< HS education	MBMS: Black NH	cg22555181	0.015822	5.85E-08
*FUT6*	A Golgi stack membrane protein that is involved in basophil-mediated allergic inflammation [65]	19	residential racialized economic segregation	MESA: Black NH	cg01630130	-0.01286	4.05E-08
*KLF6*	A transcriptional activator and tumour suppressor, which regulates macrophage inflammatory responses [66]	10	NOx	MESA: Black NH	cg27283993	-0.00093	2.18E-13
					cg14357508	-0.00046	2.79E-08
				MESA: white NH			
			LAC	MESA: Black NH	cg27283993	-0.03741	4.93E-10
					cg19347588	-0.05152	1.23E-09
				MESA: white NH	cg19347588	-0.011985	1.16E-12
*FOS*	FOS is an early response gene, and is a subunit of the AP-1 transcription factor complex, which regulates gene expression involved in lung injury, repair and transformation [67], as well as regulating many cytokine genes and T-cell differentiation [68, 69]	14	NOx	MESA: Black NH	cg00773696	-0.000228	5.05E-09
					cg04305870	-0.000321	2.55E-08
					cg03507218	-0.000562	6.67E-08
					cg18117039	-0.000837	7.11E-08
					cg27019030	-0.001136	9.73E-08
					cg07159858	-0.000619	1.08E-07
					cg18717355	-0.000442	1.41E-07
			LAC	MESA: Black NH	cg00773696	-0.009837	8.52E-08
*FOSB*	FOSB is another subunit of AP-1	19	NOx	MESA: Black NH	cg20646490	-0.000872	2.58E-11
			LAC	MESA: Black NH	cg20646490	-0.038856	2.52E-10
*ZFP36*	ZFP36 encodes a protein (TTP) that is a key regulator of post-transcriptional regulation, which has roles in immune and inflammatory responses [70]	19	NOx	MESA: Black NH	cg14890578	-0.000393	3.78E-08
					cg25739316	-0.000180	1.25E-07
*DUSP1*	DUSP1 is a gene that regulates airway inflammation; DUSP1’s key mechanism of inflammation modulation may be via modulating the actions of the protein TTP encoded by the ZFP36 gene [71]	5	NOx	MESA: Black NH	cg12333707	-0.000702	9.10E-10
			LAC	MESA: Black NH	cg12333707	-0.028484	1.49E-07
*VIM*	Encodes a filament protein responsible for integrity of cell shape and cytoplasm. Pathogens can attach to this protein on the cell surface. Putative involvement regulating innate immune response to lung injury and irritation [72]	10	NOx	MESA: Black NH	cg14898116	-0.000373	5.39E-09
*MALAT1*	Metastasis associated lung adenocarcinoma transcript 1, lncRNA that acts as transcriptional regulator; upregulation linked to cancerous tissues and proliferation and metastasis of tumour cells	11	LAC	MESA: white NH	cg26489875	0.0330592	1.14E-10
*CYTIP*	Modulates activation of ARF (ADP-ribosylation factor) genes, which regulate vesicle budding, tethering and cytoskeleton organization. Dysregulation of ARFs may be involved in cancer cell migration and invasion	2	NOx	MESA: white NH	cg01715901	0.0006371	2.31E-08
			LAC	MESA: white NH	cg01715901	0.0275629	7.01E-09
*ZEB2*	DNA-binding transcriptional repressor involved in the transforming growth factor-β (TGF-β) signalling pathway that interacts with activated SMADs. May be related to small cell lung cancer [73]	2	NOx	MESA: white NH	cg20995564	0.0005788	1.09E-07
			LAC	MESA: white NH	cg20995564	0.0244766	7.80E-08
*PTPRC*	A receptor-type PTP that is an essential regulator of T- and B-cell antigen receptor signalling	1	NOx	MESA: white NH	cg15626828	0.0005196	2.31E-08
			LAC	MESA: white NH	cg15626828	0.0219937	1.51E-08
*NPNT*	An extracellular matrix protein that has roles in kidney development and carcinogenesis [74]	4	LAC	MESA: Hispanic	cg01852585	0.0184083	2.27E-07
*ADPRHL1*	a protein encoding a pseudoenzyme involved in cardiogenesis [75]	13	NOx	MESA: Hispanic	cg12637085	-0.001030	2.14E-08
*PDE4D*	PDE4s, including PDE4D, have roles in cell signalling, as well as regulating inflammatory responses [76]	5	MDS	MESA: Black NH	cg10487428	0.017243	6.57E-08

**Table 4:** Summary of the number of DNAm sites passing the genome-wide threshold in each individual EWAS in MBMS (threshold 9e-8) and MESA (threshold 2.4e-7)

	MBMS				MESA					
	Black NH		white NH		Black NH		white NH		Hispanic	
	N	N sites	N	N sites	N	N sites	N	N sites	N	N sites
Birth in a Jim crow state	224	1	NA^1^	NA^1^	225	0	549	53^2^	186	0
Parent’s highest education (high vs low)	64	0	33	0	117	0	288	0	NA^3^	NA^3^
Parent’s highest education (high vs mid)	129	0	49	0	128	0	384	0	NA^3^	NA^3^
Participant’s education (high vs low)	63	1	36	0	54	0	142	0	51	0
Participant’s education (high vs mid)	190	0	61	0	202	0	534	0	156	0
Household poverty to income ratio	190	0	66	0	218	0	528	0	180	0
Racialized economic segregation	224	0	69	0	223	1	545	1	174	0
Black carbon	224	0	69	0	211	12	526	7	175	1
Nitrogen oxides	219	0	69	0	211	22	526	4	175	1
EOD^5^ (1–2 vs 0)	82	0	59	0						
EOD^5^ (1–2 vs 3 +)	192	0	34	0						
MDS^6^ (1–2 vs 0)					204	1	554	0	184	0
MDS^6^ (1–2 vs 3 +)					97	0	NA^4^	NA^4^	60	0

The list of specific DNAm sites passing the genome-wide threshold can be found in Supplementary Table 4. ^1^The EWAS was not run for Jim Crow birth state for white NH participants in MBMS, due to small cell numbers. ^2^See text; these 53 sites were driven by air pollution differences between individuals born and not born in a Jim Crow state. ^3^ The two EWAS for parental education were not run for Hispanic participants in MESA, due to small cell numbers.^4^ The EWAS was not run for MDS (score of 1–2 vs 3 +) for white NH participants in MESA, as no participants had a score of 3 or more. ^5^ EOD—Experiences of Discrimination scale. ^6^ MDS—Major Discrimination Scale

### Replication

We assessed replication between equivalent exposures in MBMS and MESA in three ways. Firstly, for both MBMS and MESA we tested replication of the top 25 EWAS hits at *p* < 0.05/25 in the other dataset, for each of the exposures. We found one site identified in MESA that associated with less than high school education in White non-Hispanic participants replicated in MBMS at p = 0.0006. We additionally considered the top 10 and 50 sites and found similar results.

Secondly, we examined the correlation between EWAS effect sizes. In the Black non-Hispanic participants, we found some evidence that effects found in the MBMS EWAS of parental education (< high school vs 4 + years of college education) replicated in MESA (for 3 of 5 sets of sites, Pearson’s R = 0.79 to 0.45, p = 0.0009 to 0.007). When looking at EWAS effects found in MESA, we found evidence of negative correlation in MBMS for the EWAS of household income to poverty ratio (for 4 of 5 sets of sites, Pearson’s R = − 0.62 to − 0.3, *p* = 1.5e-5 to 0.00084), and less than high school education (for 4 of 5 sets of sites, Pearson’s R = 0.88 to 0.49, *p* = 1.2e-7 to 0.00087). In the White non-Hispanic participants, we found convincing evidence of positive correlation between effect sizes found in MBMS EWAS, and the effect sizes in MESA, for household poverty to income ratio (for all 5 sets of sites, Pearson’s R = 0.76 to 0.16,p = 0.0024 to 0.041). There was some evidence of negative correlation between effect sizes for the top sites in the MESA EWAS, and the effect sizes in MBMS, for nitrous oxides (for 3 of 5 sets of sites, Pearson’s R = − 0.53 to − 0.31, p = 3.2e-8 to 7.9e-5), and less than high school education (for 2 of 5 sets of sites, Pearson’s R = 0.82 to 0.61, p = 0.0012 to 0.0035).

Thirdly, we asked if direction of effect was preserved in replication data for the top associated sites. Here, we found some evidence of replication for sites associated with less than high school education in white NH MESA participants (3 of 5 sets of sites, p = 0.02 to p = 0.03).

### MESA subgroup analysis

The main impact of removing the Minnesota and Forsyth County sites (which both had very low levels of air pollution) was to remove the confounding structure between air pollution and Jim Crow birth state among white NH participants, reducing the number of DNAm sites associated with Jim Crow birth state to zero. Please see Supplementary materials section “EWAS results and biological interpretation” for details. It also increased the similarity of air pollution associations between the Black NH and white NH participants; for example, of the 19 DNAm sites associated with NOx among white NH participants, 12 passed the genome-wide threshold in the Black NH participant EWAS, and correlations between the top 10–200 effect sizes became strong to moderate (ranging from -0.51 to 0.85 in the main analysis, to 0.4 to 0.97 in the subgroup analysis—see Supplementary materials section “MESA subgroup analysis” for a full table). Numbers of associated sites in all subgroup EWAS are in Table 5. Miami plots for this MESA subgroup can be found in Supplementary Figs. 6 to 8, and QQ plots are in Supplementary Figs. 14 to 16.

**Table 5:** Summary of EWAS results for MESA subgroup analysis

	Black NH		White NH		Hispanic	
	N	N sites	N	N sites	N	N sites
Birth in a Jim Crow state	221	0	237	0	NA^1^	NA^1^
Parent’s highest education (high vs low)	115	0	134	0	NA^2^	NA^2^
Parent’s highest education (high vs mid)	125	0	164	0	NA^2^	NA^2^
Participant’s education (high vs low)	54	0	55	0	NA^2^	NA^2^
Participant’s education (high vs mid)	198	0	227	0	NA^2^	NA^2^
Household poverty/income ratio	214	0	227	1	67	0
Racialized economic segregation	219	1	233	0	59	0
Light Absorption Coefficient	208	10	231	6	57	0
Nitrogen oxides	208	20	231	19	57	0
Major discrimination scale (1–2 vs 0)	200	1	236	0	67	0
Major discrimination scale (1–2 vs 3 +)	94	0	NA^3^	NA^3^	NA^3^	NA^3^

^1^ The EWAS for Jim Crow birth state were not run for Hispanic participants due to small cell numbers. ^2^ The EWAS for parental and participant education were not run for Hispanic participants, due to small cell numbers. ^3^ The EWAS were not run for MDS (score of 1–2 vs 3 +) for white NH and Hispanic participants in MESA, as no participants had a score of 3 or more

### Air pollution meta-analysis

Meta-analysis in MBMS (black carbon n = 293, NOx n = 288) did not yield any sites passing the genome-wide threshold. In MESA (both n = 912), we see approximately similar numbers of associations as with the Black NH subgroup (17 for LAC and 18 for NOx); see Supplementary Table 3. When we restricted to participants recruited at the Baltimore and New York sites (n = 496), a much larger number of DNAm sites passed the genome-wide threshold (51 for LAC and 79 for NOx). Effect sizes correlated strongly between the full cohort meta-analysis and the subset meta-analysis for NOxNOx (Pearson’s correlation R = 0.84–0.92), and moderately to strongly for LAC (0.4–0.91); but p values do not bear a consistent relationship with each other so this is not simply a thresholding effect (please see Supplementary materials for tables of correlations, and Supplementary Table 7 for a table comparing p values and effect sizes). This difference may be due to Minnesota and Forsyth County sites having had very low variance in pollution levels which enabled better detection of effects due to removal of low variance observations; alternately it may be due to differing composition of pollutants at different geographical sites The MESA sensitivity meta-analysis identified multiple associations linked to *DUSP1*, *FOS*, *KLF6*, *MCL1*, and *VIM* –—genes that have putative roles in inflammation and immunity. A table of all meta-analysis sites passing the genome-wide threshold is in Supplementary Table 8.

### Biological enrichments of exposure associations

#### Gene ontology

We observed no evidence for gene set enrichments for any Gene Ontology terms among the top 100 sites of the main EWAS we conducted. However, we did observe that the 22 sites associated with NOx above the genome-wide threshold among MESA Black NH participants were enriched for the gene ontology terms “response to glucocorticoid” and “response to corticosteroid” (FDR > 0.05). We also observed that the MESA meta-analysis of NOx among all participants was associated with 13 Gene Ontology terms (FDR > 0.05) related to blood-based immune response.

#### EWAS catalog

We observed a number of relevant enrichments among sites identified in our EWAS (as defined by an association at p < 0.05, using Fisher’s exact test). In MBMS, we see enrichment for inflammation for both NOx and LAC EWAS among Black NH participants, and in the NOx meta-analysis. Among the full sample of US-born MESA participants, the LAC and NOx EWAS were enriched for infection and cancer among both Black NH and white NH participants; the NOx EWAS were enriched for metabolic factors among MESA Hispanic participants; and the LAC and NOx meta-analyses were both enriched for lung, inflammation, diet, cancer, and alcohol. When we restricted MESA to participants recruited at the New York and Baltimore sites, we observed consistent enrichment for infection in Black NH and white NH individuals; with enrichment for inflammation in Hispanic individuals; and in the meta-analyses, for LAC we observed enrichment for infection and cancer, and for NOx we observed enrichment for infection.

We also found some consistent enrichments for measures of structural discrimination. Among both MESA Black NH and Hispanic participants, the racialized economic segregation EWAS were enriched for neurological traits. Among both the white NH and Hispanic participants, household poverty to income ratio EWAS was enriched for SEP and education. In the MESA subgroup analysis, enrichment for prenatal exposures was observed for the Jim Crow birth state EWAS among the Black NH and Hispanic participants. A table of all associations is in Supplementary Table 5, and forest plots of the associations are in Supplementary Figs. 17–27.

### Enrichment for genomic features

When we looked at enrichment of genomic locations of the top 100 sites (*p* < 0.05, Fisher’s exact test), we found that among MBMS Black NH participants, NOx was the only exposure with associated CpGs being located in active genomic regions (two chromatin states, bivalent promoter and promotor upstream of transcription start sites; in addition to being located in promoters, CpG islands and CpG island shores; and enrichment for 9 TFBS). In the MBMS meta-analyses, NOx was enriched for regions related to gene promoters. Among MESA Black NH participants, we observe enrichment for regions related to transcription and genome regulation in the LAC and NOx EWAS. We also observed enrichment relating to transcription regulation for the birth in a Jim Crow state EWAS. Among MESA white NH participants, we observed enrichment for transcription regulation for both measures of air pollution. Among MESA Hispanic participants, LAC exposure shows some associations with active genomic regions. When we restrict MESA to the New York and Baltimore sites, we see a similar set of enrichments; and in the MESA meta-analyses we see consistent enrichment related to transcription regulation and promotors. Notably, genomic feature enrichments for NOx among both MBMS and MESA Black NH participants involved similar genomic locations (CpG islands and shores) and chromatin states (related to promotors), as well as 6 of a possible 9 TFBS. Please see Supplementary materials for a more detailed discussion.

Lookup of associations in a priori specified genomic locations.

We did not observe any associations in our EWAS results for sites identified in previous EWAS of related exposures.

## Discussion

The series of EWAS we conducted on a range of adverse exposures at different levels and at different points in the lifecourse, drawing on two different population-based studies with similar exposure data, provide evidence that DNAm may be a biological pathway by which societal context shapes health inequities. This work has shown for the first time associations between DNAm and multiple levels of structural discrimination. These associations sit within genes that are biologically plausible routes of embodiment involving gene regulation, including inflammation; this fits within our theoretical framework of the ecosocial theory of disease distribution. Additionally, our EWAS and meta-analyses of air pollution showed clear association between two road traffic-related measures of air pollution, and DNAm of multiple CpGs in multiple genes that have been consistently associated with inflammation and infection, suggesting that the environment people live may induce inflammatory changes. Our study has added to the existing literature on air pollution; there are few EWAS studies looking at NOx (*n* = 5), and two so far looking at black carbon, with little consistency between studies. In total, this work highlights the need for researchers to consider multiple levels of discrimination and adversity across the lifecourse, especially structural inequities in the material world in which people live, to fully elucidate drivers and biological mechanisms of inequitable health.

Associations detected at the genome-wide level in MBMS related more closely to early life exposures (being born in a Jim Crow state and low educational attainment); in MESA, they related more to current experiences and exposures (air pollution, racialized economic segregation, and experiences of discrimination), possibly reflecting the relatively older age of the MESA participants. The much stronger associations with air pollution in MESA compared to MBMS could potentially be due to: (1) the use of purified monocytes in MESA, with a single cell type making associations easier to detect; (2) less variation in exposure to air pollution in MBMS compared MESA; (3) longer duration of air pollution exposure in MESA (due to older age of the participants); or (4) reduced statistical power to detect EWAS effects in MBMS, due to lower quantities of DNA [77].

Notably, inflammation was the predominant pathway indicated in the air pollution analyses, both via putative gene functions and enrichment analyses. These findings underscore that while there is a large psychosocial literature on inflammation being a mechanism by which discrimination harms health [32, 78, 79], it is also critical to consider inequities in biophysical exposures in the material world as an important driver of this inflammation. Overall, air pollution sites tend to be enriched for inflammation in MBMS and infection in MESA; this could represent different mechanisms of the same process due to the different blood cell types sampled in the two cohorts; with monocytes being specialized in infection prevention, and neutrophils (the highest proportion cell in whole blood) being specialized in inflammatory responses. However, this enrichment for inflammatory genes may change depending on the tissue measured.

Our study identified a greater number of associations with air pollution measures than previous work in MESA [20, 80]; this is likely due to the fact that we do not adjust for recruitment site (which would reduce variation in the exposure because exposure is location-dependent); and previous analyses have adjusted for racialized group membership, which is also associated with air pollution exposure; this may have masked the effects that we have detected. This joins other research that has demonstrated the importance of considering spatial effects of air pollution [81]. However, further replication efforts are necessary because our results did not overlap DNAm sites that have previously been robustly associated (which we defined as present in two or more studies) with air pollution measures in previous EWAS studies.

A limitation of our study is that we cannot infer causality. Although it would be possible to conduct Mendelian randomization instrumenting *cis*-mQTLs, we did not conduct this analysis because we think the results would be highly speculative. Additionally, the MESA sample we used may have been subject to selection bias, because (1) individuals who had experienced prior cardiovascular events were excluded from recruitment, and (2) a number of participants died between Exam 1 and Exam 5. If adversity and discrimination are associated with these cardiovascular events and mortality, associations could be biased in MESA. Replication of our findings in further studies is needed, because of the large set of exposures we tested, and because it is difficult to tell whether our lack of replication is due to differences between MBMS and MESA (such as sample type), or inconsistency of association between DNAm and the exposures. Our samples sizes were relatively small compared to recent cohorts with epigenetic data, which may have reduced our power to find effects, particularly in MBMS. We would encourage other studies to collect data on multiple levels of discrimination, including historical exposures, so this work can be extended. Finally, we did not replicate the findings of a recent EWAS of racial discrimination conducted in MESA [29], one of the cohorts we tested. Possible reasons for this are our differing sets of EWAS covariates, and a slight difference in construction of the measure of racial discrimination (theirs comparing two or fewer experiences to more than two, and ours comparing 1–2 with both zero and three or more).

## Conclusions

We think this work provides direction for future epigenetic studies to consider the role of inequitable adverse social and biophysical exposures across the lifecourse, including but not limited to structural discrimination. We also think it demonstrates the value of grounding EWAS study design in theory. Our results suggest inflammation may be a key biological pathway by which inequities become embodied, in our case driven primarily by exposure to air pollution, and not self-reported racial discrimination. These findings accordingly suggest that attention to how social inequities shape biophysical as well as social exposures is crucial for understanding how societal inequities can become embodied, via pathways involving DNAm.

## Supplementary Information

Additional file1 (CSV 28 KB)

Additional file2 (XLSX 21 KB)

Additional file3 (CSV 20 KB)

Additional file4 (PDF 18399 KB)

Additional file5 (DOCX 195 KB)

## Data Availability

This study (NIH Grant number R01MD014304) relied on three sources of data, each of which is subject to distinct data sharing stipulations: (1) the non-public data from the “My Body, My Story” (MBMS) study; (2) the non-public data from the Multi-Ethnic Study of Atherosclerosis (MESA; data use agreement G638); and (3) the public de-identified data from the US Census, the American Community Survey, and the State Policy Liberalism Index. We provide descriptions of these data sharing stipulations and access to these data below; this information is also available at: https://www.hsph.harvard.edu/nancy-krieger/data-sharing-resources/ •ICE metrics relating to racial composition, income distribution, and housing tenure that were derived from sources in the public domain, i.e. the US Census and the American Community Survey are available at the census tract level now on GitHub. •Code used to construct the variables is available on GitHub and here. •The State Policy Liberalism Index data used in our study is also publicly available and can be obtained from the Harvard Dataverse. Reference: Caughey, Devin; Warshaw, Christopher, 2014, “The Dynamics of State Policy Liberalism, 1936–2014”, http://dx.doi.org/10.7910/DVN/ZXZMJB Dataverse [Distributor] V1 [Version]. •De-identified data from the My Body My Story study used for this project will be made available only for purposes approved by the study PI, as stipulated by the study’s informed consent protocol. The application form to obtain these data will be made available via this website after completion of this project in late Fall 2024. •Data from the Multi-Ethnic Study of Atherosclerosis (MESA) must be obtained directly from the MESA website via their application protocol. •The scripts to run the EWAS and downstream analyses are available on GitHub. •EWAS summary statistics will be uploaded to the EWAS catalog website upon publication.
