# Supplementary material for: An epigenome-wide analysis of DNA methylation, racialized and economic inequities, and air pollution
Source: Clin Epigenetics. 2025 Nov 27;18:4. doi: 10.1186/s13148-025-01929-6 (PMC12764144; doi:10.1186/s13148-025-01929-6)
Supplement: Supplementary file 4 — Additional file4 (PDF 18399 KB) [file 13148_2025_1929_MOESM4_ESM.pdf]

Supplementary figures

Miami plots

MBMS full cohort Miami plots

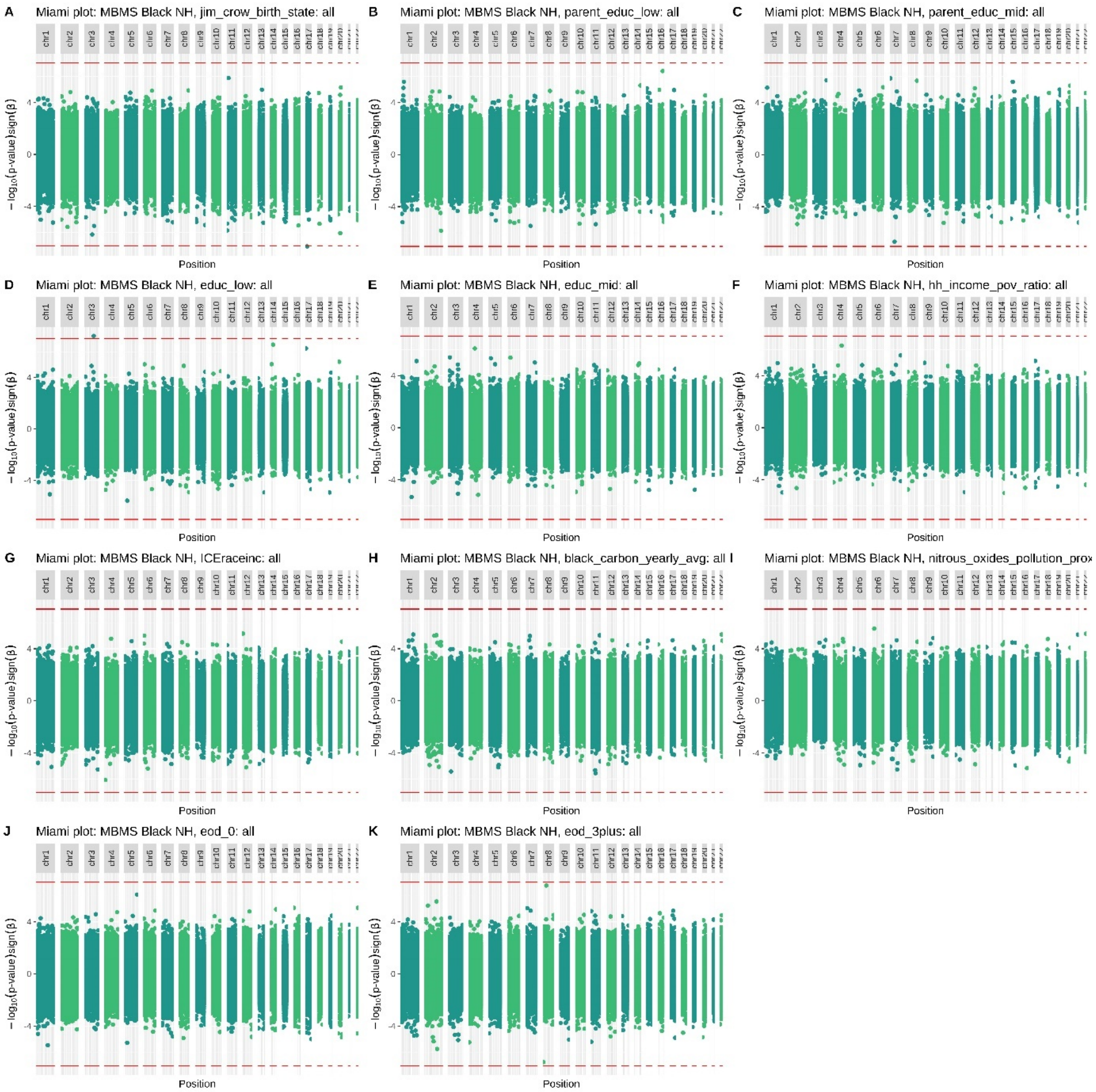

Figure 1: MBMS Black NH Miami plots

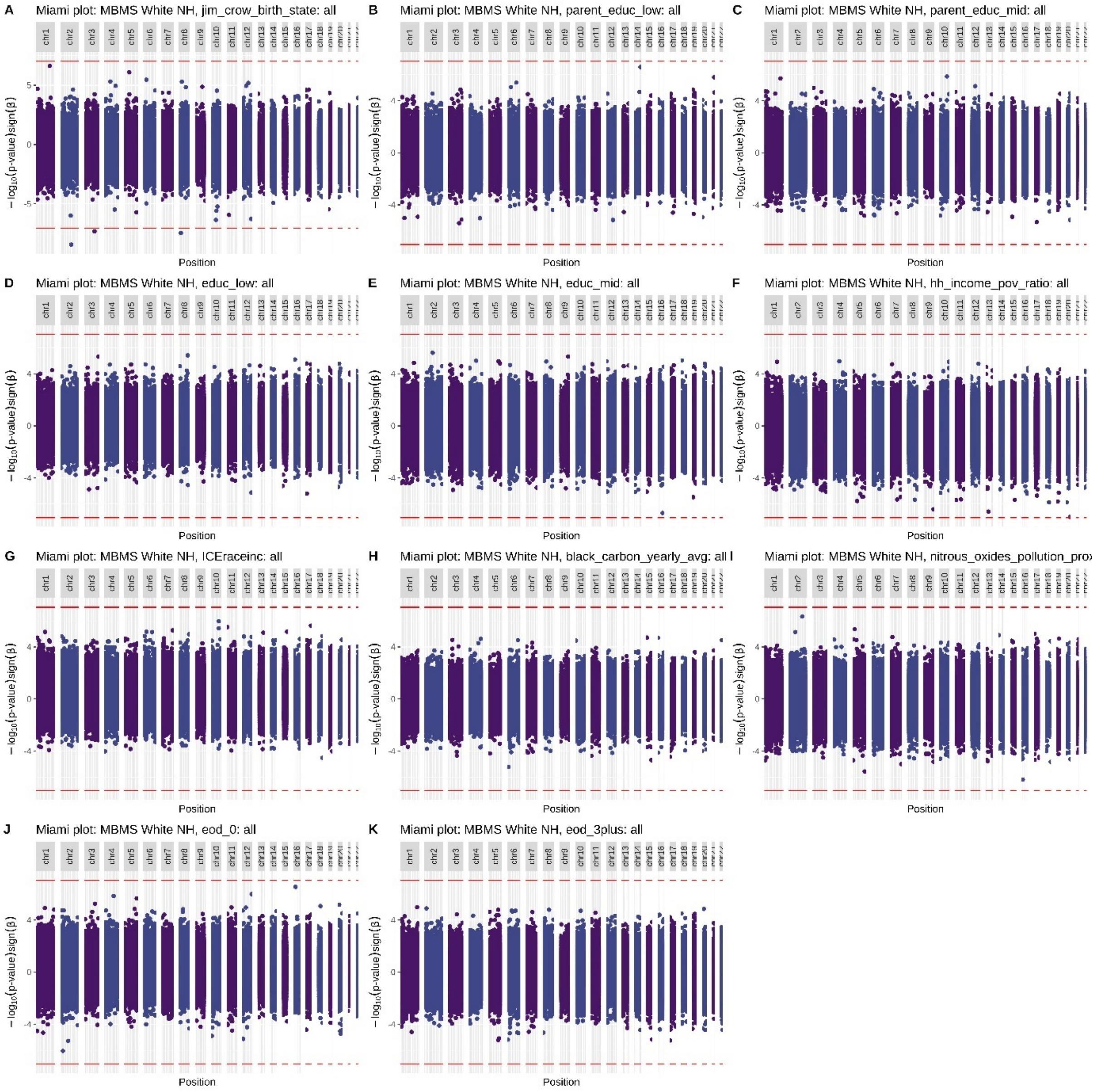

Figure 2: MBMS white NH Miami plots

MESA full cohort Miami plots

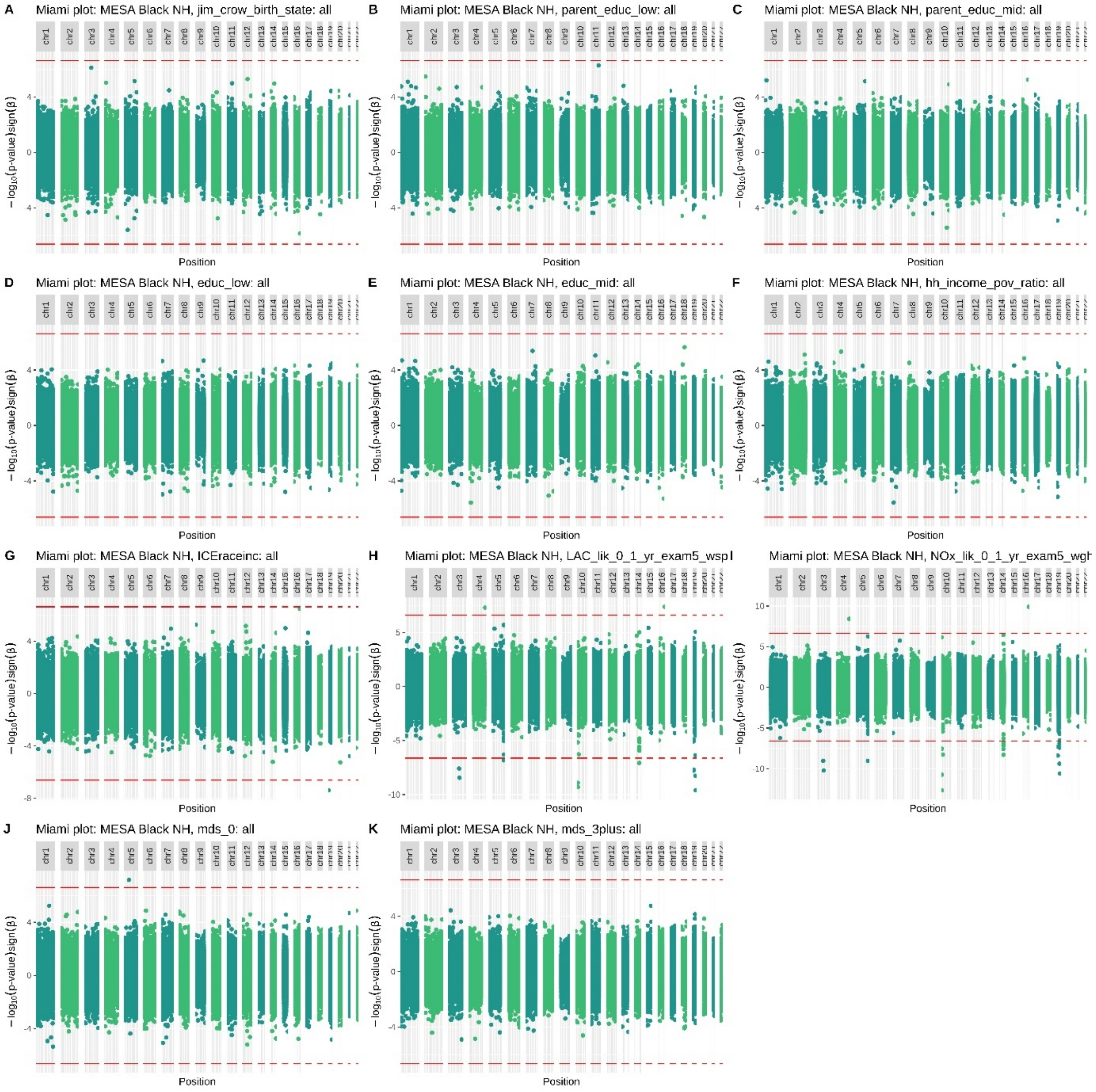

Figure 3: MESA Black NH Miami plots

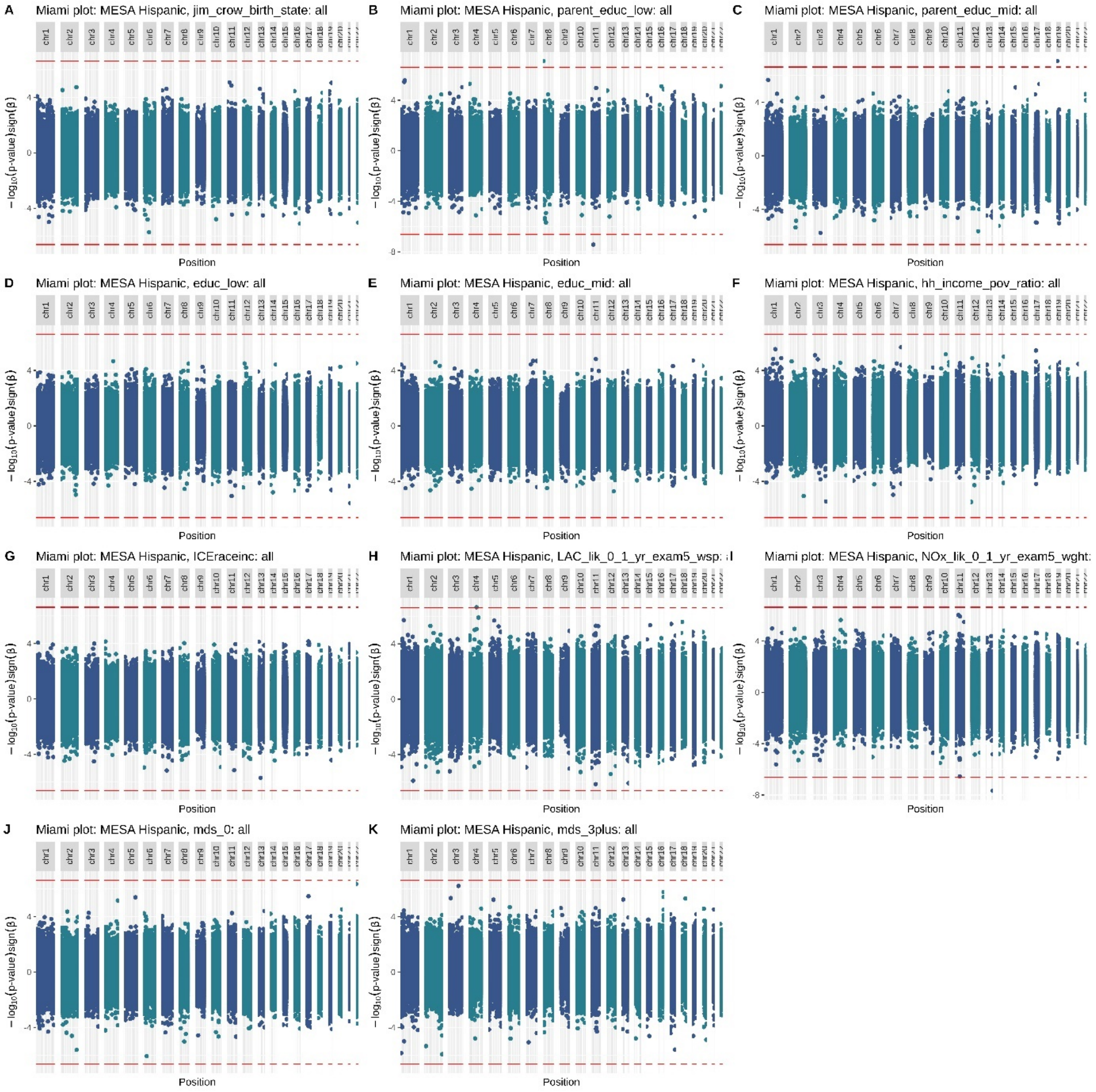

Figure 4: MESA Hispanic Miami plots

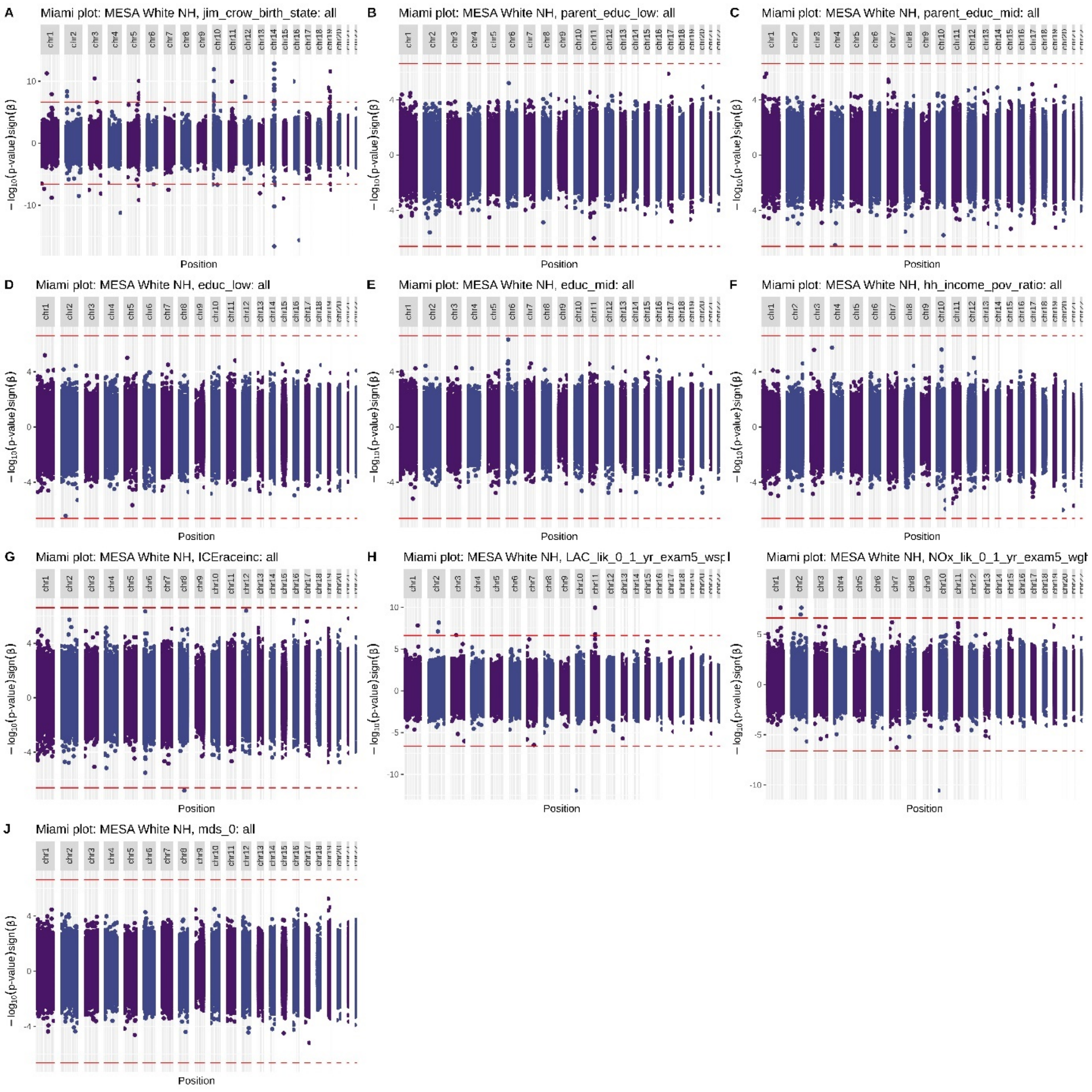

Figure 5: MESA white NH Miami plots

MESA New York and Baltimore subset Miami plots

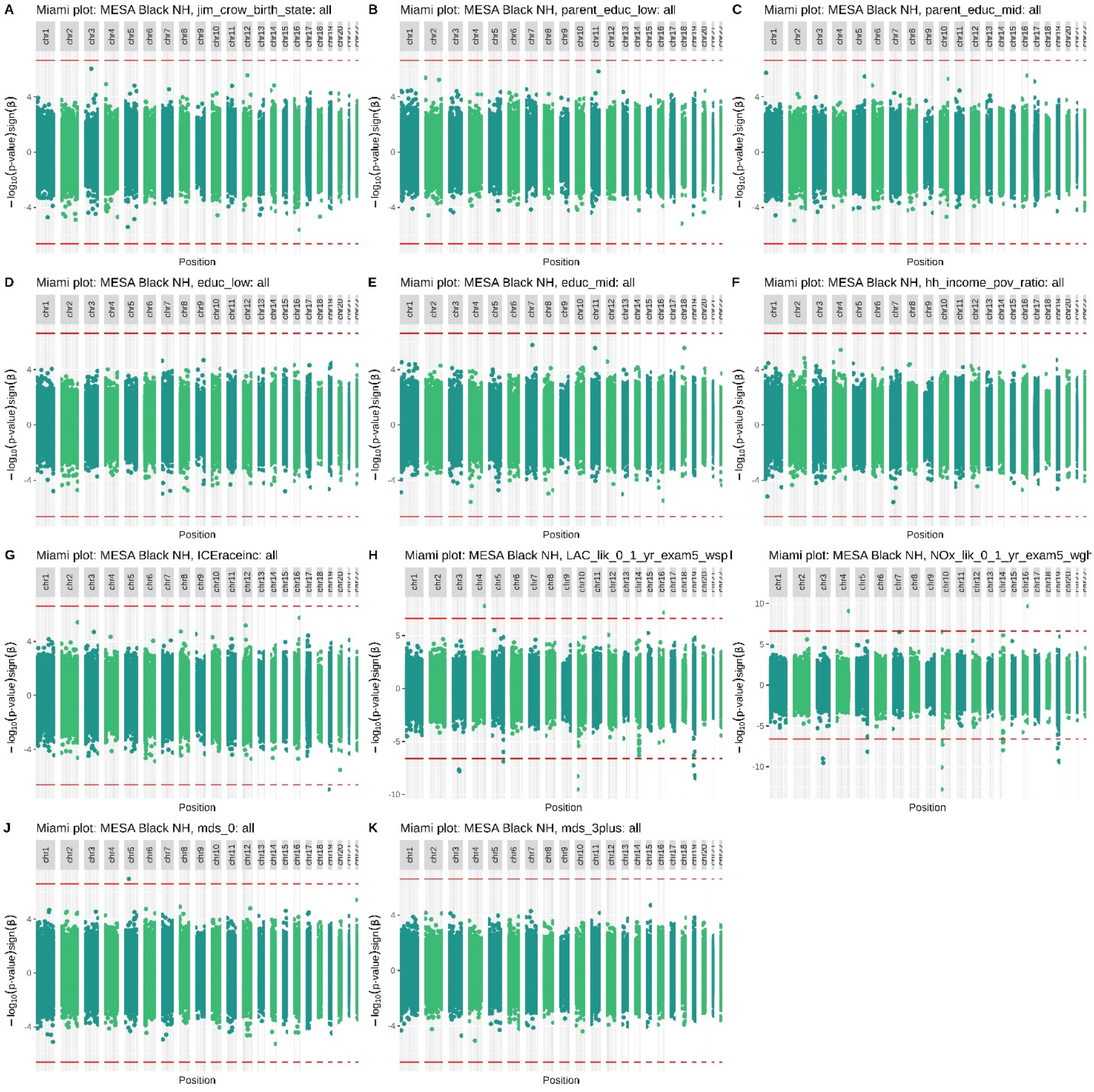

Figure 6: MESA Black NH Miami plots (JHU and COL subgroup)

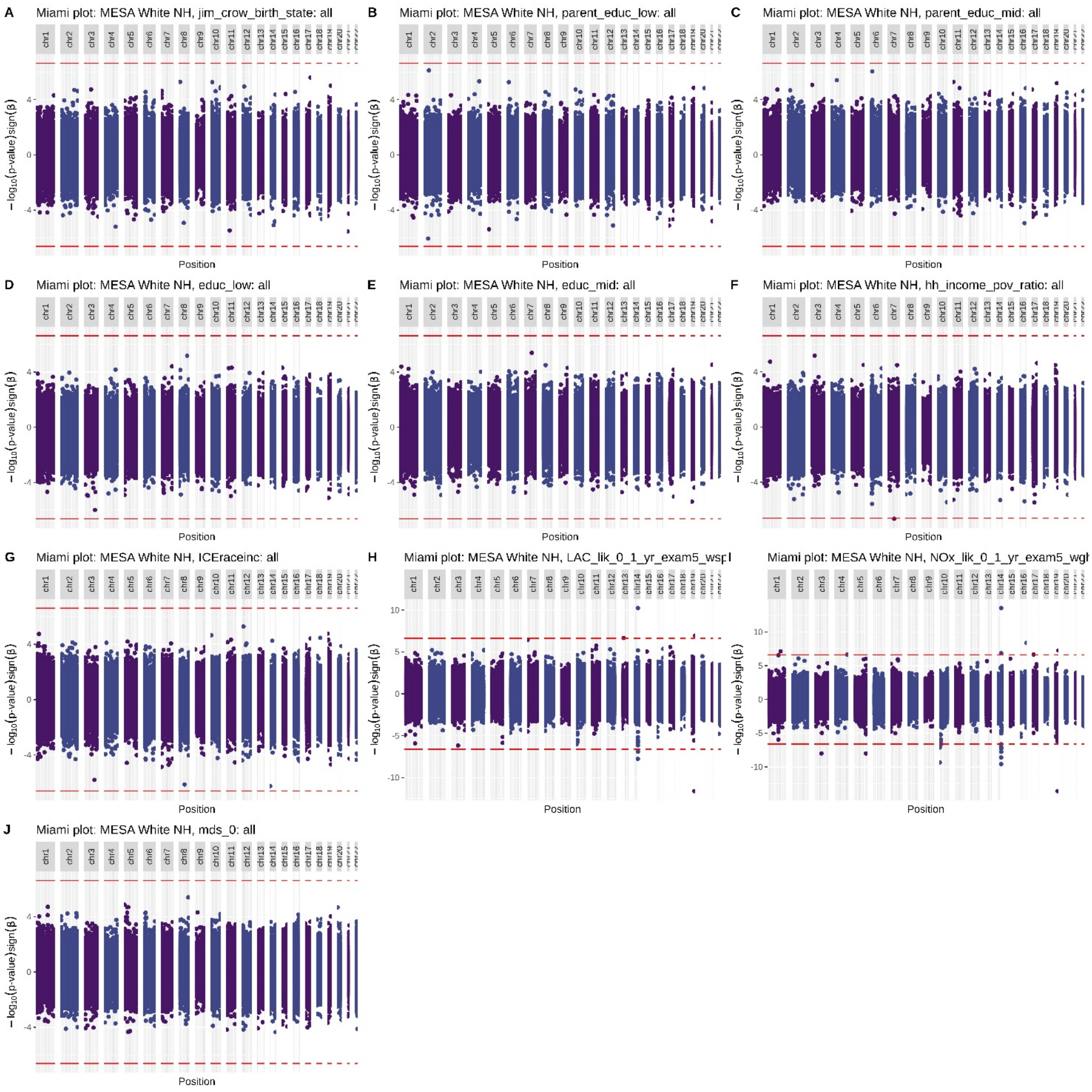

Figure 7: MESA white NH Miami plots (JHU and COL subgroup)

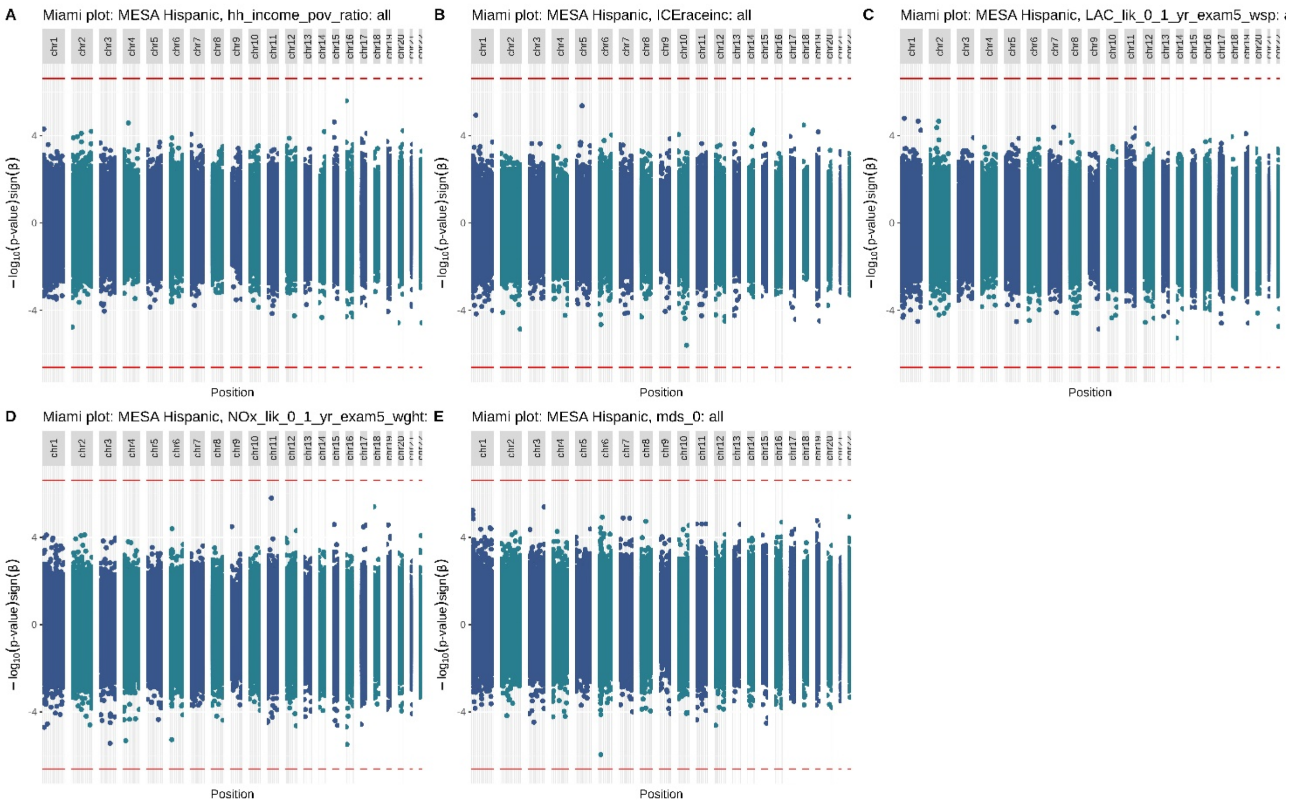

Figure 8: MESA Hispanic Miami plots (JHU and COL subgroup)

QQ plots

MBMS full cohort

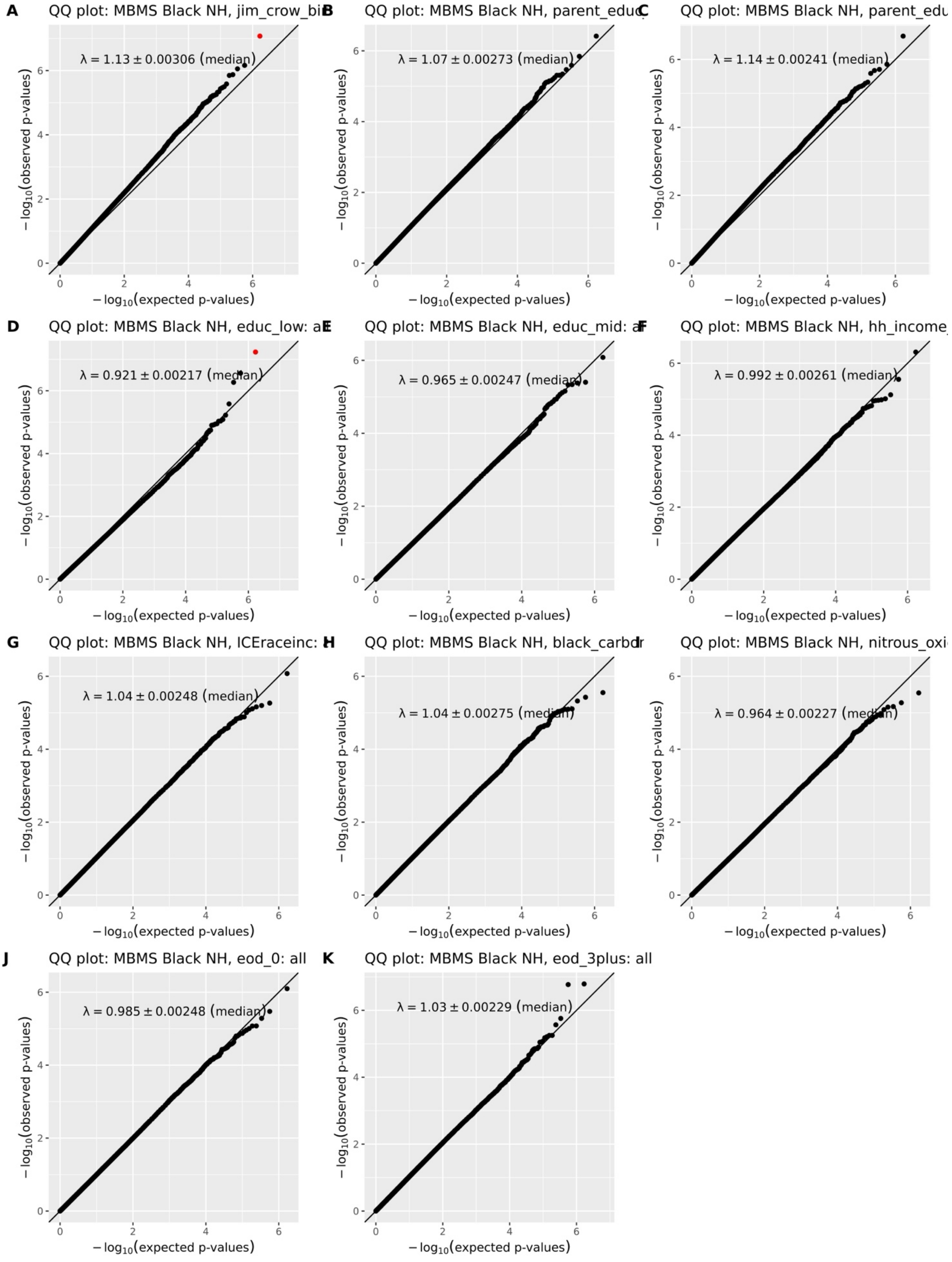

Figure 9: MBMS Black NH QQ plots

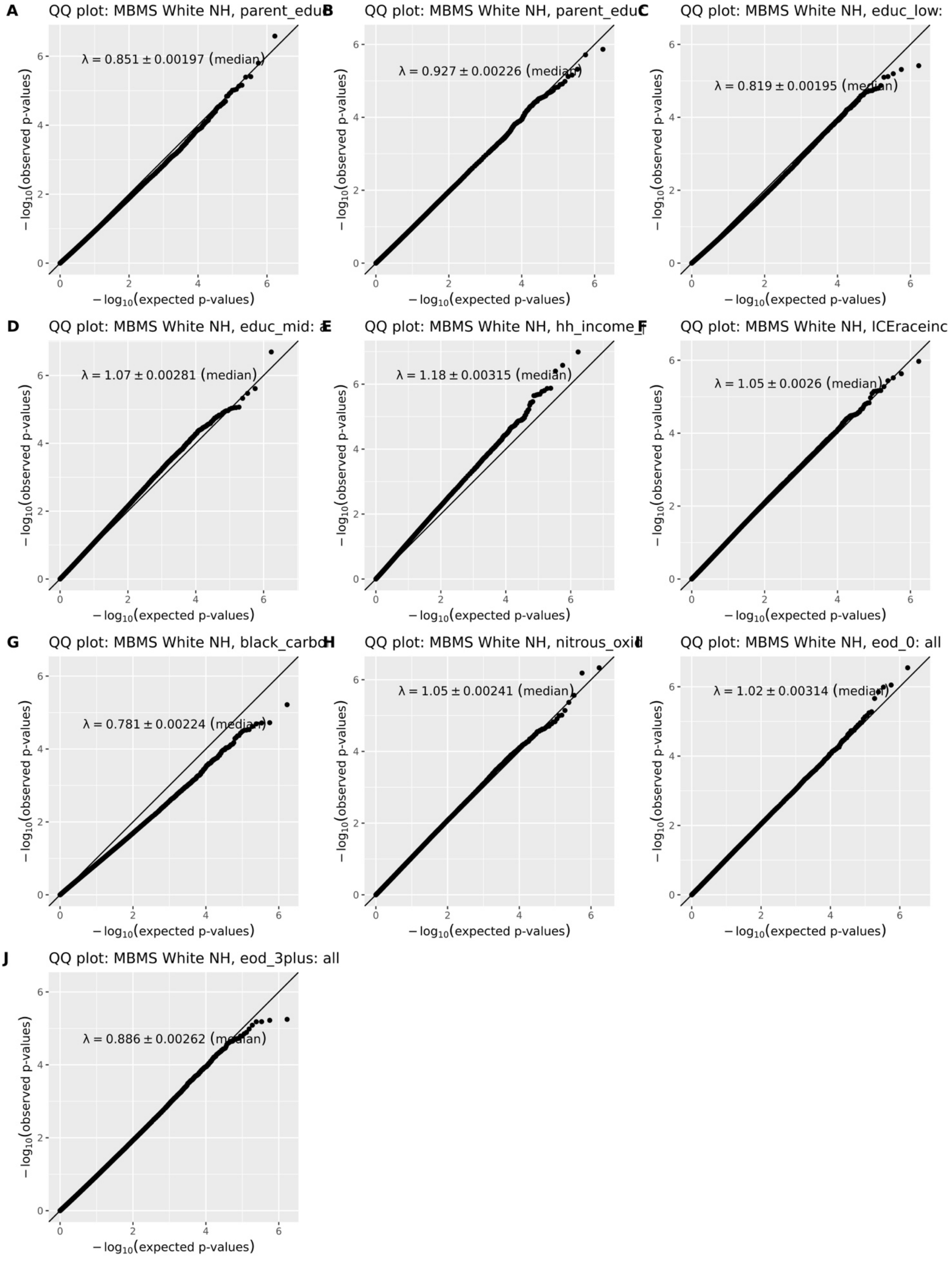

Figure 10: MBMS White NH QQ plots

MESA full cohort QQ plots

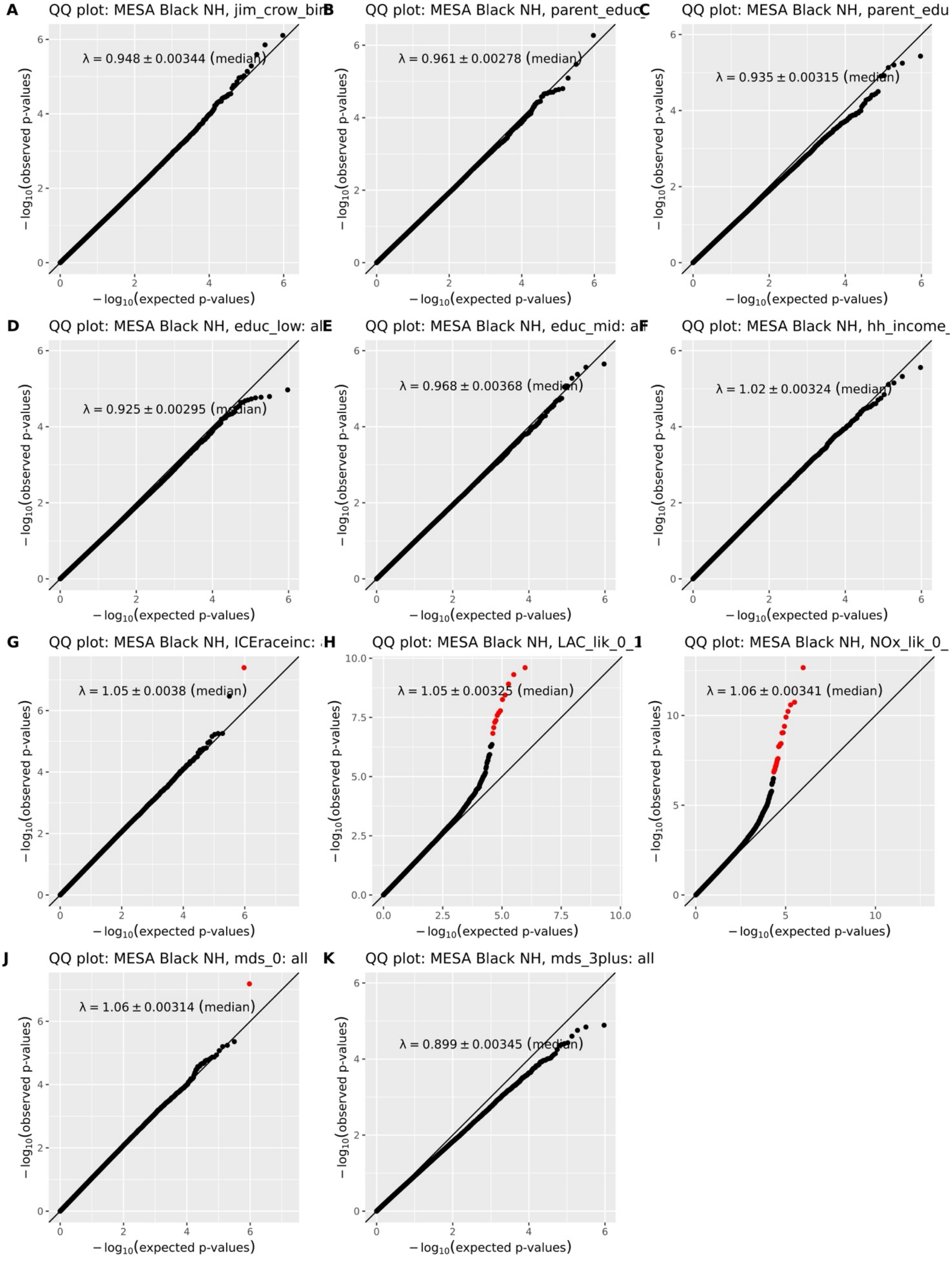

Figure 11: MESA Black NH QQ plots

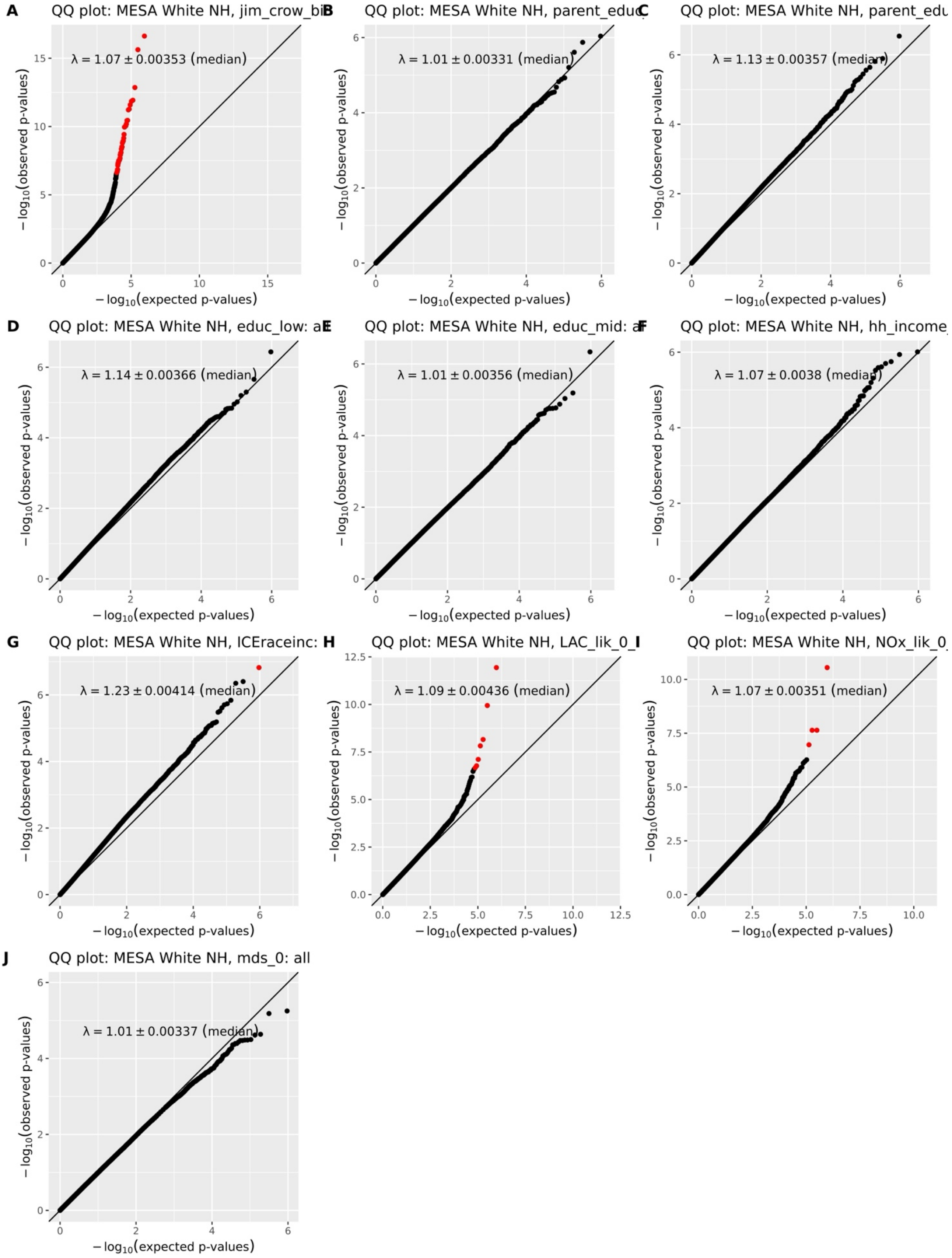

Figure 12: MESA White NH QQ plots

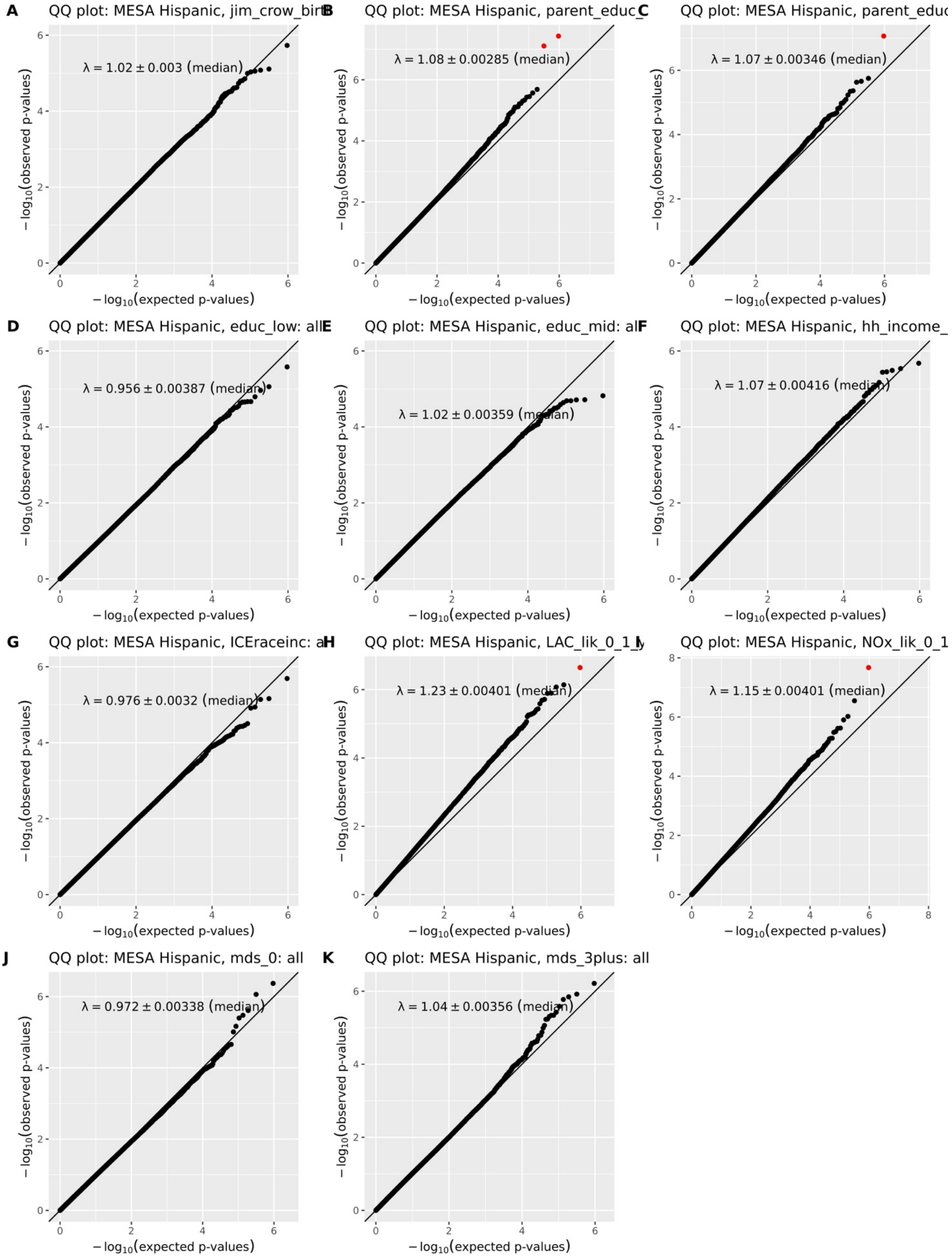

Figure 13: MESA Hispanic QQ plots

MESA New York and Baltimore subset QQ plots

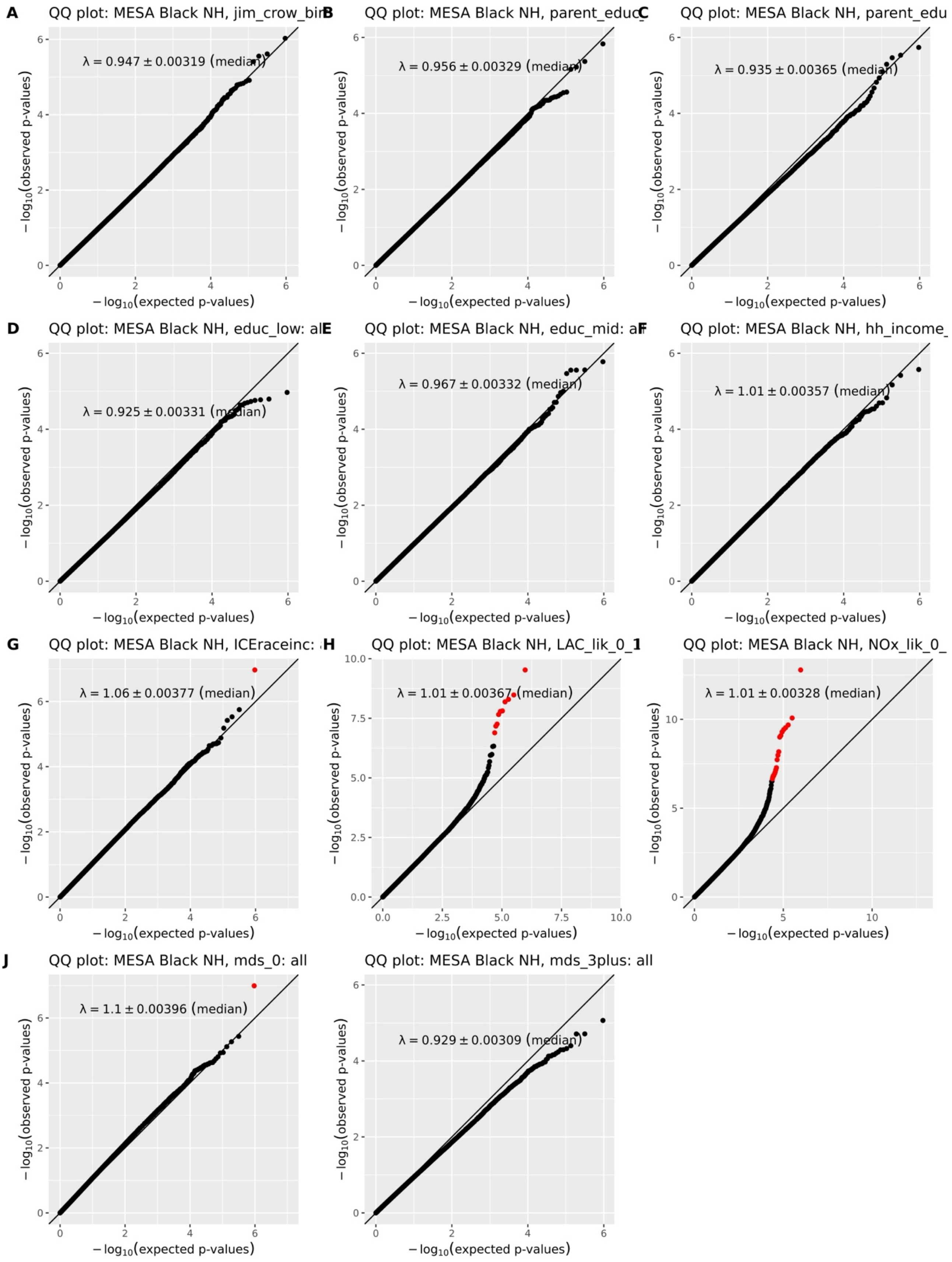

Figure 14: MESA Black NH QQ plots (JHU and COL subgroup)

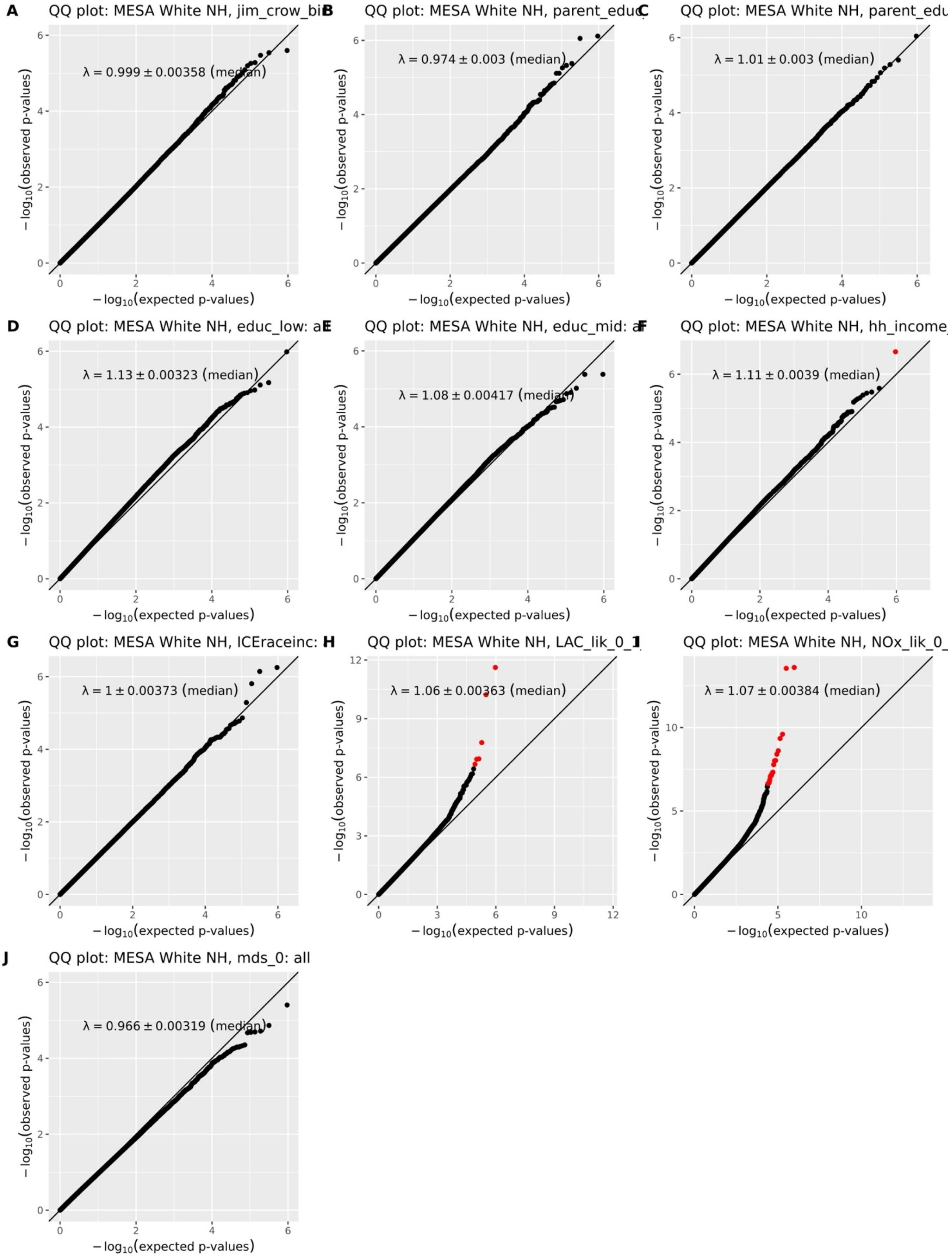

Figure 15: MESA White NH QQ plots (JHU and COL subgroup)

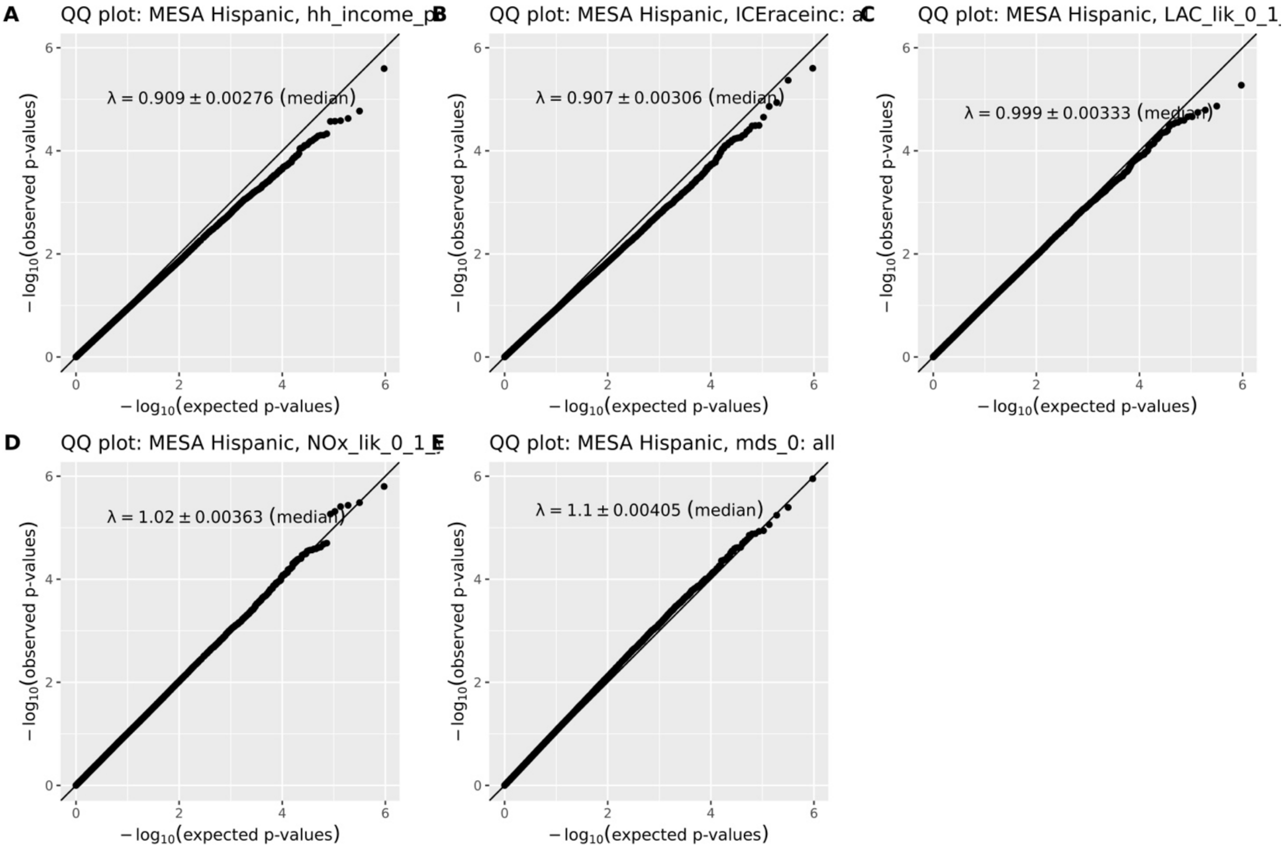

Figure 16: MESA Hispanic QQ plots (JHU and COL subgroup)

EWAS catalog enrichment plots

MBMS full cohort

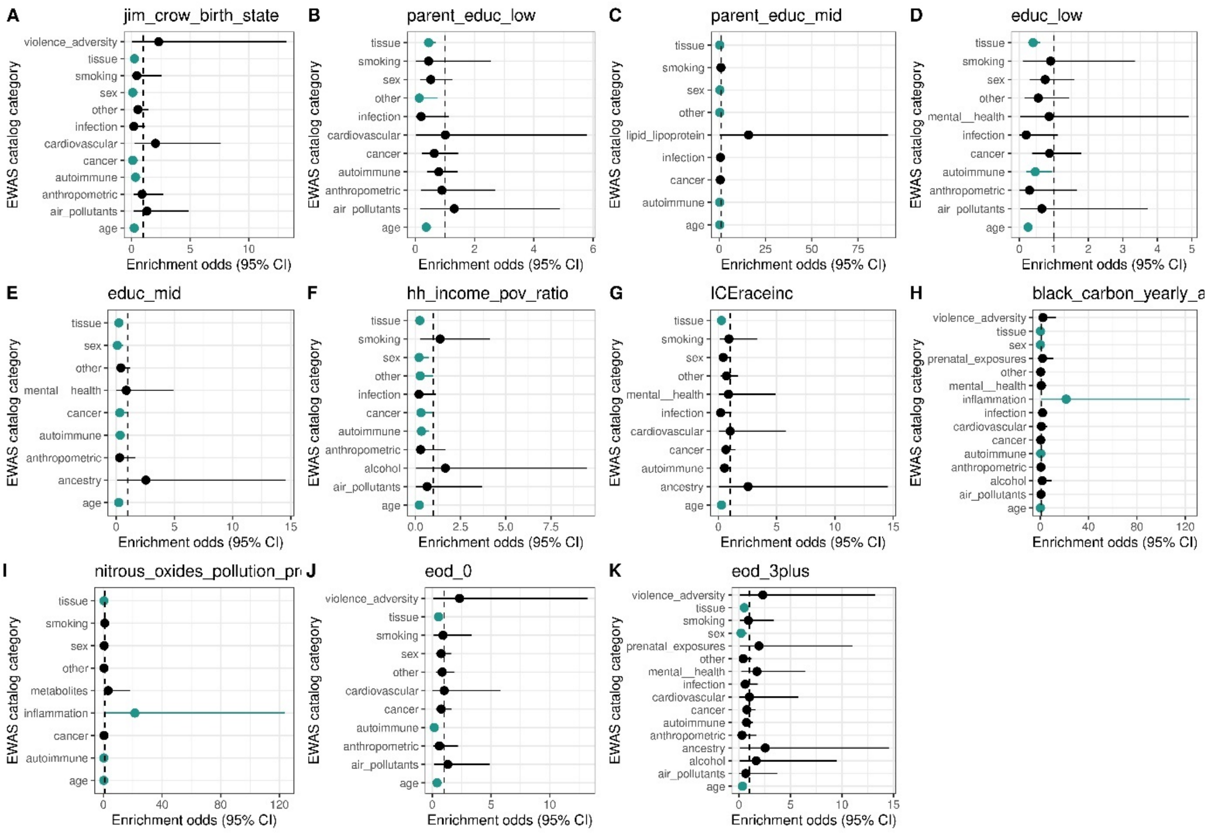

Figure 17: EWAS catalog enrichment plot: MBMS Black NH

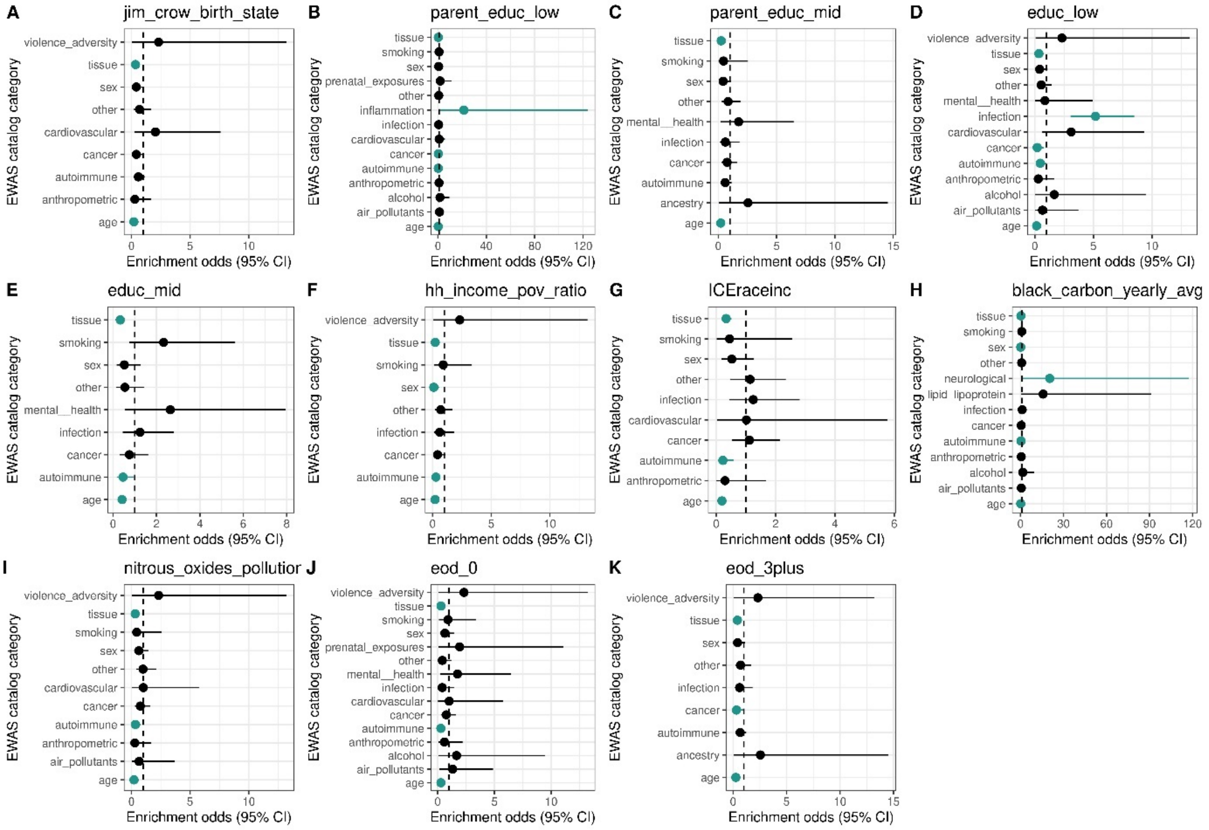

Figure 18: EWAS catalog enrichment plot: MBMS white NH

MESA full cohort

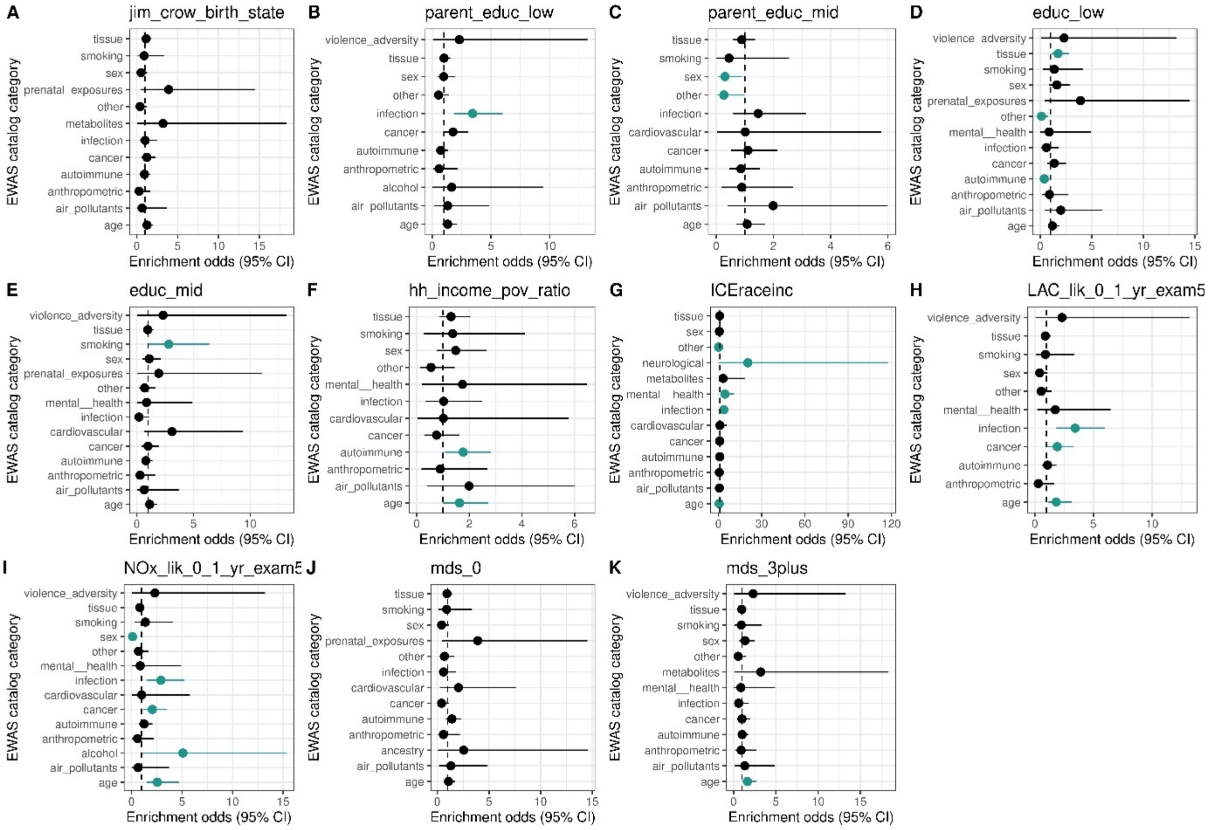

Figure 19: EWAS catalog enrichment plot: MESA Black NH

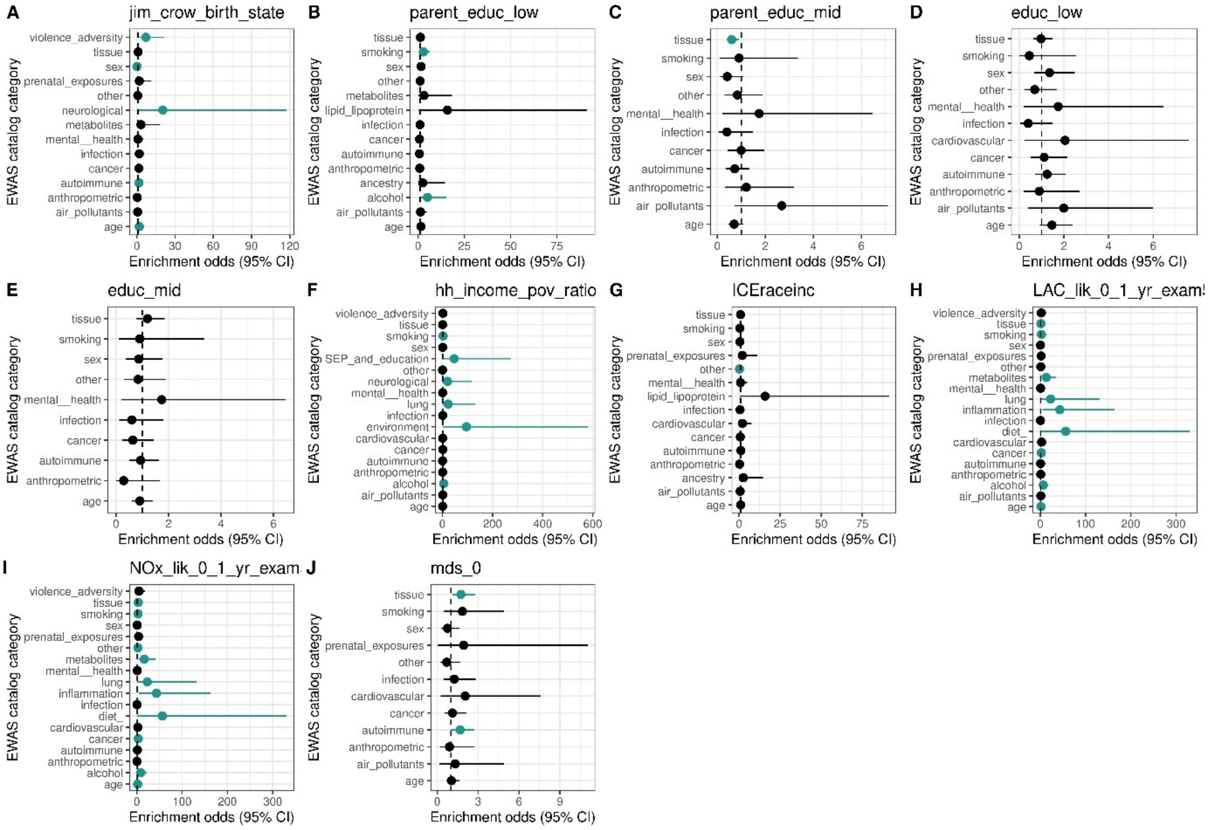

Figure 20: EWAS catalog enrichment plot: MESA white NH

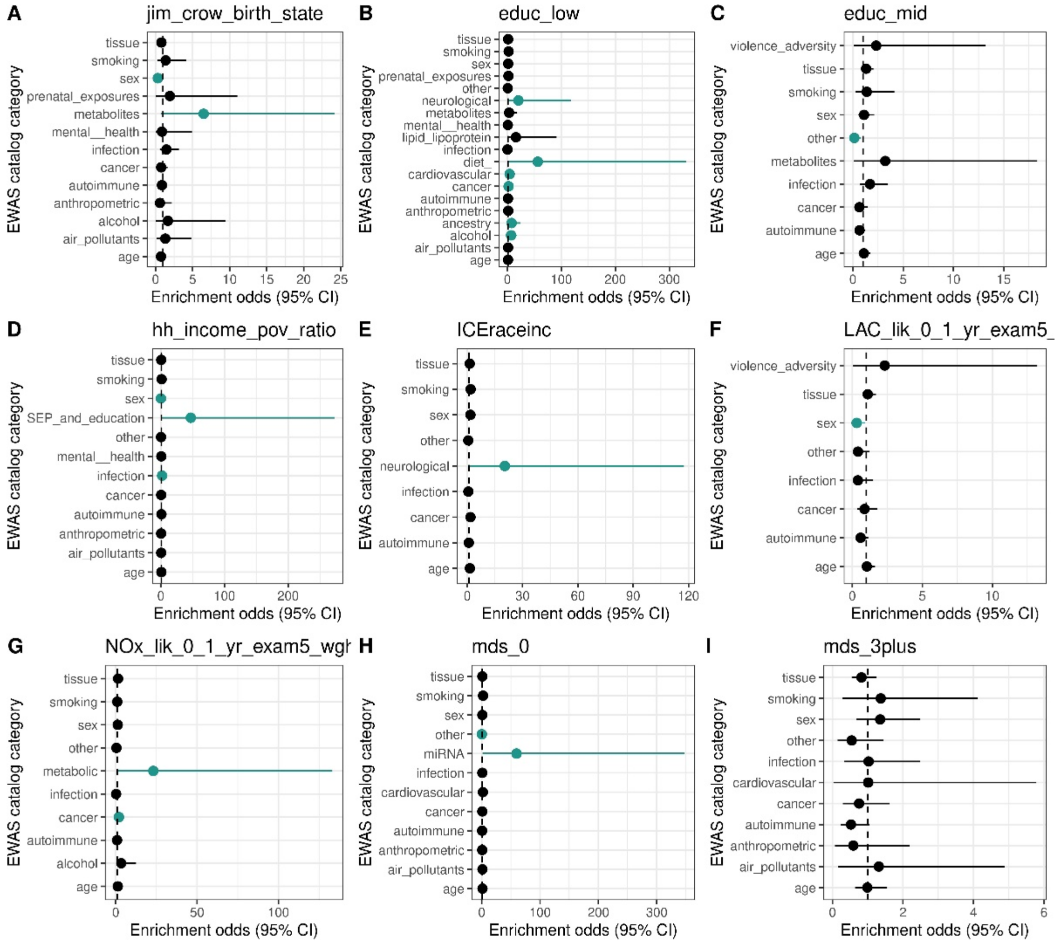

Figure 21: EWAS catalog enrichment plot: MESA Hispanic

MESA JHU/COL subgroup

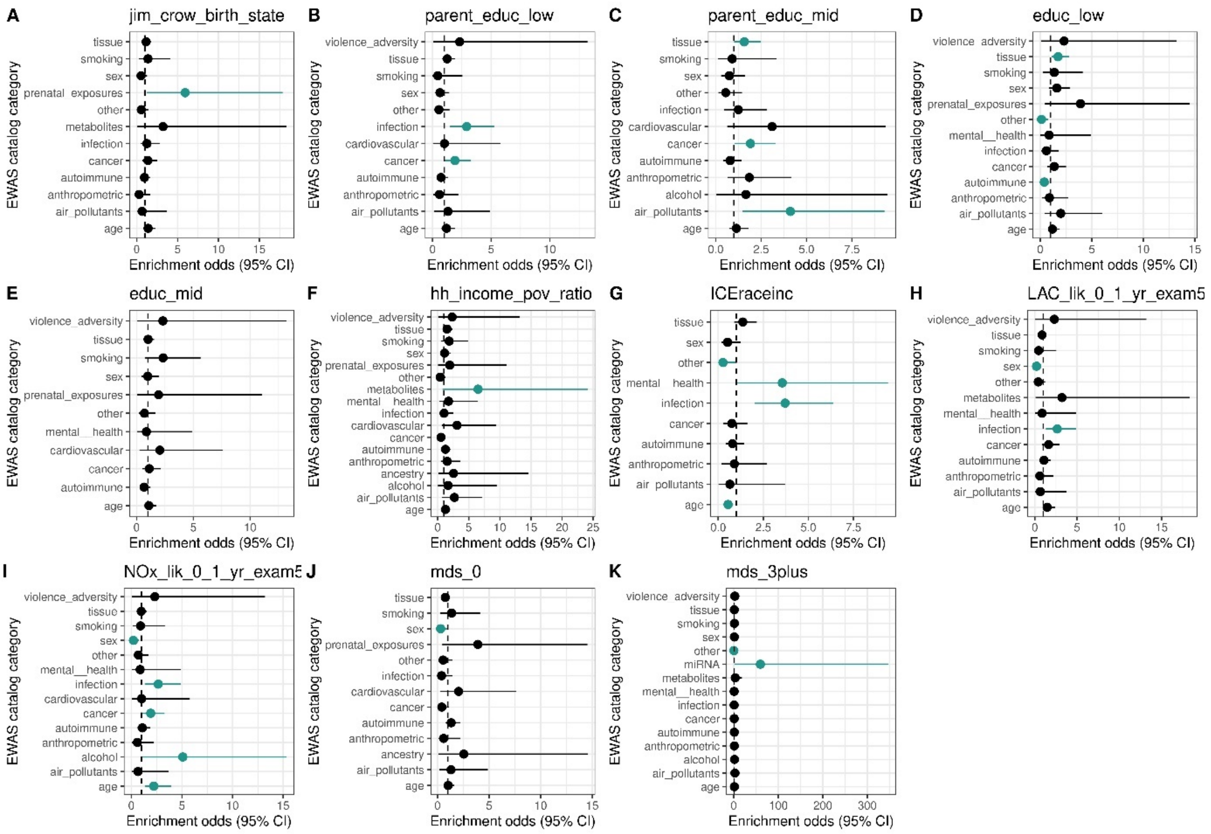

Figure 22: EWAS catalog enrichment plot: MESA Black NH JHU + COL subgroup

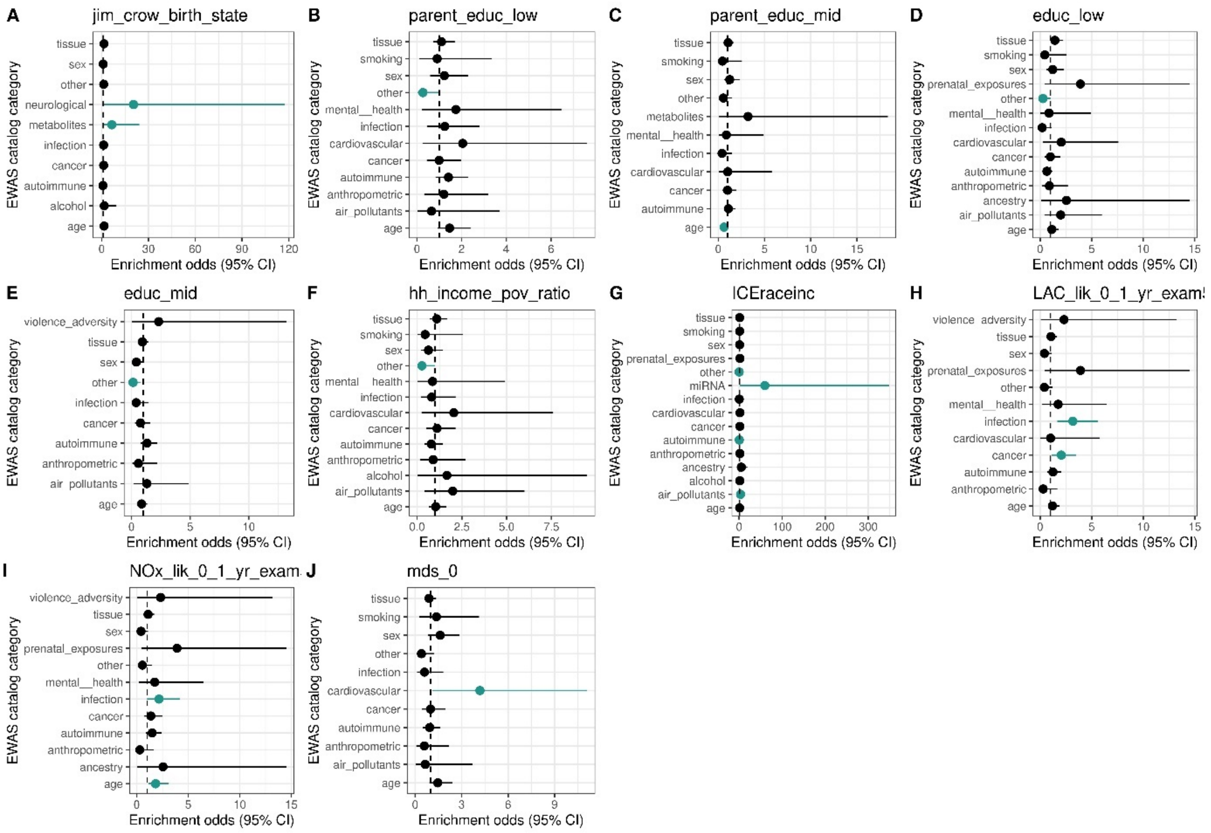

Figure 23: EWAS catalog enrichment plot: MESA white NH JHU + COL subgroup

---

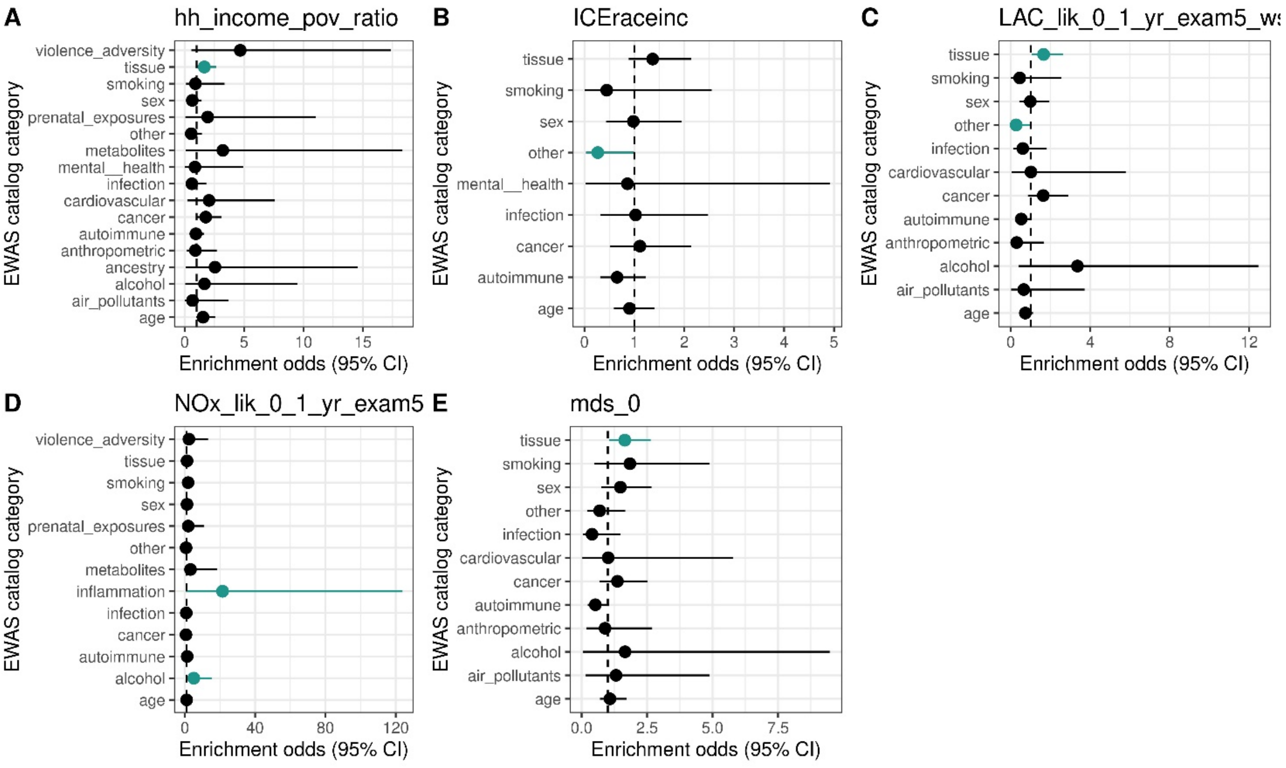

Figure 24: EWAS catalog enrichment plot: MESA Hispanic JHU + COL subgroup

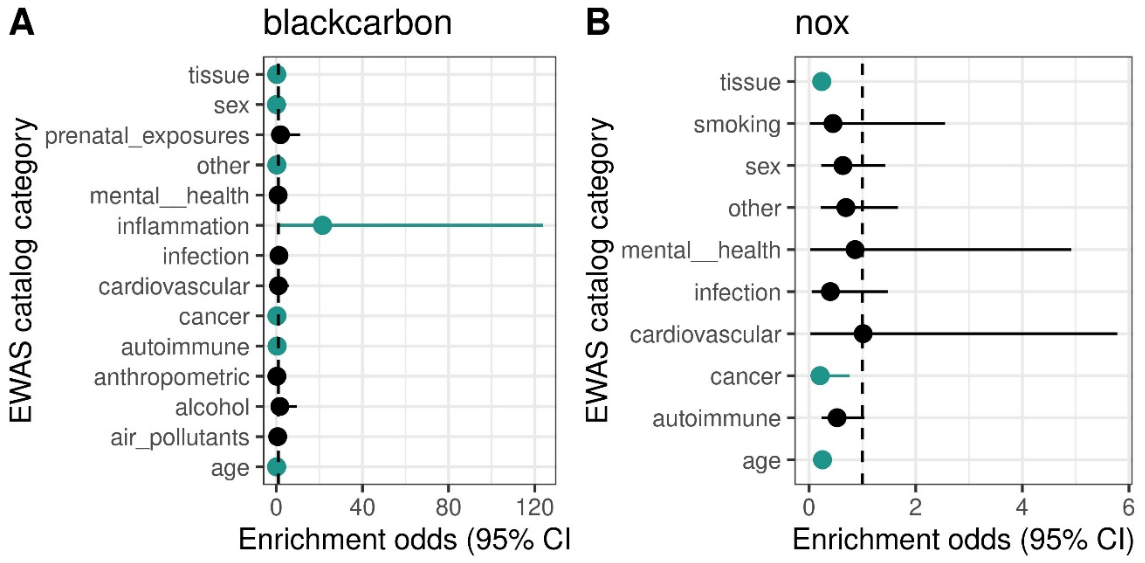

Figure 25: EWAS catalog enrichment plot: MBMS meta-analysis

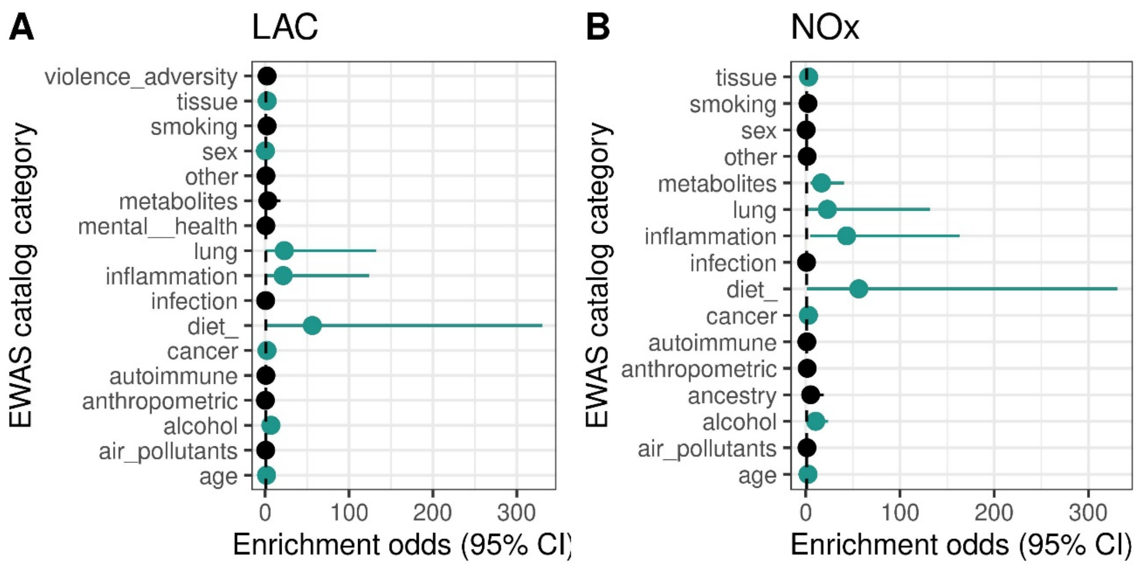

Figure 26: EWAS catalog enrichment plot: MESA meta-analysis

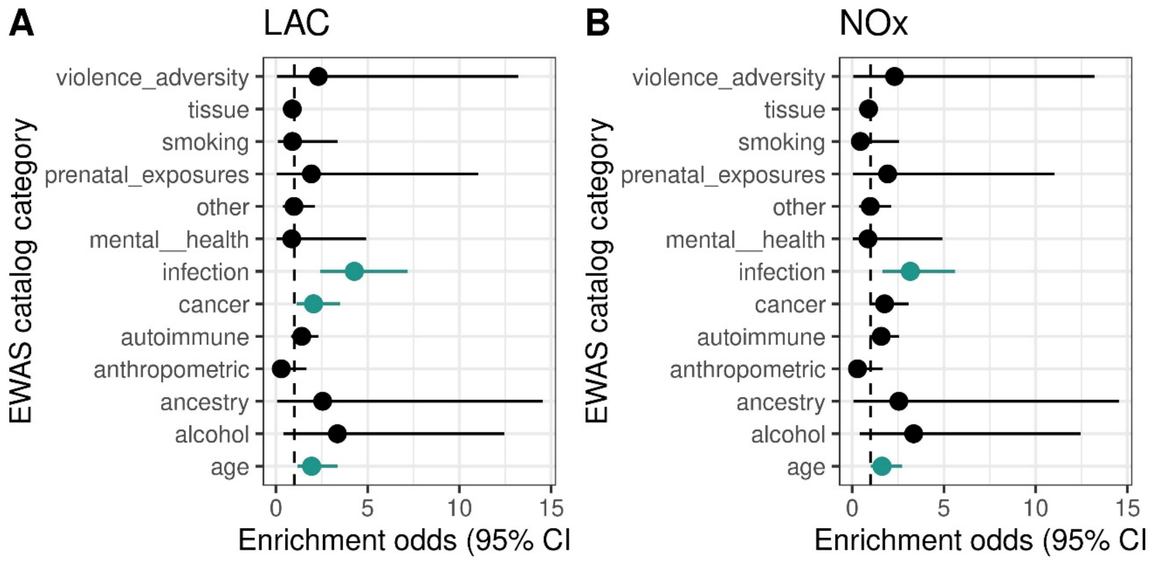

Figure 27: EWAS catalog enrichment plot: MESA meta-analysis (JHU and COL subgroup)

Enrichment for genomic features

MBMS Black NH participants

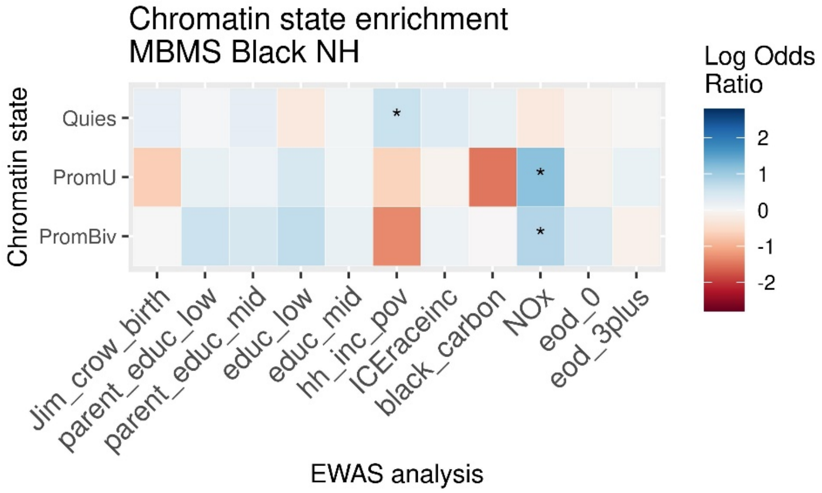

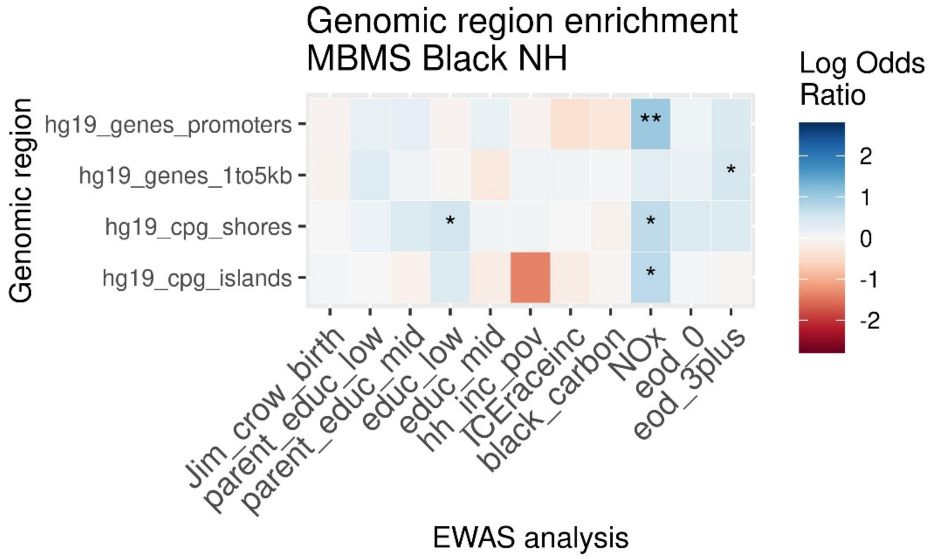

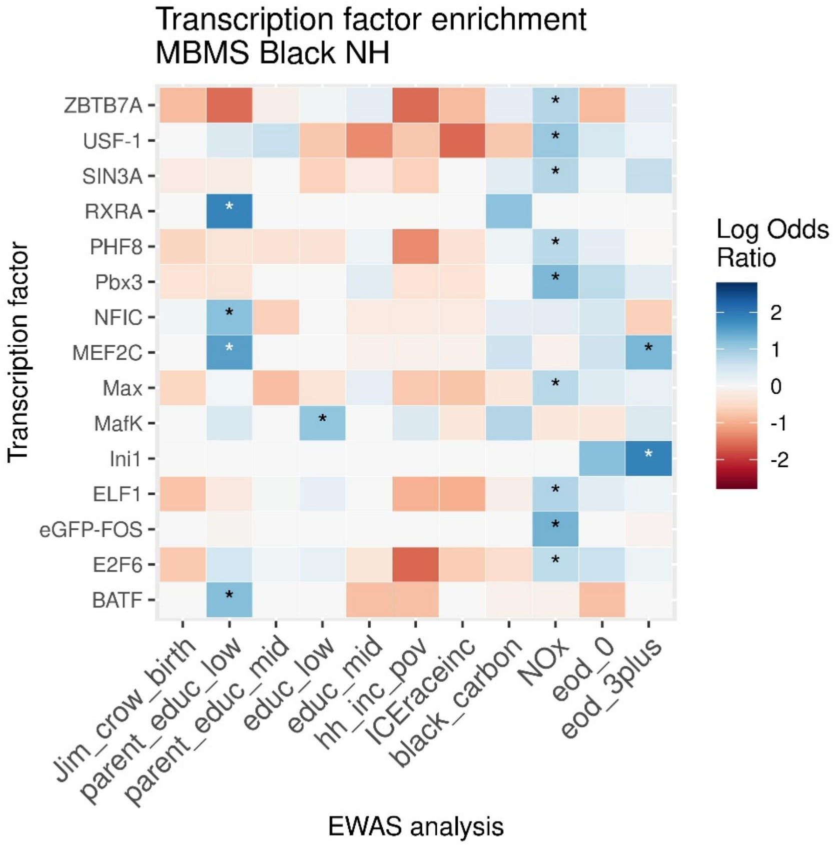

MBMS white NH participants

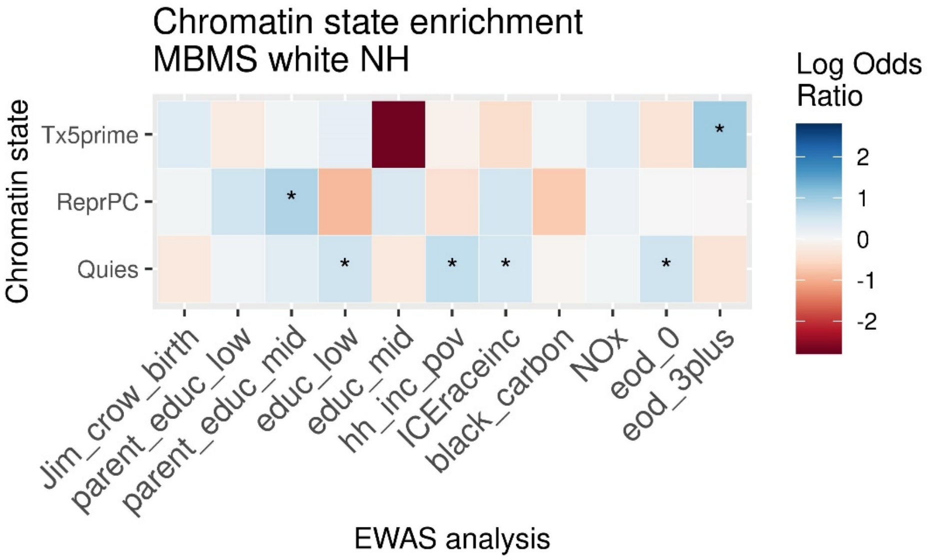

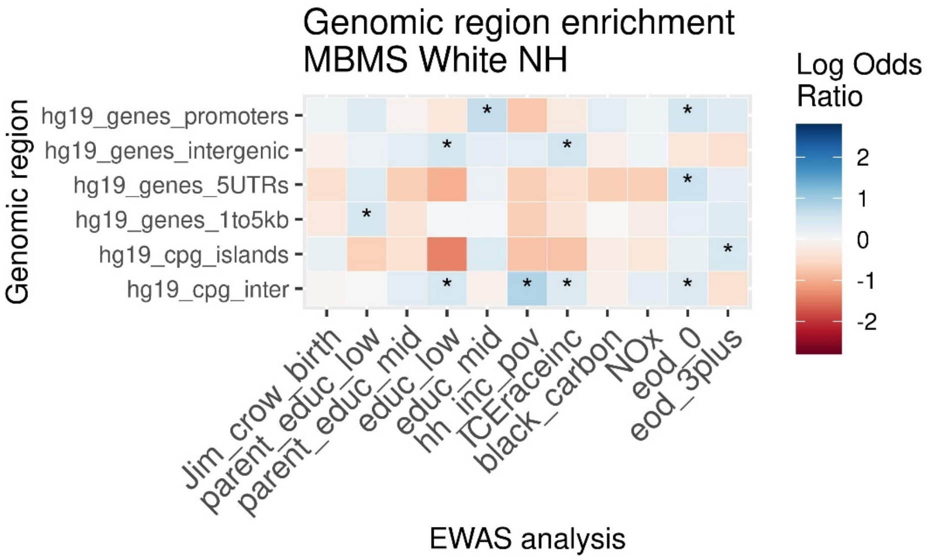
Genomic region enrichment
MBMS White NH
Log Odds Ratio
2
1
0
-1
-2
Genomic region
hg19_genes_promoters
hg19_genes_intergenic
hg19_genes_5UTRs
hg19_genes_1to5kb
hg19_cpg_islands
hg19_cpg_inter
Jim_crow_birth
parent_educ_low
parent_educ_mid
educ_low
educ_mid
hh_inc_pov
ICEraceinc
black_carbon
NOx
eod_0
eod_3plus
EWAS analysis

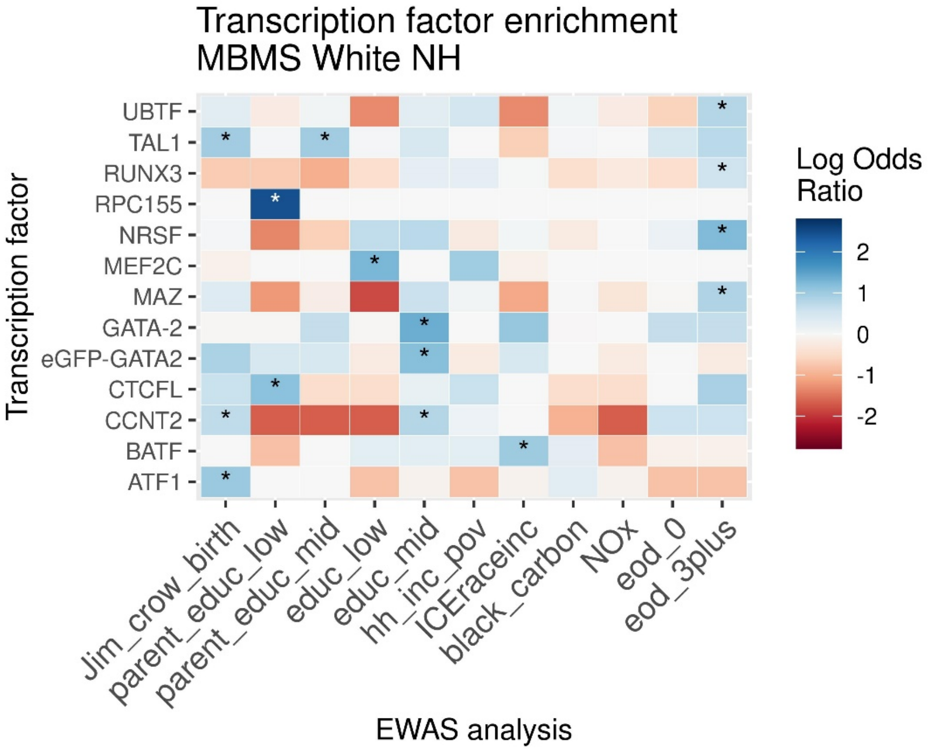
Transcription factor enrichment
MBMS White NH
Log Odds Ratio
2
1
0
-1
-2
Transcription factor
UBTF
TAL1
RUNX3
RPC155
NRSF
MEF2C
MAZ
GATA-2
eGFP-GATA2
CTCFL
CCNT2
BATF
ATF1
Jim_crow_birth
parent_educ_low
parent_educ_mid
educ_low
educ_mid
hh_inc_pov
ICEraceinc
black_carbon
NOx
eod_0
eod_3plus
EWAS analysis

MESA Black NH participants

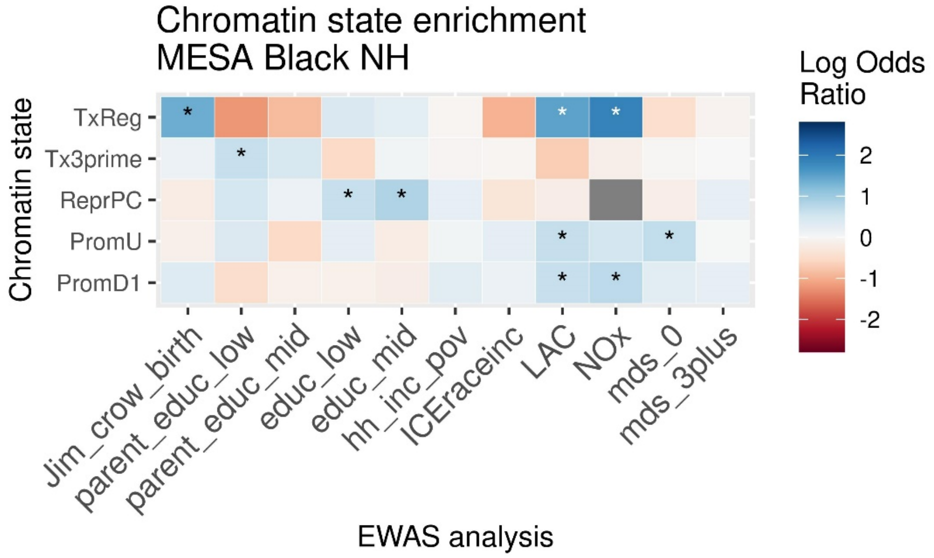

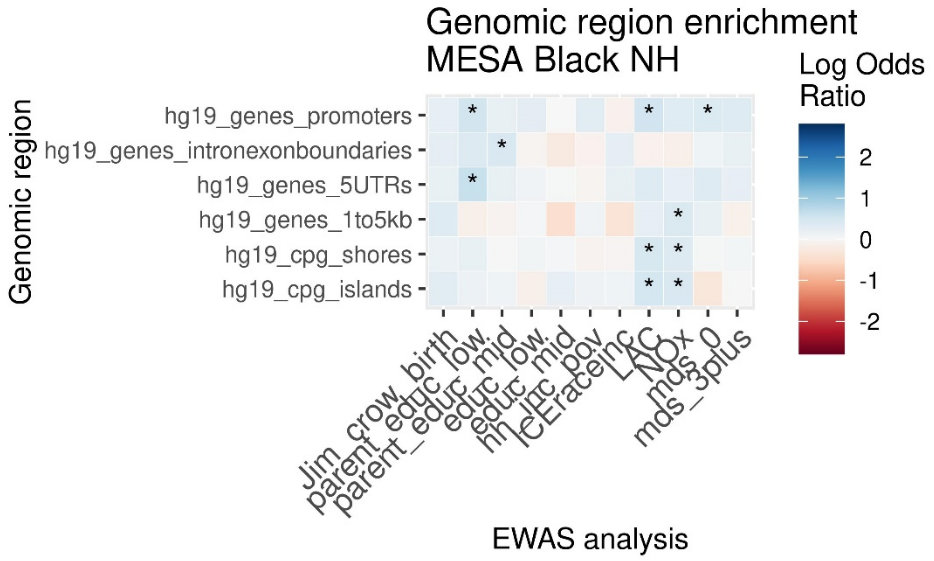

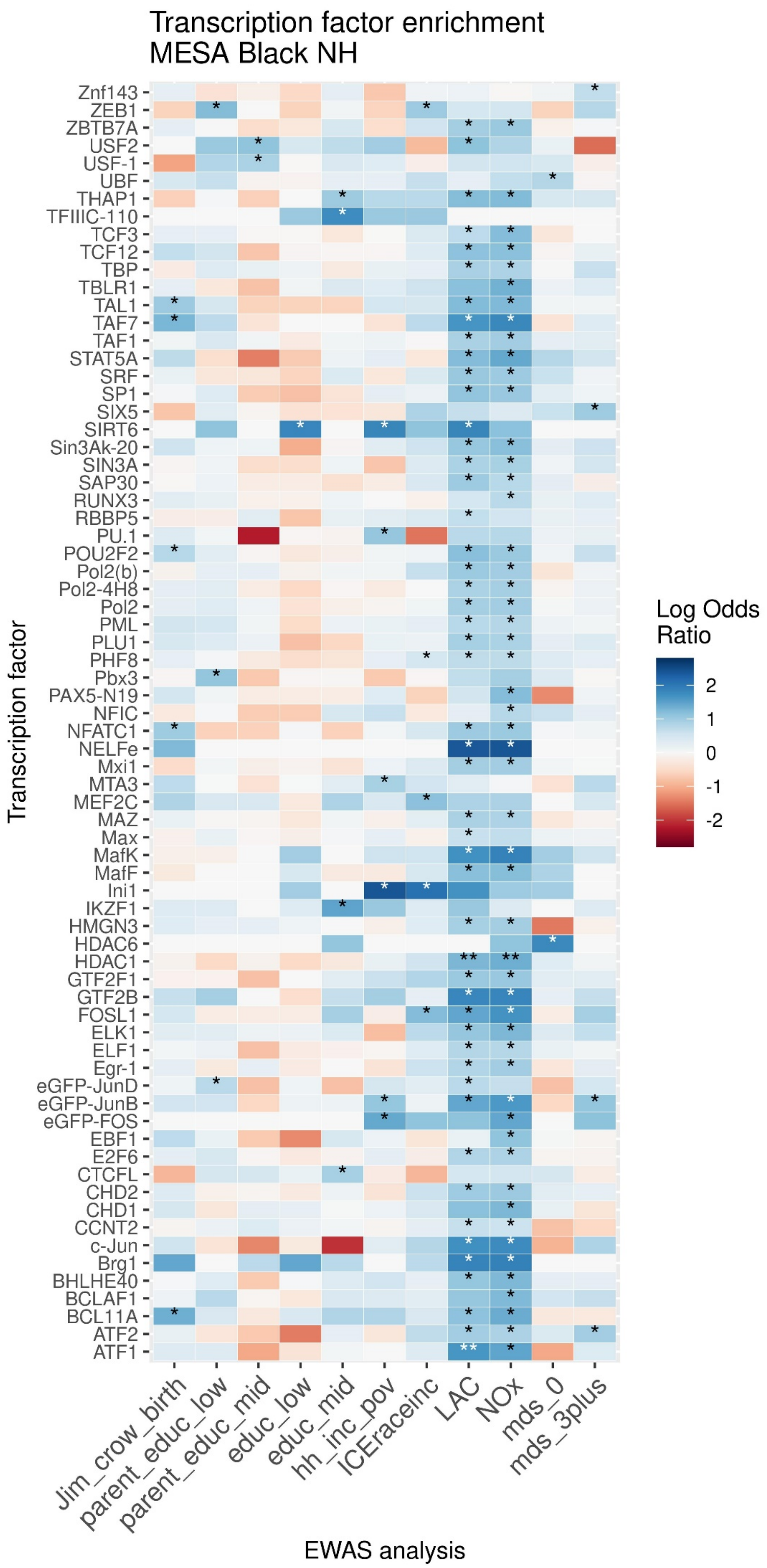

Transcription factor enrichment
MESA Black NH
Transcription factor
Znf143
ZEB1
ZBTB7A
USF2
USF-1
UBF
THAP1
TFIIIC-110
TCF3
TCF12
TBP
TBLR1
TAL1
TAF7
TAF1
STAT5A
SRF
SP1
SIX5
SIRT6
Sin3Ak-20
SIN3A
SAP30
RUNX3
RBBP5
PU.1
POU2F2
Pol2(b)
Pol2-4H8
Pol2
PML
PLU1
PHF8
Pbx3
PAX5-N19
NFIC
NFATC1
NELFe
Mxi1
MTA3
MEF2C
MAZ
Max
MafK
MafF
Ini1
IKZF1
HMGN3
HDAC6
HDAC1
GTF2F1
GTF2B
FOSL1
ELK1
ELF1
Egr-1
eGFP-JunD
eGFP-JunB
eGFP-FOS
EBF1
E2F6
CTCFL
CHD2
CHD1
CCNT2
c-Jun
Brg1
BHLHE40
BCLAF1
BCL11A
ATF2
ATF1
Jim_crow_birth
parent_educ_low
parent_educ_mid
educ_low
educ_mid
hh_inc_pov
ICEraceinc
LAC
NOx
mds_0
mds_3plus
EWAS analysis
Log Odds Ratio
2
1
0
-1
-2

MESA white NH participants

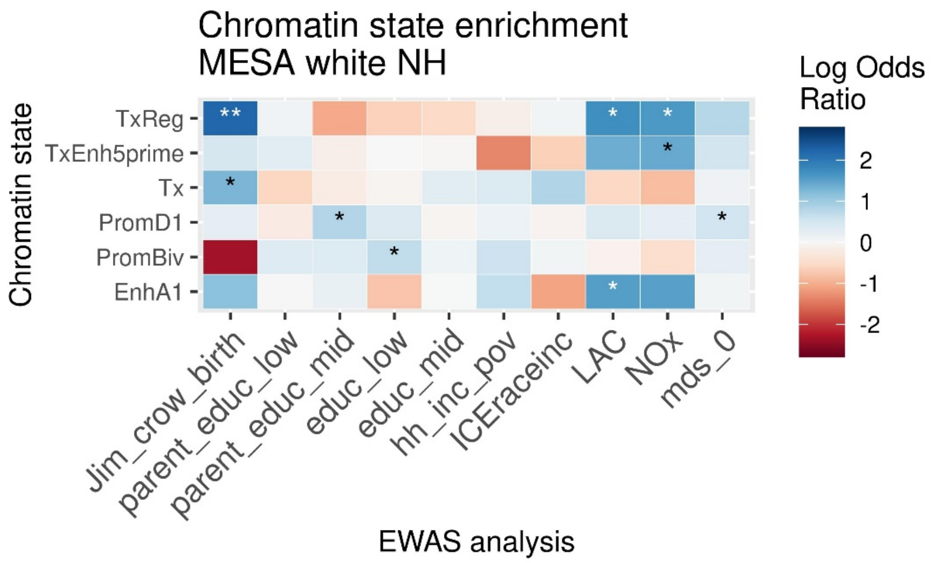

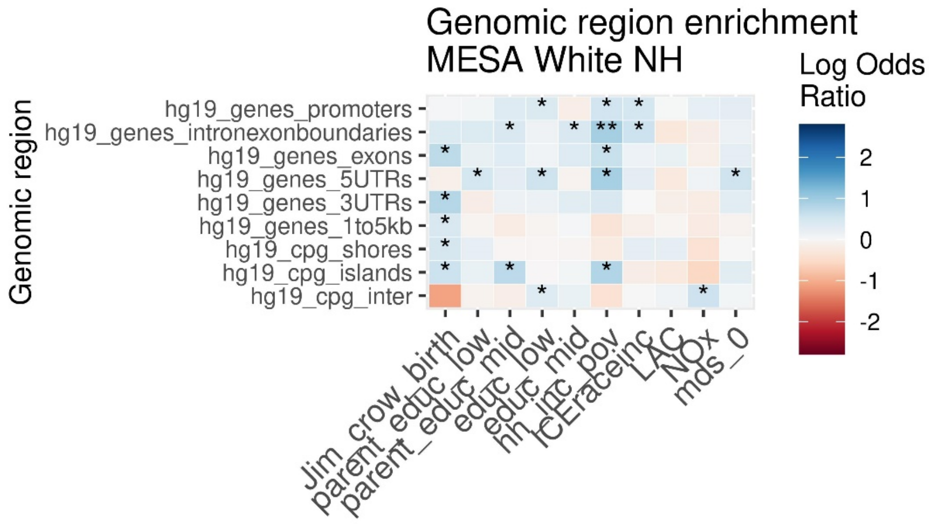

Genomic region enrichment
MESA White NH
Log Odds Ratio
2
1
0
-1
-2
Genomic region
hg19_genes_promoters
hg19_genes_intronexonboundaries
hg19_genes_exons
hg19_genes_5UTRs
hg19_genes_3UTRs
hg19_genes_1to5kb
hg19_cpg_shores
hg19_cpg_islands
hg19_cpg_inter
Jim_crow_birth
parent_educ_low
parent_educ_mid
educ_low
educ_mid
hh_inc_pov
ICEraceinc
LAC
NOx
mds_0
EWAS analysis

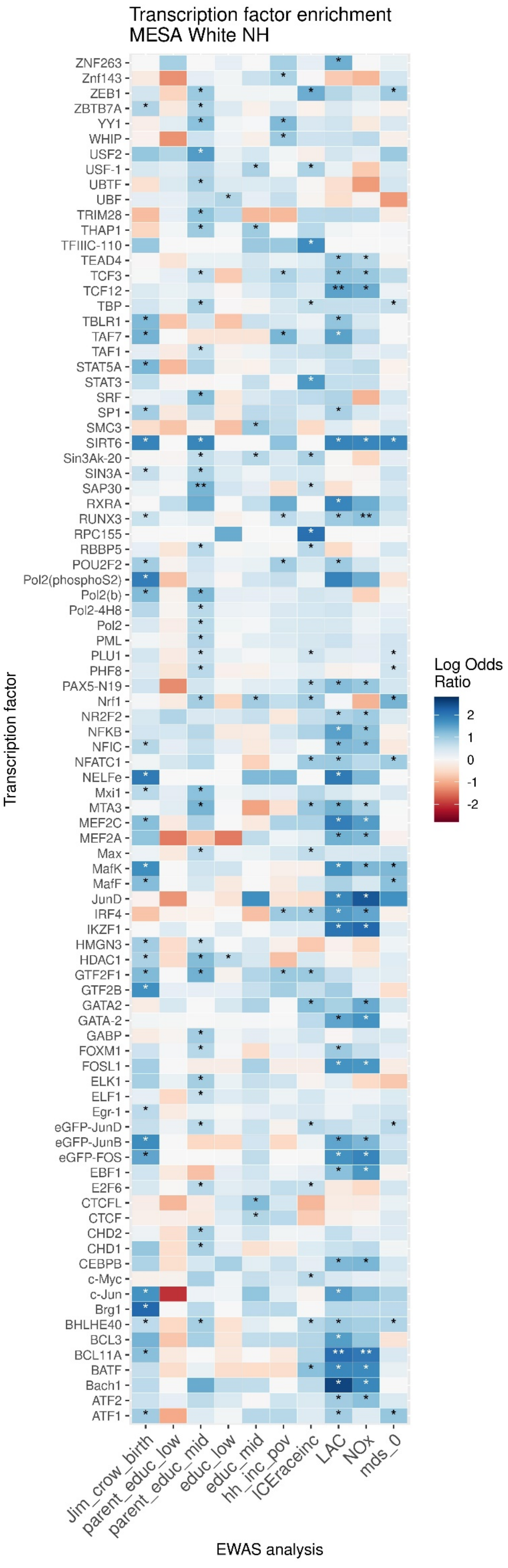

Transcription factor enrichment
MESA White NH
Transcription factor
ZNF263
Znf143
ZEB1
ZBTB7A
YY1
WHIP
USF2
USF-1
UBTF
UBF
TRIM28
THAP1
TFIIIC-110
TEAD4
TCF3
TCF12
TBP
TBLR1
TAF7
TAF1
STAT5A
STAT3
SRF
SP1
SMC3
SIRT6
Sin3Ak-20
SIN3A
SAP30
RXRA
RUNX3
RPC155
RBBP5
POU2F2
Pol2(phosphoS2)
Pol2(b)
Pol2-4H8
Pol2
PML
PLU1
PHF8
PAX5-N19
Nrf1
NR2F2
NFKB
NFIC
NFATC1
NELFe
Mxi1
MTA3
MEF2C
MEF2A
Max
MafK
MafF
JunD
IRF4
IKZF1
HMGN3
HDAC1
GTF2F1
GTF2B
GATA2
GATA-2
GABP
FOXM1
FOSL1
ELK1
ELF1
Egr-1
eGFP-JunD
eGFP-JunB
eGFP-FOS
EBF1
E2F6
CTCFL
CTCF
CHD2
CHD1
CEBPB
c-Myc
c-Jun
Brg1
BHLHE40
BCL3
BCL11A
BATF
Bach1
ATF2
ATF1
Jim_crow_birth
parent_educ_low
parent_educ_mid
educ_low
educ_mid
hh_inc_pov
ICEraceinc
LAC
NOx
mds_0
EWAS analysis
Log Odds
Ratio
2
1
0
-1
-2

MESA Hispanic participants

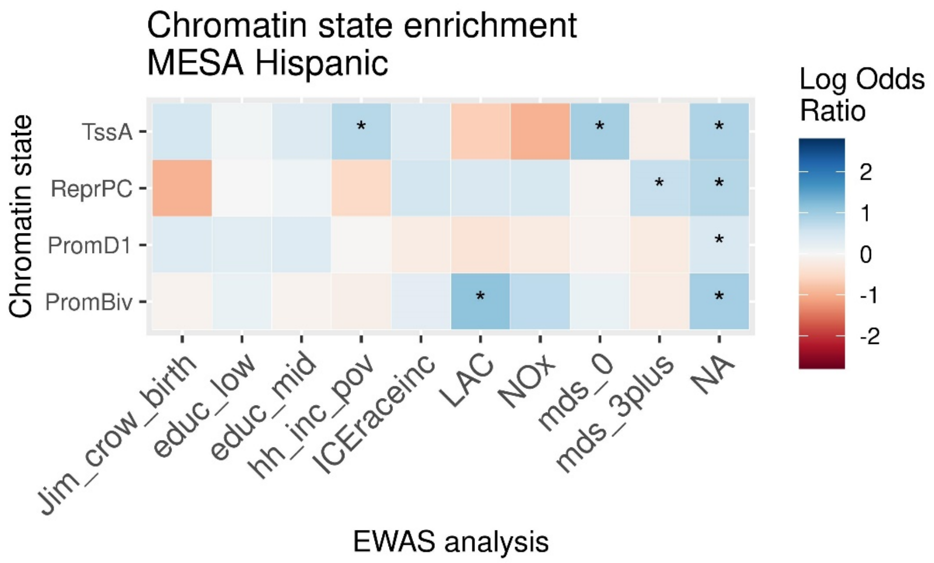

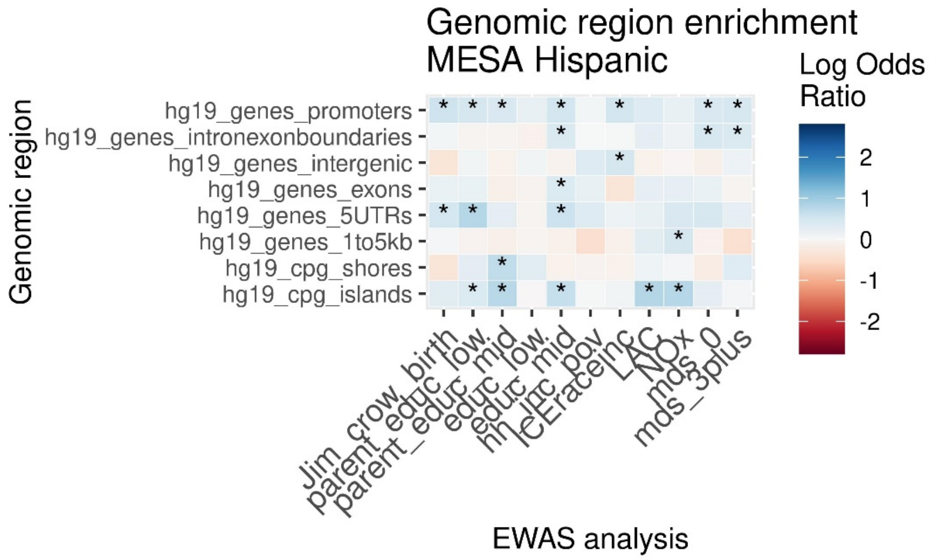
Genomic region enrichment
MESA Hispanic
Log Odds Ratio
2
1
0
-1
-2
Genomic region
hg19_genes_promoters
hg19_genes_intronexonboundaries
hg19_genes_intergenic
hg19_genes_exons
hg19_genes_5UTRs
hg19_genes_1to5kb
hg19_cpg_shores
hg19_cpg_islands
Jim_crow_birth
parent_educ_low
parent_educ_mid
educ_low
educ_mid
hh_inc_pov
ICEraceinc
LAC
NOx
mds_0
mds_3plus
EWAS analysis

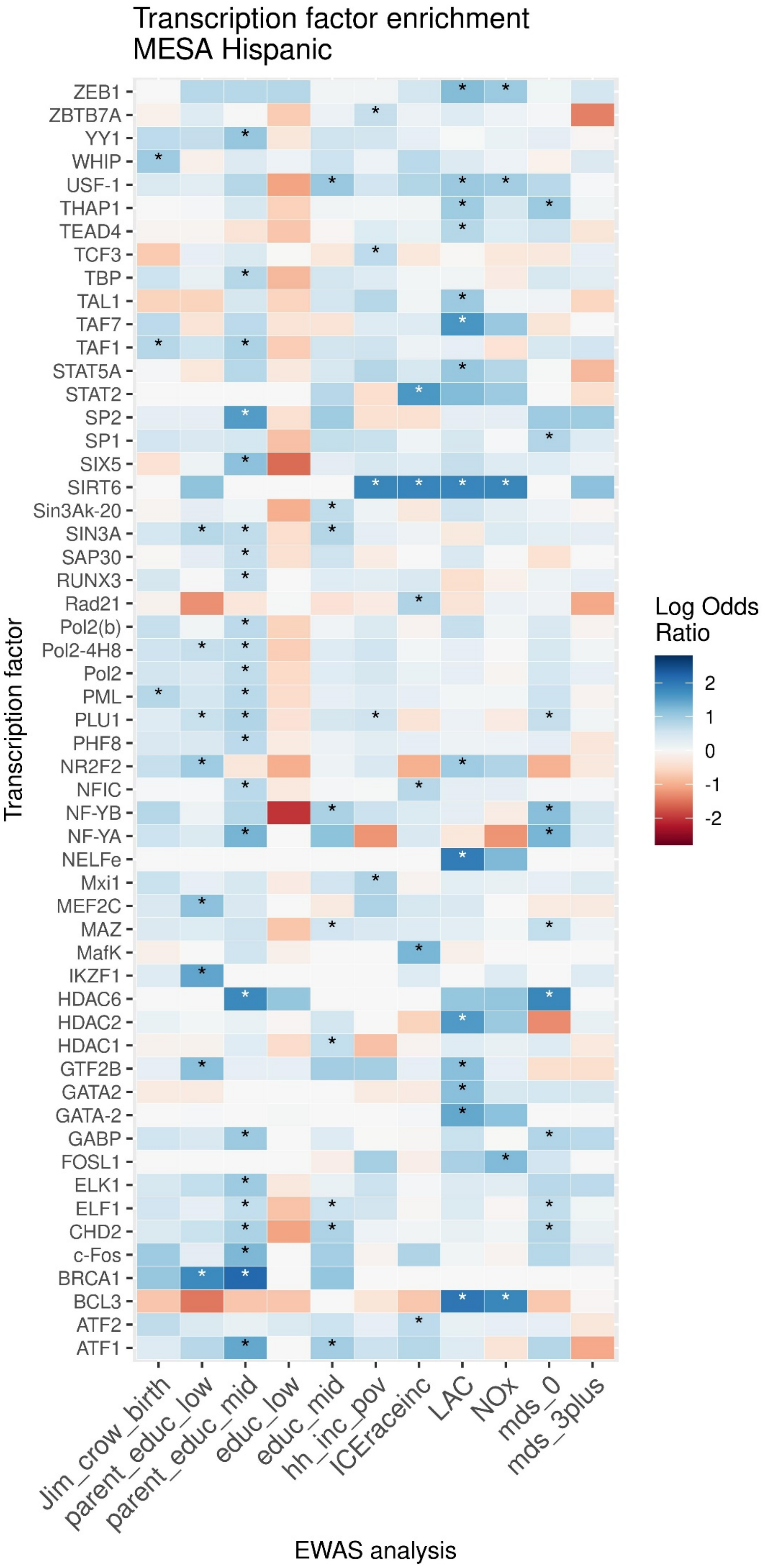
Transcription factor enrichment
MESA Hispanic
Transcription factor
ZEB1
ZBTB7A
YY1
WHIP
USF-1
THAP1
TEAD4
TCF3
TBP
TAL1
TAF7
TAF1
STAT5A
STAT2
SP2
SP1
SIX5
SIRT6
Sin3Ak-20
SIN3A
SAP30
RUNX3
Rad21
Pol2(b)
Pol2-4H8
Pol2
PML
PLU1
PHF8
NR2F2
NFIC
NF-YB
NF-YA
NELFe
Mxi1
MEF2C
MAZ
MafK
IKZF1
HDAC6
HDAC2
HDAC1
GTF2B
GATA2
GATA-2
GABP
FOSL1
ELK1
ELF1
CHD2
c-Fos
BRCA1
BCL3
ATF2
ATF1
Jim_crow_birth
parent_educ_low
parent_educ_mid
educ_low
educ_mid
hh_inc_pov
ICEraceinc
LAC
NOx
mds_0
mds_3plus
EWAS analysis
Log Odds
Ratio
2
1
0
-1
-2

MESA Black NH participants (JHU + COL subgroup)

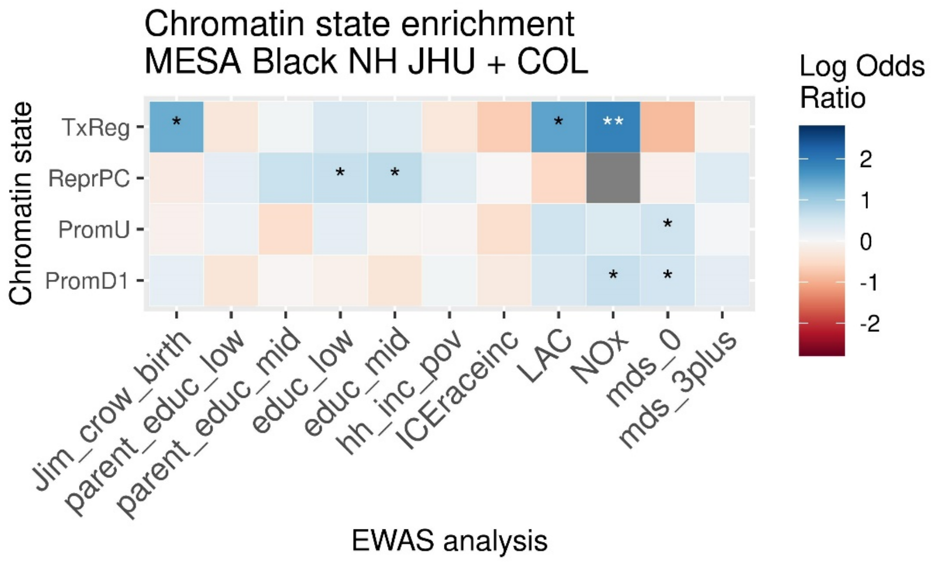

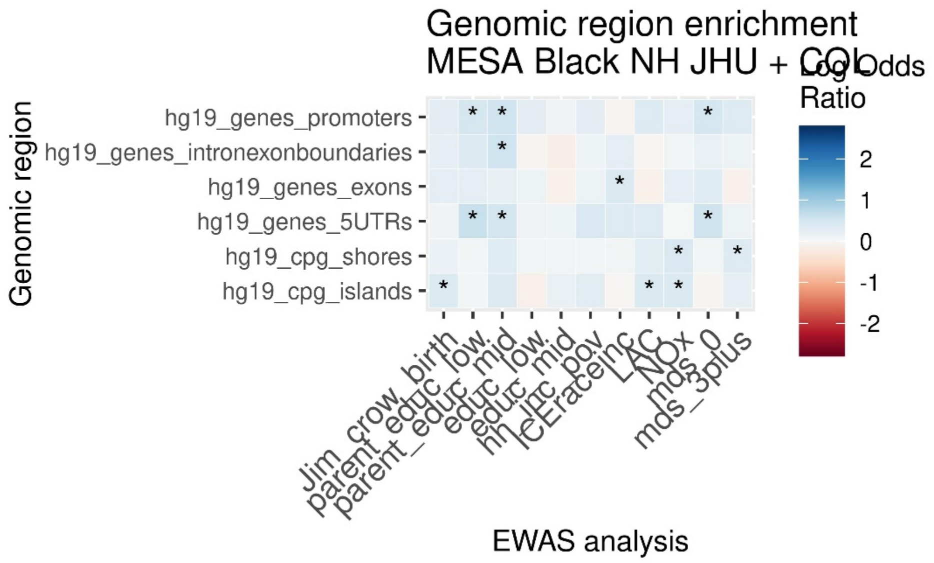

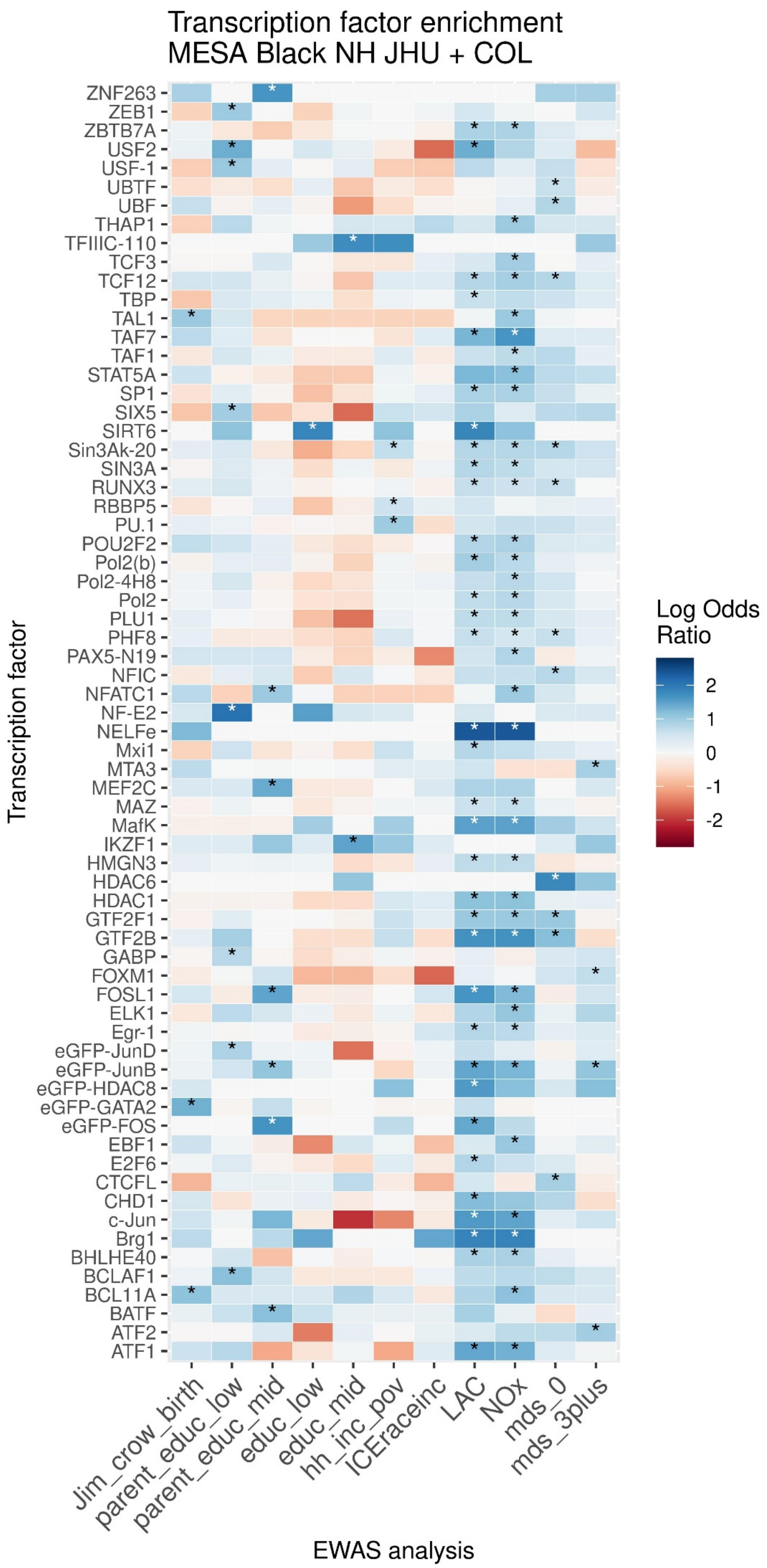
Transcription factor enrichment
MESA Black NH JHU + COL
Transcription factor
ZNF263
ZEB1
ZBTB7A
USF2
USF-1
UBTF
UBF
THAP1
TFIIIC-110
TCF3
TCF12
TBP
TAL1
TAF7
TAF1
STAT5A
SP1
SIX5
SIRT6
Sin3Ak-20
SIN3A
RUNX3
RBBP5
PU.1
POU2F2
Pol2(b)
Pol2-4H8
Pol2
PLU1
PHF8
PAX5-N19
NFIC
NFATC1
NF-E2
NELFe
Mxi1
MTA3
MEF2C
MAZ
MafK
IKZF1
HMGN3
HDAC6
HDAC1
GTF2F1
GTF2B
GABP
FOXM1
FOSL1
ELK1
Egr-1
eGFP-JunD
eGFP-JunB
eGFP-HDAC8
eGFP-GATA2
eGFP-FOS
EBF1
E2F6
CTCFL
CHD1
c-Jun
Brg1
BHLHE40
BCLAF1
BCL11A
BATF
ATF2
ATF1
Jim_crow_birth
parent_educ_low
parent_educ_mid
educ_low
educ_mid
hh_inc_pov
ICEraceinc
LAC
NOx
mds_0
mds_3plus
EWAS analysis
Log Odds Ratio
2
1
0
-1
-2

MESA white NH participants (JHU + COL subgroup)

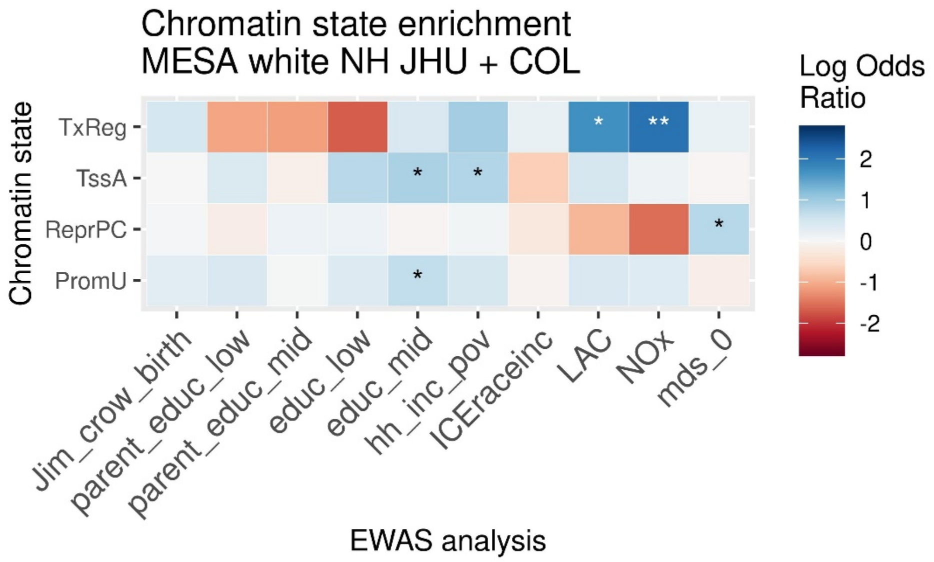

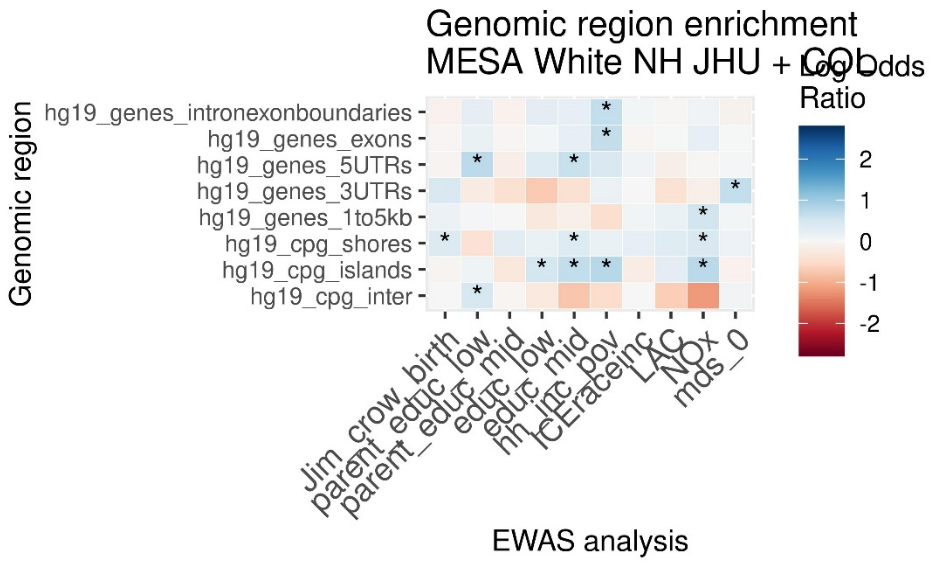

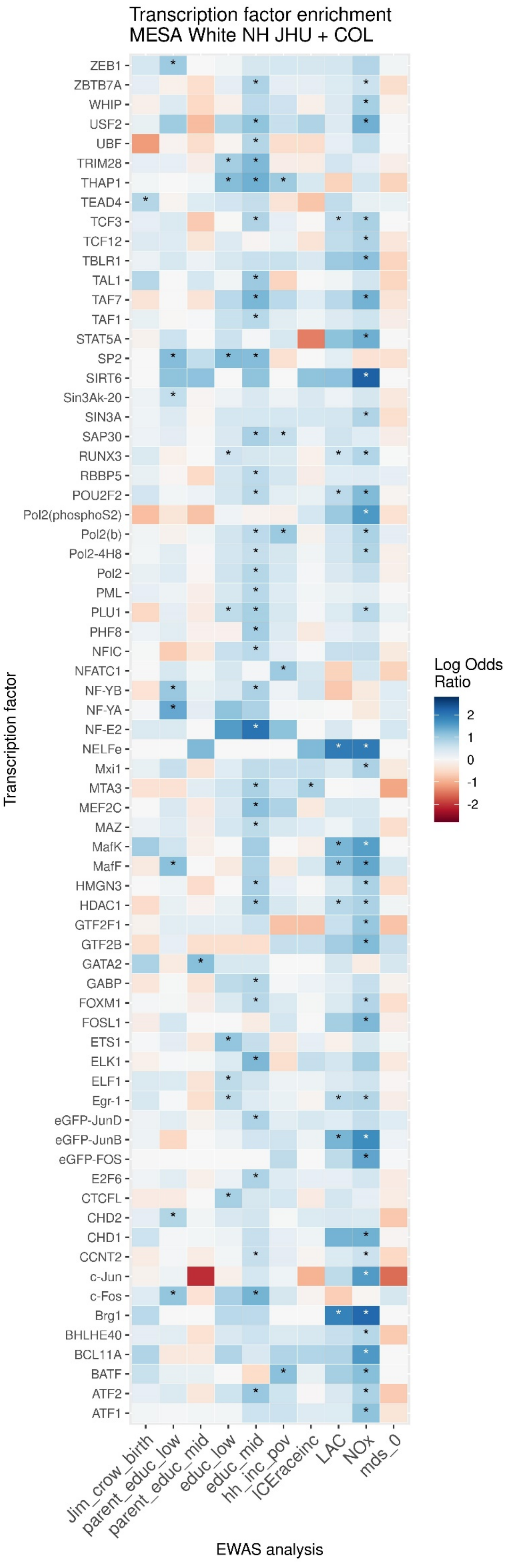

Transcription factor enrichment
MESA White NH JHU + COL
Transcription factor
ZBTB7A
ZEB1
WHIP
USF2
UBF
TRIM28
THAP1
TEAD4
TCF3
TCF12
TBLR1
TAL1
TAF7
TAF1
STAT5A
SP2
SIRT6
Sin3Ak-20
SIN3A
SAP30
RUNX3
RBBP5
POU2F2
Pol2(phosphoS2)
Pol2(b)
Pol2-4H8
Pol2
PML
PLU1
PHF8
NFIC
NFATC1
NF-YB
NF-YA
NF-E2
NELFe
Mxi1
MTA3
MEF2C
MAZ
MafK
MafF
HMGN3
HDAC1
GTF2F1
GTF2B
GATA2
GABP
FOXM1
FOSL1
ETS1
ELK1
ELF1
Egr-1
eGFP-JunD
eGFP-JunB
eGFP-FOS
E2F6
CTCFL
CHD2
CHD1
CCNT2
c-Jun
c-Fos
Brg1
BHLHE40
BCL11A
BATF
ATF2
ATF1
Jim_crow_birth
parent_educ_low
parent_educ_mid
educ_low
educ_mid
hh_inc_pov
ICEraceinc
LAC
NOx
mds_0
EWAS analysis
Log Odds Ratio
2
1
0
-1
-2

MESA Hispanic participants (JHU + COL subgroup)

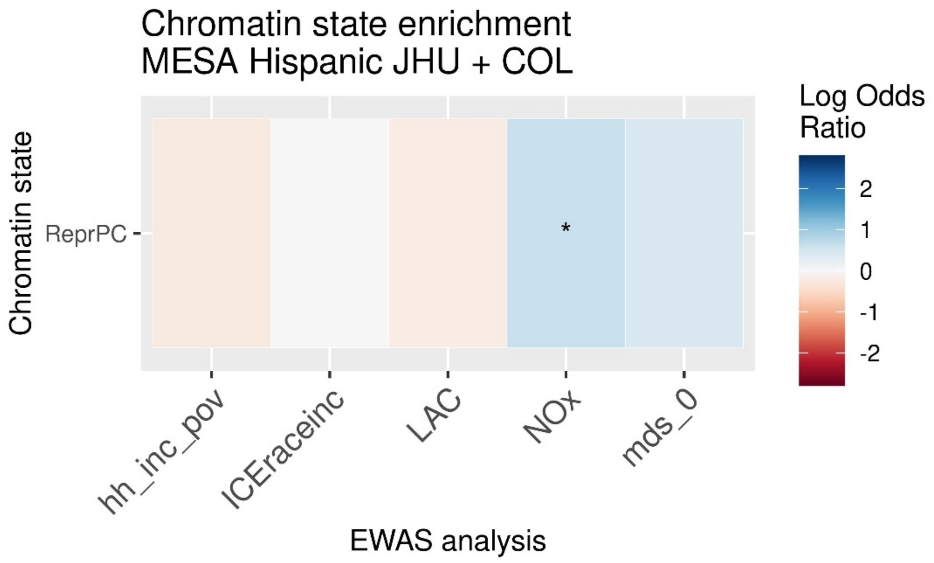

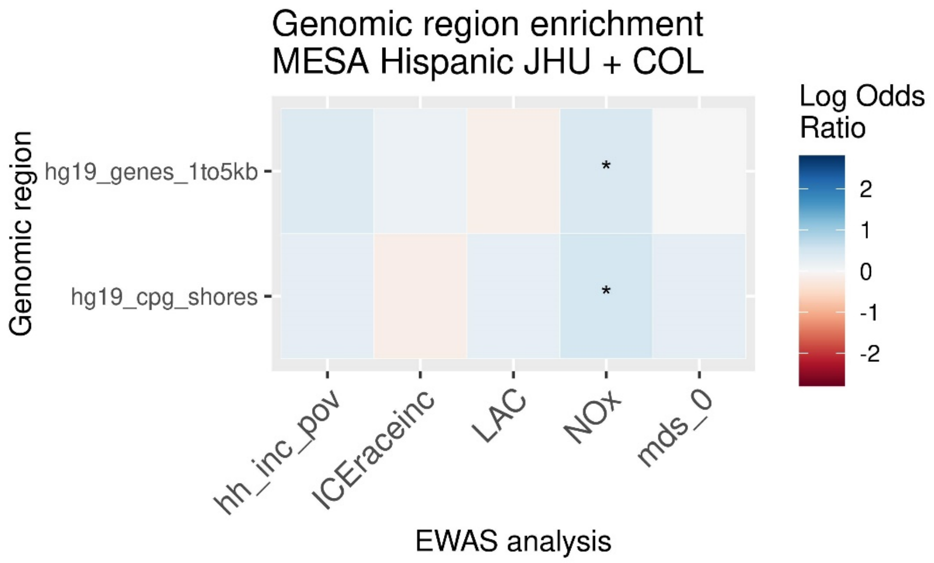

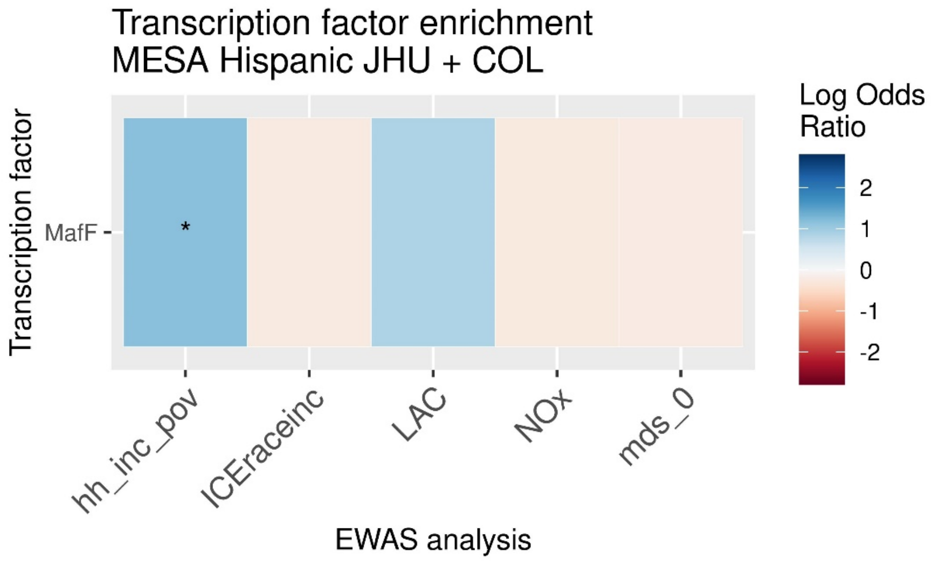

MBMS meta-analysis

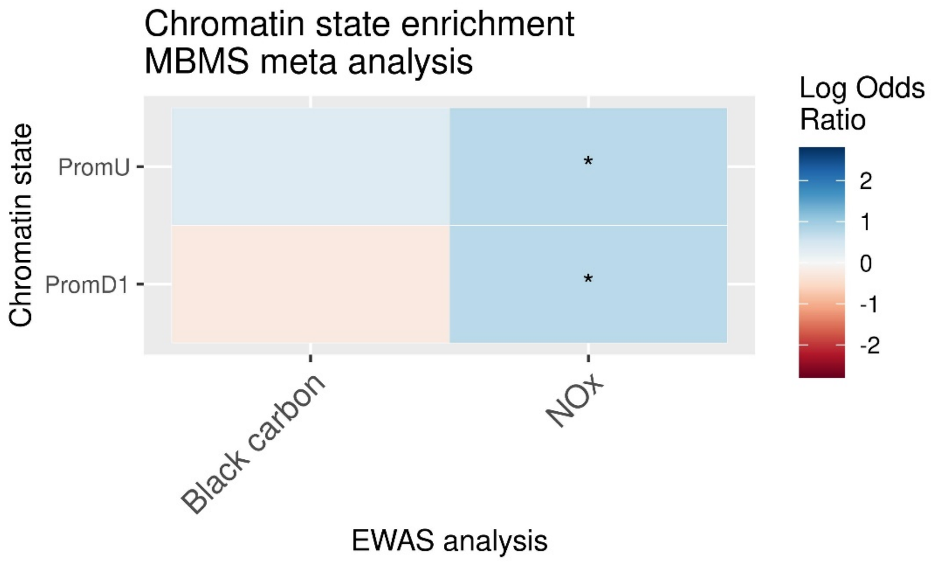

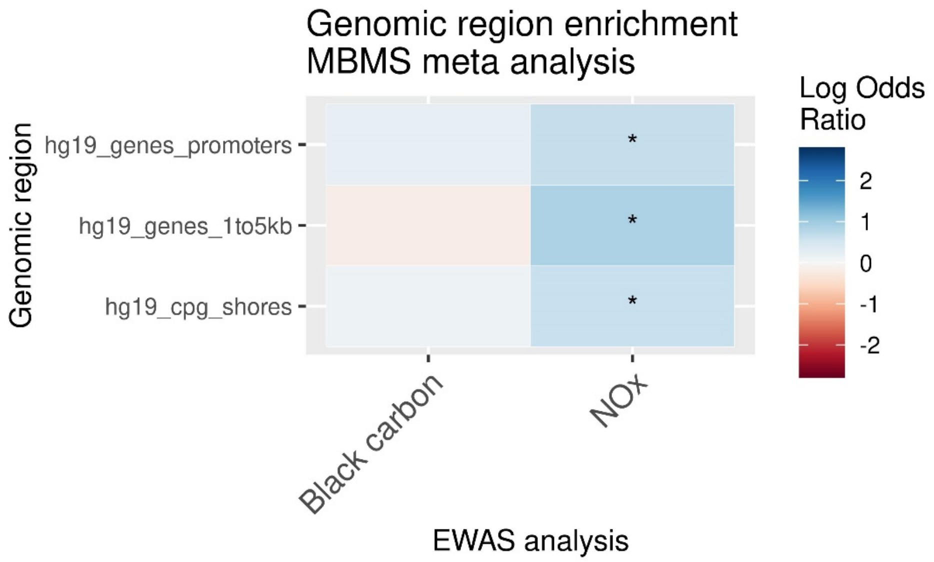

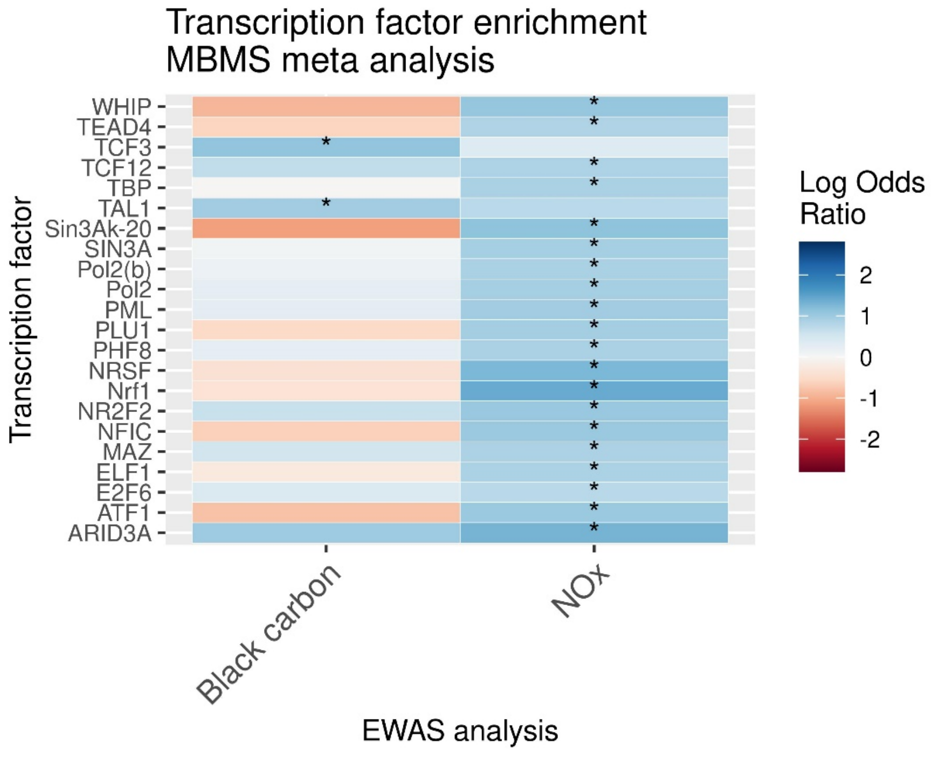

Transcription factor enrichment
MBMS meta analysis
Transcription factor
WHIP
TEAD4
TCF3
TCF12
TBP
TAL1
Sin3Ak-20
SIN3A
Pol2(b)
Pol2
PML
PLU1
PHF8
NRSF
Nrf1
NR2F2
NFIC
MAZ
ELF1
E2F6
ATF1
ARID3A
Black carbon
NOx
EWAS analysis
Log Odds
Ratio
2
1
0
-1
-2

Mesa meta-analysis (full cohort)

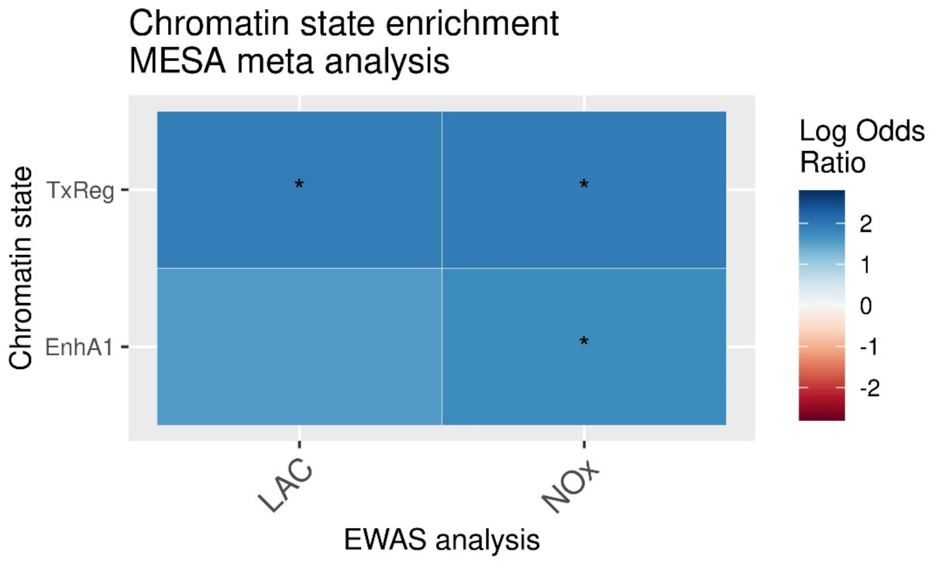

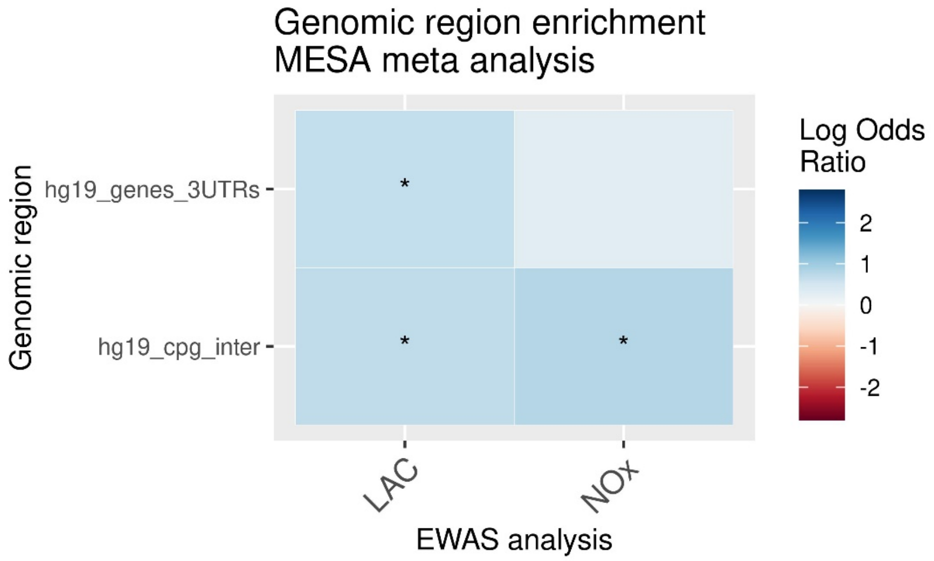

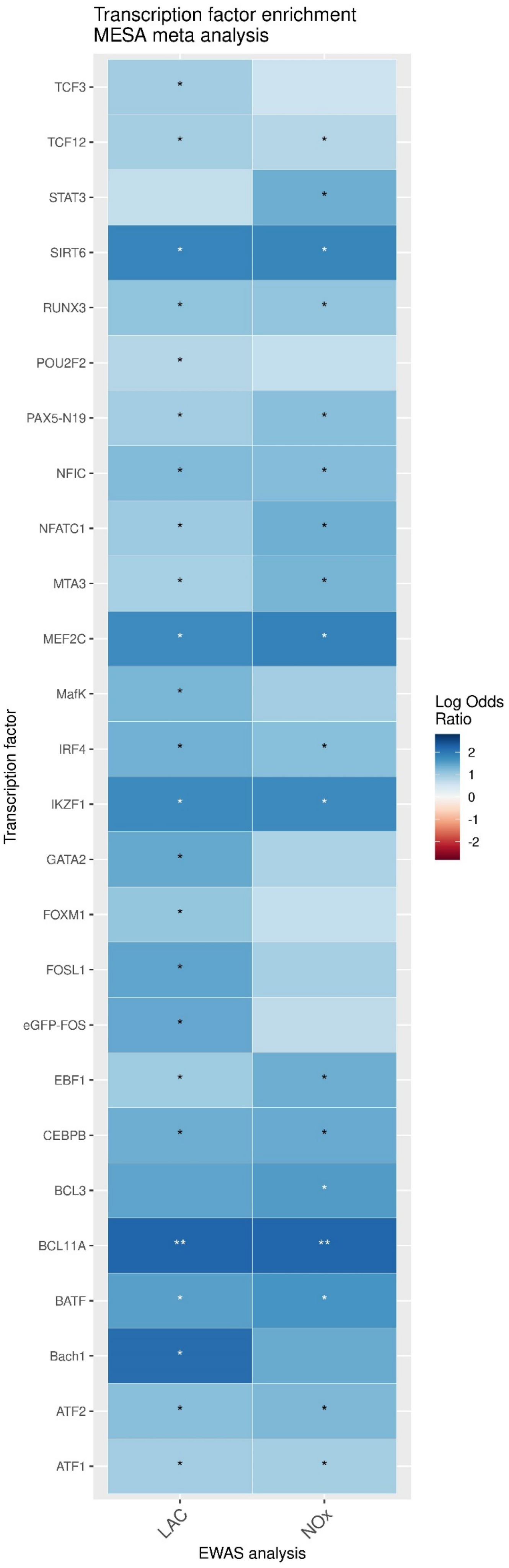
Transcription factor enrichment
MESA meta analysis
Transcription factor
TCF3
TCF12
STAT3
SIRT6
RUNX3
POU2F2
PAX5-N19
NFIC
NFATC1
MTA3
MEF2C
MafK
IRF4
IKZF1
GATA2
FOXM1
FOSL1
eGFP-FOS
EBF1
CEBPB
BCL3
BCL11A
BATF
Bach1
ATF2
ATF1
LAC
NOx
EWAS analysis
Log Odds
Ratio
2
1
0
-1
-2

MESA meta-analysis (JHU + COL subgroup)

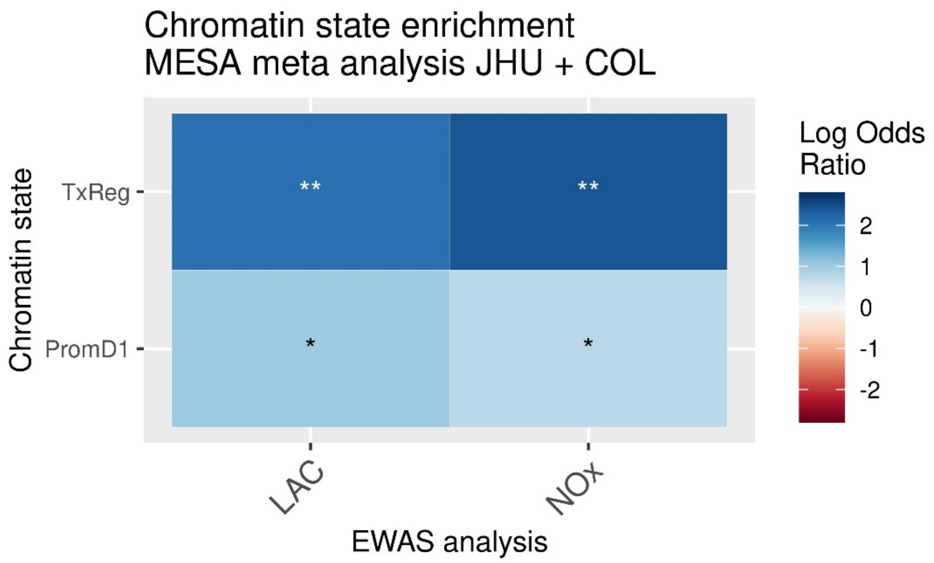

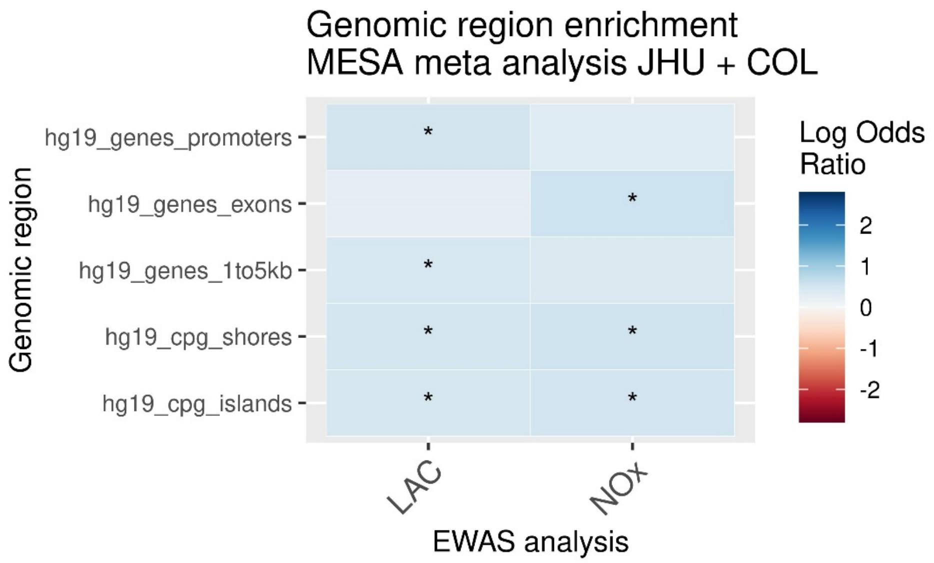

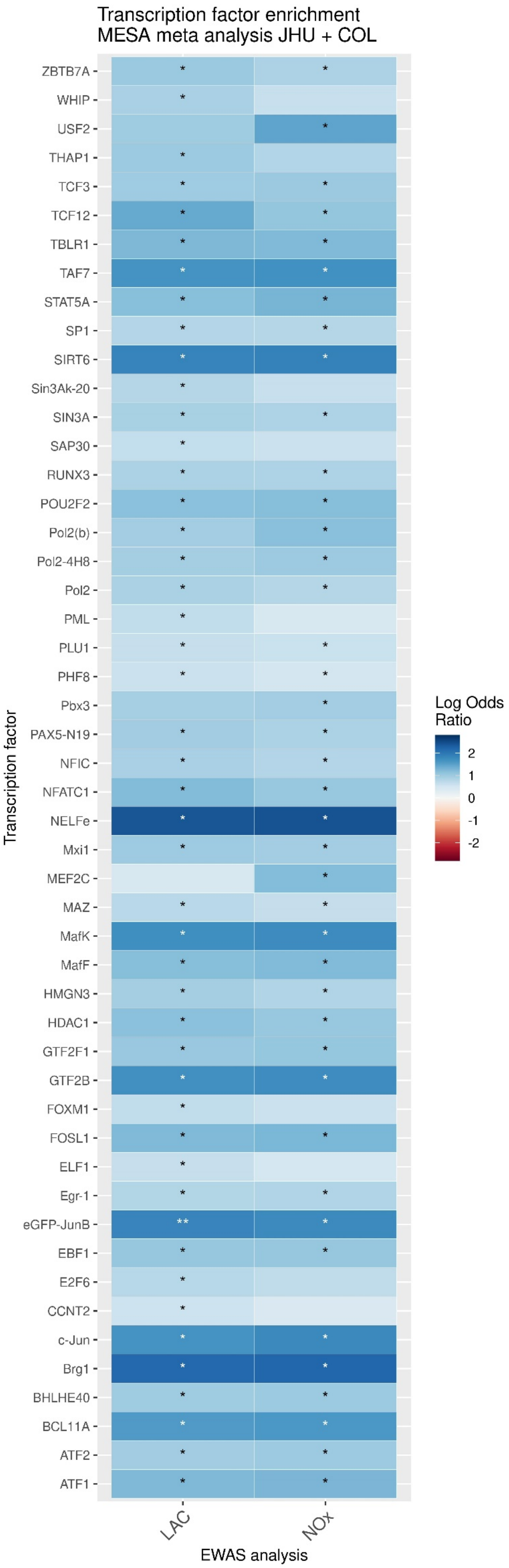
Transcription factor enrichment
MESA meta analysis JHU + COL
Transcription factor
ZBTB7A
WHIP
USF2
THAP1
TCF3
TCF12
TBLR1
TAF7
STAT5A
SP1
SIRT6
Sin3Ak-20
SIN3A
SAP30
RUNX3
POU2F2
Pol2(b)
Pol2-4H8
Pol2
PML
PLU1
PHF8
Pbx3
PAX5-N19
NFIC
NFATC1
NELFe
Mxi1
MEF2C
MAZ
MafK
MafF
HMGN3
HDAC1
GTF2F1
GTF2B
FOXM1
FOSL1
ELF1
Egr-1
eGFP-JunB
EBF1
E2F6
CCNT2
c-Jun
Brg1
BHLHE40
BCL11A
ATF2
ATF1
LAC
NOx
EWAS analysis
Log Odds Ratio
2
1
0
-1
-2
