# Supplementary material for: An epigenome-wide analysis of DNA methylation, racialized and economic inequities, and air pollution
Source: Clin Epigenetics. 2025 Nov 27;18:4. doi: 10.1186/s13148-025-01929-6 (PMC12764144; doi:10.1186/s13148-025-01929-6)
Supplement: Supplementary file 5 — Additional file5 (DOCX 195 KB) [file 13148_2025_1929_MOESM5_ESM.docx]

# Supplementary materials

# Methods

## Participants

MBMS includes participants recruited from four Community Health Centers (CHCs) in Boston, MA between 2008 and 2010, and was designed to investigate the association between racial discrimination and risk of cardiovascular disease, taking into account a range of social and environmental factors. The MBMS cohort and recruitment procedures have previously been described in detail (Krieger, Waterman, et al., 2011); briefly, the study recruited 1005 individuals who met study inclusion criteria and were randomly selected from the patient rosters of the CHCs. Participants were eligible if they were aged between 35 and 64 years, had been born in the US, and self-identified as white non-Hispanic or Black non-Hispanic.

Among the 1005 MBMS participants, 85% provided a finger prick blood sample on to filter paper (409 Black; 466 White), and consequently biological material was limited and in some instances of poor quality. Blood spots were stored at -20°C, and DNA was extracted from blood spots using the QIAamp DNA Investigator Kit for FTA and Guthrie cards, with samples randomised across 96 well plates. Of the 875 participants who provided blood spots, 472 of the samples were judged to be suitable for DNA extraction (blood spots judged not to be suitable were primarily collected at the first CHC where recruitment took place, whose membership was predominantly white). Of those, 48 yielded less than 40ng of DNA; we have previously determined this to be an input level at which data quality decreases (Watkins et al., 2022), so we removed them from further analysis. The amount of DNA extracted was assessed using Invitrogen Quant-iT™ PicoGreen™ (Thermo Fisher Scientific).

We generated DNAm data using the Illumina Infinium MethylationEPIC BeadChip for the remaining 424 participants. We then removed a further 96 participants from the sample set due to poor quality DNA extraction (as determined by high numbers of undetected probes on the EPIC BeadChip; these 96 samples had substantially lower levels of DNA than the 328 remaining for analysis (means of 136.8ng vs 220.7ng; t-test p=1.9e09)). Another 35 samples had a mismatch between the gender they self-reported in the study and sex as predicted by probe signal intensities targeting sites on the X and Y chromosomes. These mismatches were likely due to erroneous sample swaps. We confirmed this by showing that, by showing that, whereas chronological age and age estimated from DNA methylation were methylation highly correlated among the 293 samples whose self-reported gender matched their probe intensity (Horvath clock R=0.63, Hannum clock R=0.69), correlation among the 35 with a mismatch was very low (Horvath clock R=-0.01, Hannum clock R=0.18). This left us with 293 participants (224 Black and 69 white) with DNA methylation data for analysis.

MESA is a longitudinal US-based cohort that was set up to investigate subclinical cardiovascular disease in individuals free of clinical cardiovascular disease at recruitment. MESA comprises a total of 6814 participants aged 45-84, who were recruited to the study from six field centers across the US. We utilise data from a random subset of participants recruited from four of the field centers (Baltimore, MD; Forsyth County, NC; New York City, NY; and St. Paul, MN) who had blood samples collected at the Exam 5 data collection (2010-2012), and DNAm assayed using the Illumina Infinium HumanMethylation450 BeadChip (total n=1264, aged 55-94 at time of blood draw). DNAm was measured in monocytes isolated from whole blood with over 90% purity (Liu et al., 2013). To ensure an appropriate comparison dataset to MBMS, we included the 975 participants who were US-born and self-identified as non-Hispanic Black (n=229) and non-Hispanic white (n=555), additionally including individuals who identified as Hispanic (n=191).

We utilised only US-born MESA participants because research indicates these groups systematically differ in their self-reports of racial discrimination, with responses in the latter group differential by age at immigration and duration in the US (Brondolo et al., 2015; Dominguez, Strong, Krieger, Gillman, & Rich-Edwards, 2009; Krieger, Kosheleva, Waterman, Chen, & Koenen, 2011). Recruitment of the three racialized groups was uneven across the four recruitment sites; please see Supplementary Table 1 for a breakdown of numbers.

|  | Approximate equivalent to Boston | |  | |
| --- | --- | --- | --- | --- |
|  | New York city, NY | Baltimore, MD | St Paul, MN | Forsyth County, NC |
| Black N.H. | 93 | 132 | 0 | 4 |
| Hispanic | 67 | 0 | 124 | 0 |
| White N.H. | 71 | 168 | 270 | 46 |

Supplementary Table 1: breakdown of racialized group recruitment from the 4 MESA sites that generated DNAm data; for the 975 US-born participants only.

## Sensitivity analysis

We tested whether our results were influenced by population stratification by additionally adjusting each EWAS for the first 10 genetic principal components (PCs), and then using Pearson correlation to test effect size replication for the top 10, 25, 50, 100 and 200 sites in each EWAS. MESA used the Affymetrix Genome-Wide Human SNP Array 6.0 to genotype 8402 participants; this included 933 out of 975 of the US-born participants with DNAm data. Genetic principal components were generated by MESA: 23,428 flagged SNPs and 6,849 SNPs in long range LD were removed before principal components were computed per chromosome, and then combined across chromosomes to give the final PC values. We used the genetic PCs estimated in participants stratified by self-reported racialized group, to match our own study setup.

## Biological enrichments of top 100 sites

These analyses included the top 100 sites from each EWAS to ensure were sufficient numbers to assess enrichment. Gene set enrichment analyses were conducted using the R package *missMethyl*. Enrichments among sites previously associated with other phenotypes and exposures were tested by comparison to published EWAS summary statistics recorded by the EWAS catalog (Battram et al., 2022) using previously described methods to create phenotype and exposure categories (Elliott et al., 2022). Enrichment of up to 26 categories could be tested (categories were not tested if no CpGs in the category were in the test set of sites); between 8 and 19 categories were tested for our exposures. Enrichments for tissue-specific chromatin states, genomic regions and transcription factor binding sites (TFBS) were assessed the R package *LOLA* (Sheffield & Bock, 2016). We tested for enrichment of chromatin states using the Roadmap Epigenomics chromHMM imputed 25 chromatin states (Ernst & Kellis, 2015; Roadmap Epigenomics et al., 2015); for genomic regions using Illumina annotations (Zhou, Laird, & Shen, 2017); and for TFBS using the Encode (Consortium, 2012; Davis et al., 2018) TFBS set, comprising ChIP-seq data on 161 TFs. All sets of genomic loci are available through <http://lolaweb.databio.org>; we reduced these to features measured using blood. For all LOLA analyses, each DNAm site was extended to a 200bp region centred at the site, removing overlapping sites to prevent inflation.

## Lookup of associations in *a priori* specified genomic locations

We hypothesised a priori that our EWASs would detect DNAm sites that have been robustly associated with our study exposures, or factors that might relate to our exposures, in previous studies. Because some of the structural measures have not been tested via EWAS before, we also included hypothesised pathways. We grouped previous studies as follows with regard to our exposures: parent’s and participant’s education (previous EWAS of education), household poverty to income ratio (previous EWAS of individual-level socioeconomic status), ICE race plus income (previous EWAS of neighbourhood level socioeconomic measures), black carbon, LAC, and NOx (previous EWAS of air pollution measures), and EOD and MDS (previous EWAS of experiences of racial discrimination). No equivalents were available for Jim Crow birth state. We included studies that included at least 100 participants, and restricted sites to those passing the genome-wide threshold (2.4e-7). Where studies only reported FDR, we used the study FDR threshold. DNAm sites associated with these domains in previous literature were identified through the EWAS catalog and literature searches. Where publications reported genes rather than CpG sites, we took all CpG sites within 1000bp of the gene (to incorporate transcription start sites and promotors). To ensure the sites we tested had robust associations with the exposures, we only took DNAm sites forward where they were identified in at least two separate studies, aside from racial discrimination where literature is currently limited, as this was an important focus of our study. EWAS results for each of the tested exposures were then reduced to the sites that had been identified for that particular exposure in previous literature, and tested for association at p<0.05 divided by the number of sites tested. The script containing code to replicate this list of DNAm sites can be found on our [GitHub](https://github.com/shwatkins/EWAS_of_social_inequities) page.

# Results

Predicted cell type proportions are detailed in Supplementary Table 2; MBMS was measured in whole blood, but as MESA measured DNAm in purified monocytes it is difficult to interpret any differences.

| **Predicted cell count proportions** | | | | | |
| --- | --- | --- | --- | --- | --- |
| B Cell | 0.08 (0.02) | 0.06 (0.01) | 0.04 (0.03) | 0.03 (0.02) | 0.03 (0.02) |
| CD4+T cells | 0.17 (0.05) | 0.15 (0.04) | 0.04 (0.02) | 0.03 (0.03) | 0.03 (0.01) |
| CD8+T cells | 0.005 (0.02) | 0.002 (0.01) | 0.002 (0.006) | 0.0007 (0.003) | 0.0006 (0.003) |
| Monocytes | 0.124 (0.02) | 0.116 (0.02) | 0.9 (0.05) | 0.91 (0.04) | 0.92 (0.04) |
| Neutrophils | 0.55 (0.1) | 0.62 (0.08) | 0.0003 (0.002) | 0.0008 (0.005) | 0.0009 (0.007) |
| Natural Killer | 0.1 (0.04) | 0.09 (0.04) | 0.015 (0.01) | 0.012 (0.01) | 0.01 (0.01) |
| Eosinophils | 0.007 (0.02) | 0.003 (0.009) | 0.01 (0.01) | 0.01 (0.01) | 0.01 (0.01) |

Table 2: predicted cell type proportions in MBMS and MESA. Predictions were made using the meffil R package, using the “blood gse35069 complete reference” (Houseman et al., 2012)

## EWAS results and biological interpretation

We noted that there was a strong overlap between the genome-wide significant DNAm sites between the Jim Crow birth state and the air pollution EWASs in both the Black and white non-Hispanic MESA participants. The sites at which MESA participants were recruited strongly determined their air pollution exposure (with the Columbia site in New York having much higher pollution levels than other sites); and recruitment site was strongly related to whether or not individuals were born in a Jim Crow state (the more southern recruitment sites recruiting more individuals born in Southern states with Jim Crow laws). We suspected that the associations in the Jim Crow EWAS in the white NH participants were very likely to have been mediated by air pollution; so we re-performed the air pollution EWAS analyses among the MESA White NH participants stratified by Jim Crow birth status. We found that effect sizes of these stratified EWAS correlated R = 0.86-0.99 with the original air pollution EWAS (which we took to be good replication, given the lower numbers and reduction in variation in air pollution in the stratified groups). This suggests that the associations in the Jim Crow EWAS for the MESA White NH participants were mediated by air pollution, resulting from the intersection of the geographical distribution of air pollution, and the tendency of participants to remain living in a relatively similar geographical area to that which they were born in.

## Sensitivity analysis

We utilised the first 10 genetic PCs in MESA to test whether population stratification might play a role in our EWAS findings. Additionally adjusting for genetic PCs in our EWAS models, we found that effect sizes for the top 10, 25, 50, 100 and 200 sites from each exposure EWAS correlated with our original EWAS: R>0.99 for white NH MESA participants; R>0.97 for MESA black NH participants; and R>0.98 for MESA Hispanic participants, except for R=0.90 for low education. For EWAS test statistics (t statistic), we found the top 10, 25, 50, 100 and 200 sites from each exposure EWAS correlated with our original EWAS: R>0.99 for white NH MESA participants; R>0.96 for MESA black NH participants; and R>0.98 for MESA Hispanic participants, except for R=0.91 for low education and R=0.9 for racialized economic segregation. We therefore concluded that population stratification did not drive the results of any of our EWAS. We could not run this sensitivity analysis for MBMS as this study does not have genetic data.

When we subset participants to those recruited at the Johns Hopkins and Columbia sites, we did not run the analysis for the black NH participants because only 4 participants were recruited outside of those two sites. For the white NH participants, we found that effect sizes for the top 10, 25, 50, 100 and 200 sites from each exposure EWAS correlated (using Pearson correlation) with our original EWAS R>0.93; aside from the low education (R>0.79) and air pollution (R>0.57 and R>0.73 for LAC and NOx, respectively) exposures.

## MESA subgroup analysis

To test how the similarity in air pollution sites changed when we subset MESA to the Baltimore and New York sites, we correlated the effect sizes of the top 10, 25, 50, 100 and 200 strongest CpG site associations between the Black NH and white NH groups. We see an increase in correlation between the groups in the subset analysis (see Supplementary table 3); this is potentially because the largest number of white NH participants in the MESA epigenetic substudy were recruited from the Minnesota site, whereas no Black NH participants in the MESA epigenetic substudy were recruited at that site (see Supplementary table 1).

| **Full cohort** | | | | | | | | |
| --- | --- | --- | --- | --- | --- | --- | --- | --- |
| Black NH effect sizes replication in white NH | | | | | | | | |
| Exposure | top_10 | top_25 | top_50 | top_100 | top_200 | mean | min | max |
| LAC | -0.49 | -0.49 | -0.51 | -0.13 | -0.09 | -0.34 | -0.51 | -0.09 |
| NOx | 0.86 | 0.79 | 0.71 | 0.64 | 0.55 | 0.71 | 0.55 | 0.86 |
| White NH effect sizes replication in Black NH | | | | | | | | |
| Exposure | top_10 | top_25 | top_50 | top_100 | top_200 | mean | min | max |
| LAC | 0.63 | 0.13 | 0.13 | 0.1 | 0.07 | 0.21 | 0.07 | 0.63 |
| NOx | 0.66 | 0.43 | 0.03 | 0 | 0.01 | 0.23 | 0 | 0.66 |
| **Subgroup** | | | | | | | | |
| Black NH effect sizes replication in white NH | | | | | | | | |
| Exposure | top_10 | top_25 | top_50 | top_100 | top_200 | mean | min | max |
| LAC | 0.95 | 0.91 | 0.87 | 0.76 | 0.65 | 0.83 | 0.65 | 0.95 |
| NOx | 0.97 | 0.94 | 0.94 | 0.89 | 0.55 | 0.85 | 0.55 | 0.97 |
| White NH effect sizes replication in Black NH | | | | | | | | |
| Exposure | top_10 | top_25 | top_50 | top_100 | top_200 | mean | min | max |
| LAC | 0.4 | 0.44 | 0.46 | 0.47 | 0.43 | 0.44 | 0.4 | 0.47 |
| NOx | 0.97 | 0.95 | 0.51 | 0.53 | 0.5 | 0.69 | 0.5 | 0.97 |

Supplementary Table 3: Correlation between effect sizes of the air pollution EWAS between MESA Black NH and white NH participants for 1) the full cohort and 2) the Baltimore and New York recruitment site subset

## Meta-analysis

Supplementary Table 4 shows the complete case numbers for the air pollution meta-analyses, along with the number of sites passing the genome-wide threshold. The number of associated sites increased in the subset of participants recruited at the Baltimore and New York sites even though the number of participants was cut almost by half. The two removed sites in the subgroup analysis had very low levels of air pollution – it is possible that their removal increased our ability to detect effects because it removed a large number of observations with low variance and low levels of exposure. This difference in pollution levels is illustrated by Supplementary Figure 1. The strong to moderate correlations between effect sizes of the full cohort meta-analysis and the subset meta-analysis suggest that the relationship between DNAm and exposure is relatively similar, but p-value differences might relate to the differences in sample size and the differences in exposure variability (see Supplementary Table 5 for the effect size correlations and Supplementary Table 7 for a table comparing p-values and effect sizes).

|  | **MBMS** | | **MESA** | | **MESA (JHU + COL)** | |
| --- | --- | --- | --- | --- | --- | --- |
|  | **N** | **N sites** | **N** | **N sites** | **N** | **N sites** |
| **Black carbon/LAC*** | 293 | 0 | 912 | 17 | 496 | 51 |
| **NOx** | 288 | 0 | 912 | 18 | 496 | 79 |

Supplementary Table 4: Meta-analysis results for air pollution exposure in MBMS and MESA. *Black carbon measured in MBMS; LAC (light absorption coefficient) measured in MESA.


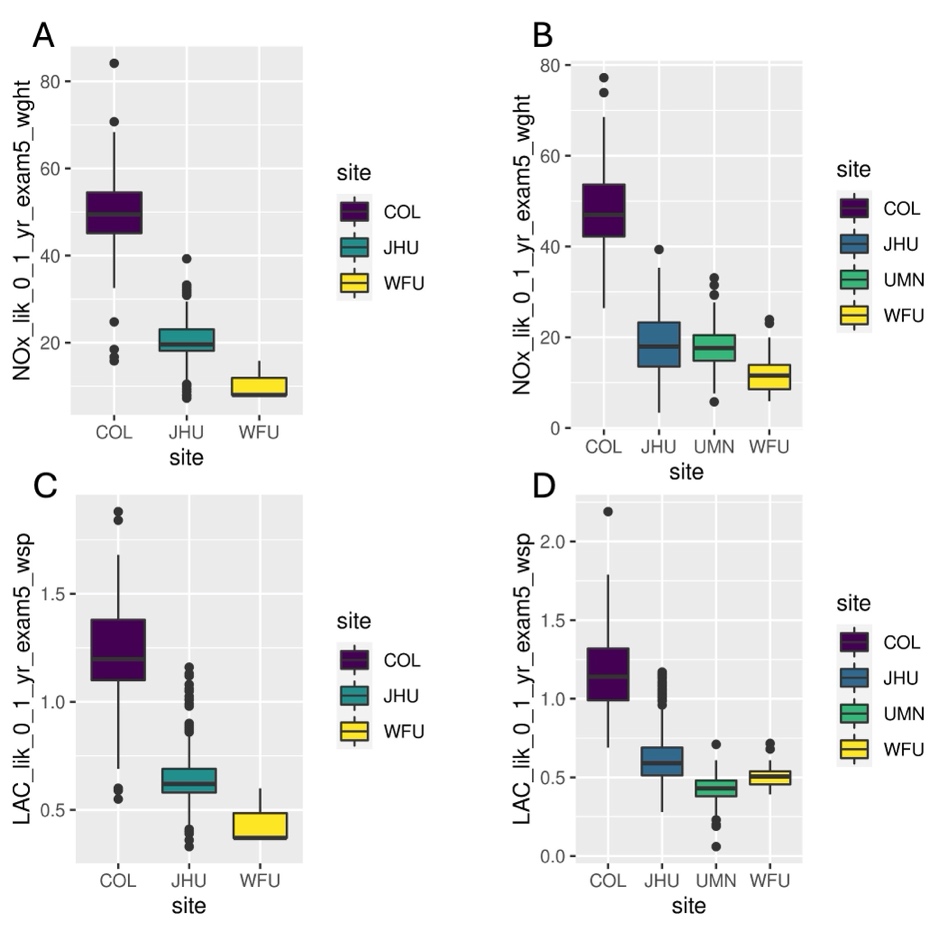


Supplementary Figure 1: Air pollution levels by recruitment site in MESA. A and C: Black NH participants. B and D: white NH participants

| **Full cohort analysis effect size replication in subset** | | | | | | | | |
| --- | --- | --- | --- | --- | --- | --- | --- | --- |
| Exposure | top_10 | top_25 | top_50 | top_100 | top_200 | mean | min | max |
| LAC | 0.8 | 0.63 | 0.72 | 0.85 | 0.91 | 0.78 | 0.63 | 0.91 |
| NOx | 0.88 | 0.91 | 0.84 | 0.92 | 0.91 | 0.89 | 0.84 | 0.92 |
| **Subset analysis effect size replication in full cohort** | | | | | | | | |
| Exposure | top_10 | top_25 | top_50 | top_100 | top_200 | mean | min | max |
| LAC | 0.4 | 0.61 | 0.6 | 0.67 | 0.79 | 0.61 | 0.4 | 0.79 |
| NOx | 0.86 | 0.86 | 0.84 | 0.84 | 0.85 | 0.85 | 0.84 | 0.86 |

Supplementary Table 5: correlation between effect sizes for MESA air pollution meta-analyses. They show correlation 1) between the top sites identified in the full cohort EWAS and 2) the top sites identified in the subset EWAS.

### Enrichment for genomic features

When we looked at enrichment of genomic locations of the top 100 sites (using p<0.05 as a threshold), we found that among MBMS Black NH participants, NOx was the only exposure with notable associations, enriched for two chromatin states (bivalent promoter and promotor upstream of transcription start sites) in addition to being located in promoters, CpG islands and CpG island shores, and enrichment for 9 transcription factor binding sites. We did not observe similar associations for black carbon exposure; and we did not observe any striking enrichments in the MBMS white NH participants. In the MBMS meta-analyses, NOx was enriched for two chromatin states (promotor upstream of transcription start sites and promotor downstream of transcription start sites), three genomic regions (being located in promoters, CpG island shores, and 1-5kb upstream of the TSS), and 20 TFBS.

Among MESA Black NH participants, we observe very similar enrichment for LAC and NOx. NOx is enriched for two active chromatin states (transcription regulation and promotor downstream of TSS 1); three genomic locations (CpG islands, CpG shores and 1-5kb upstream of the TSS); and 53 TFBS. LAC is enriched for three chromatin states (transcription regulation, promotor downstream of TSS 1 and promotor upstream of TSS); three genomic locations (CpG islands, CpG shores, and promotors); and 50 TFBS. They overlap 2 chromatin states, 2 genomic locations, and 45 TFBS. The only structural measure with notable enrichments among the MESA Black NH participants is birth in a Jim Crow state, which is enriched for transcription regulation chromatin state, and 5 TFBS.

Among MESA white NH participants, we see slightly different enrichment patterns for air pollution in the full cohort. Enrichment is instead seen for chromatin states related to transcription regulation, and enhancer for LAC. We only observe enrichment for genomic regions for NOx (intergenic CpG islands); LAC and NOx are enriched for a similar set of TFBS. There are some strong enrichments for Jim Crow birth state, which as discussed above was an artefact of air pollution differences. Among MESA Hispanic participants, LAC exposure shows some associations with active genomic regions, enriched for bivalent promotor chromatin states, location in CpG islands, and 15 TFBS. In the MESA full cohort meta-analysis for LAC and NOx we observe enrichment for chromatin states related to transcription regulation; genomic regions related to intergenic CpG islands; and 24 and 18 TFBS. When we restrict MESA to the New York and Baltimore sites, among white NH participants we observe similar chromatin state enrichments; NOx enrichments for CpG island regions; and a reduction in the number of TFBS enrichments for LAC. In the MESA subset meta-analysis we observe LAC and NOx enrichment for chromatin states related to transcription regulation and promotors; location in CpG islands; and for 48 and 41 TFBS.

Notably, genomic feature enrichments for NOx among both MBMS and MESA Black NH participants involved similar genomic locations (CpG islands and shores) and chromatin states (related to promotors), as well as 6 of a possible 9 TFBS; suggesting that this higher-level analysis may illustrate potential overlap of biological mechanisms or genome regulation between the two cohorts, even though association with specific DNAm sites was not observed between the two studies. This similarity of genomic feature enrichments was not apparent for black carbon. However we think this strengthens implications of our findings, given the heterogeneity between the two cohorts.

# References

Battram, T., Yousefi, P., Crawford, G., Prince, C., Sheikhali Babaei, M., Sharp, G., . . . Suderman, M. (2022). The EWAS Catalog: a database of epigenome-wide association studies. *Wellcome Open Res, 7*, 41. doi:10.12688/wellcomeopenres.17598.2

Brondolo, E., Rahim, R., Grimaldi, S., Ashraf, A., Bui, N., & Schwartz, J. (2015). Place of birth effects on self-reported discrimination: Variations by type of discrimination. *Int J Intercult Relat, 49*, 212-222. doi:10.1016/j.ijintrel.2015.10.001

Consortium, E. P. (2012). An integrated encyclopedia of DNA elements in the human genome. *Nature, 489*(7414), 57-74. doi:10.1038/nature11247

Davis, C. A., Hitz, B. C., Sloan, C. A., Chan, E. T., Davidson, J. M., Gabdank, I., . . . Cherry, J. M. (2018). The Encyclopedia of DNA elements (ENCODE): data portal update. *Nucleic Acids Res, 46*(D1), D794-D801. doi:10.1093/nar/gkx1081

Dominguez, T. P., Strong, E. F., Krieger, N., Gillman, M. W., & Rich-Edwards, J. W. (2009). Differences in the self-reported racism experiences of US-born and foreign-born Black pregnant women. *Soc Sci Med, 69*(2), 258-265. doi:10.1016/j.socscimed.2009.03.022

Elliott, H. R., Burrows, K., Min, J. L., Tillin, T., Mason, D., Wright, J., . . . Relton, C. L. (2022). Characterisation of ethnic differences in DNA methylation between UK-resident South Asians and Europeans. *Clin Epigenetics, 14*(1), 130. doi:10.1186/s13148-022-01351-2

Ernst, J., & Kellis, M. (2015). Large-scale imputation of epigenomic datasets for systematic annotation of diverse human tissues. *Nat Biotechnol, 33*(4), 364-376. doi:10.1038/nbt.3157

Houseman, E. A., Accomando, W. P., Koestler, D. C., Christensen, B. C., Marsit, C. J., Nelson, H. H., . . . Kelsey, K. T. (2012). DNA methylation arrays as surrogate measures of cell mixture distribution. *BMC Bioinformatics, 13*, 86. doi:10.1186/1471-2105-13-86

Krieger, N., Kosheleva, A., Waterman, P. D., Chen, J. T., & Koenen, K. (2011). Racial discrimination, psychological distress, and self-rated health among US-born and foreign-born Black Americans. *Am J Public Health, 101*(9), 1704-1713. doi:10.2105/AJPH.2011.300168

Krieger, N., Waterman, P. D., Kosheleva, A., Chen, J. T., Carney, D. R., Smith, K. W., . . . Samuel, L. (2011). Exposing racial discrimination: implicit & explicit measures--the My Body, My Story study of 1005 US-born black & white community health center members. *PLoS One, 6*(11), e27636. doi:10.1371/journal.pone.0027636

Liu, Y., Ding, J., Reynolds, L. M., Lohman, K., Register, T. C., De La Fuente, A., . . . Hoeschele, I. (2013). Methylomics of gene expression in human monocytes. *Hum Mol Genet, 22*(24), 5065-5074. doi:10.1093/hmg/ddt356

Roadmap Epigenomics, C., Kundaje, A., Meuleman, W., Ernst, J., Bilenky, M., Yen, A., . . . Kellis, M. (2015). Integrative analysis of 111 reference human epigenomes. *Nature, 518*(7539), 317-330. doi:10.1038/nature14248

Sheffield, N. C., & Bock, C. (2016). LOLA: enrichment analysis for genomic region sets and regulatory elements in R and Bioconductor. *Bioinformatics, 32*(4), 587-589. doi:10.1093/bioinformatics/btv612

Watkins, S. H., Ho, K., Testa, C., Falk, L., Soule, P., Nguyen, L. V., . . . Relton, C. (2022). The impact of low input DNA on the reliability of DNA methylation as measured by the Illumina Infinium MethylationEPIC BeadChip. *Epigenetics, 17*(13), 2366-2376. doi:10.1080/15592294.2022.2123898

Zhou, W., Laird, P. W., & Shen, H. (2017). Comprehensive characterization, annotation and innovative use of Infinium DNA methylation BeadChip probes. *Nucleic Acids Res, 45*(4), e22. doi:10.1093/nar/gkw967
